# Anatomy of the dinosaur *Pampadromaeus barberenai* (Saurischia—Sauropodomorpha) from the Late Triassic Santa Maria Formation of southern Brazil

**DOI:** 10.1371/journal.pone.0212543

**Published:** 2019-02-20

**Authors:** Max Cardoso Langer, Blair Wayne McPhee, Júlio César de Almeida Marsola, Lúcio Roberto-da-Silva, Sérgio Furtado Cabreira

**Affiliations:** 1 Laboratório de Paleontologia, FFCLRP, Universidade de São Paulo, Ribeirão Preto-SP, Brazil; 2 Museu de Ciências Naturais, Universidade Luterana do Brasil, Canoas-RS, Brazil; University of Vienna, AUSTRIA

## Abstract

Sauropodomorphs are the most abundant and diverse clade of Triassic dinosaurs, but the taxonomy of their earliest (Carnian) representatives is still poorly understood. One such taxon is *Pampadromaeus barberenai*, represented by a nearly complete disarticulated skeleton recovered from the upper part of the Santa Maria Formation of Rio Grande do Sul, Brazil. Here, the osteology of *Pam*. *barberenai* is fully described for the first time. Detailed comparisons with other Carnian sauropodomorphs reveal a unique anatomy, corroborating its status as a valid species. Potential autapomorphies of *Pam*. *barberenai* can be seen in the articulation of the sacral zygapophyses, the length of the pectoral epipodium, the shape of the distal articulation of the femur and the proximal articulation of metatarsal 1. A novel phylogenetic study shows that relationships among the Carnian sauropodomorphs are poorly constrained, possibly because they belong to a “zone of variability”, where homoplasy abounds. Yet, there is some evidence that *Pam*. *barberenai* may nest within Saturnaliidae, along with *Saturnalia tupiniquim* and *Chromogisaurus novasi*, which represents the sister group to the larger sauropodomorphs, i.e. Bagualosauria.

## Introduction

In all phylogenetic reconstructions of Dinosauria [1–3] (but see [4–6]) the saurischian branch is by far the most speciose during Triassic times. In its traditional understanding, it includes various neotheropods [7], the entire diversity of Herrerasauria [8], and several other possible members of the theropod line [1, 3, 9–10]. However, most recent hypotheses suggest that the majority of known Triassic taxa are nested within the sauropodomorph branch, including a plethora of Norian-Rhaetian forms [11], plus a handful of Carnian taxa from South America [12]. Despite this richness, the first radiation of Sauropodomorpha is still poorly known, partially due to uncertainties regarding the relationships and alpha taxonomy of those same Carnian taxa [13]. Among these, *Pampadromaeus barberenai* Cabreira, Schultz, Bittencourt, Soares, Fortier, Silva & Langer, 2011 [14] was briefly described based on a partial skeleton from the upper part of the Santa Maria Formation [15–16], south Brazil. Together with *Eoraptor lunensis*, *Saturnalia tupiniquim*, *Chromogisaurus novasi*, *Buriolestes schultzi*, and *Panphagia protos*, *Pam*. *barberenai* forms the basal stock from which all later sauropodomorphs evolved, and the full understanding of its anatomy is critical to untangle the early evolution of the group. Therefore, this publication provides a detailed anatomical description of *Pam*. *barberenai*, discussing its implications for the taxonomy of Carnian sauropodomorphs.

## Material and methods

ULBRA-PVT016 was collected during 2004 by a team from Universidade Luterana do Brasil led by SFC and LRS, who also mechanically prepared the specimen at the same institution using non-pneumatic dental mallets, needles, small chisels, and pointers to remove the matrix. Fossil bones were consolidated using cellulose acetate dissolved at 3% in acetone. Most of the skeletal remains were preserved scattered over a single mudstone block [14], but assorted bones were also collected from its perimeter. This includes various tail vertebrae, the right humerus, ulna, and ilium, both tibiae, along with some metatarsals and one pedal phalanx. Based on the corresponding size and preservation of most elements and the absence of duplicated bones or bones obviously attributable to other taxa within the block, most elements included within the specimen are believed to belong to a single individual. The assemblage also includes a handful of bones that could not be anatomically identified, as well as an isolated, 6 cm long metatarsal. The latter bone does not fit the size/shape expected for a metatarsal belonging to the individual represented by the other elements referred to ULBRA-PVT016 and is tentatively excluded from the holotype of *Pam*. *barberenai*. Some of the recovered bones suffered compression in different directions, hampering the comparison of their dimensions, whereas others preserve holes and borings likely made by osteophagous insects [17]. Various bones originally preserved in the main block were extracted, some after being braced in either polyacrylate resin (e.g. the partially articulated skull, sacrum, and left ilium) or a mini plaster jacket (e.g. right dentary, three articulated tail vertebrae, right ilium, left tibia, and left metatarsal II) for protection. In particular, the partially articulated skull was originally exposed on its medial side. As such, it was mechanically prepared, photographed, and covered in polyacrylate resin prior to extraction from the main block. Its lateral surface was then prepared using the same techniques mentioned above. Most bones were, however, not extracted from the main block, and thus are still partially embedded in the bearing rock, hampering the visualisation of some anatomical details.

The current work employs veterinarian anatomical and directional terms over traditional alternatives (e.g. ‘cranial’/‘rostral’ and ‘caudal’ rather than ‘anterior’ and ‘posterior’). For the sake of terminological simplicity, when describing directional orientations, the bone is assumed to have been held along the standard horizontal/vertical axes (e.g. scapula and limbs–excepting pedal phalanges–are oriented strictly along the vertical). The term “facial” is applied in the description of the skull bones in reference to surfaces not covered by structures other than the integument, in opposition to “depressed” areas such as those occupied by the narial, antorbital, or temporal fossae.

As for clade names, we employ the branch-based definition of Sauropodomorpha of Fabri et al. [18] as “the largest clade containing *Saltasaurus loricatus* Bonaparte and Powell 1980 [19], but not *Allosaurus fragilis* Marsh 1877 [20] nor *Iguanodon bernissartensis* Boulenger in Beneden 1881 [21]”. Although Sauropodomorpha was first phylogenetically defined in a node-based fashion [22–23], the above branch-based definition better fits the mainstream [24–27] current usage of the term.

The following anatomical description is based on the first-hand observation of ULBRA-PVT016 by all authors. It was compared in greater detail to the other Carnian sauropodomorphs, i.e. *Bagualosaurus agudoensis*, *Bu*. *schultzi*, *C*. *novasi*, *E*. *lunensis*, *Pan*. *protos*, and *Sa*. *tupiniquim*, all of which were studied first-hand by at least one of the authors (Table 1). Apart from the literature sources used for comparative purposes [12, 28–35], photographs of those taxa taken by the authors and/or supplied by Jonathas Bittencourt complement the information provided here.

**Table 1:** Source of anatomical information for Carnian Sauropodomorpha employed in this study.

*Bagualosaurus agudoensis*	Direct observation of UFRGS-PV-1099-T by MCL during April 2015 and by BWM during November 2017. [12].
*Buriolestes schultzi*	Direct observation of ULBRA-PVT280 by MCL during October 2013. [5,35]. Photographs of ULBRA-PVT280 taken by MCL during October 2013.
*Chromogisaurus novasi*	Direct observation of PVSJ-845 by JCAM during March 2015. [31–32]. Photographs of PVSJ-845 taken by Jonathas Bittencourt during April 2011 and by JCAM during March 2015.
*Eoraptor lunensis*	Direct observation of PVSJ-512 by MCL during January 2000 and September 2011, by JCAM during March 2015, and by BWM during May 2017. [34]. Photographs of PVSJ-512 taken by MCL during January 2000 and September 2011, by Jonathas Bittencourt during April 2011, and by JCAM during March 2015.
*Panphagia protos*	Direct observation of PVSJ-874 by MCL during September 2011, by JCAM during March 2015, and by BWM during May 2017. [30, 33]. Photographs of PVSJ-874 taken by MCL during September 2011, by Jonathas Bittencourt during April 2011, and by JCAM during March 2015.
*Saturnalia tupiniquim*	Direct observation of MCP-3944, 3845, and 3846 by MCL from 1998–2017 and by JCAM from 2014–2017. [28–29]. Photographs of MCP-3944 and 3845 taken by JCAM during August 2014, and by Jonathas Bittencourt during December 2010.

### Phylogenetic methods

The phylogenetic position of *Pam*. *barberenai* was investigated based on the taxon-character matrix of Bronzati *et al*. [36], which incorporates previous modifications by several authors [37–44] to the Yates [45] original dataset. The “Oxford braincase” was excluded from the data-matrix and, after Bronzati *et al*. [36], character 86 was also excluded. The matrix was further modified with new and independent scorings of the Carnian taxa *Sa*. *tupiniquim*, *E*. *lunensis*, *Pan*. *protos*, and *C*. *novasi*, and the inclusion of three taxa: *Pam*. *barberenai*, *Bu*. *schultzi*, and *Ba*. *agudoensis*. In addition, based on the comparison to those seven taxa conducted during the elaboration of the extended diagnosis of *Pam*. *barberenai*, and incorporating characters discussed by various authors [5, 12, 31, 35], a set of 43 extra characters were added (see S1 File) to the taxon-character matrix of Bronzati *et al*. [36]. In this process, characters 13, 114, 258, and 373 of the original matrix were excluded and respectively replaced by characters 373–374, 395–396, 408, and 387 of the modified matrix. The resulting data matrix has 57 taxa and 413 characters (see S2 File), a parsimony analysis of which was conducted in TNT [46] via a heuristic search with the following parameters: 5,000 replicates of Wagner Trees, hold 10, and tree bi‐section and reconnection (TBR) for branch swapping. The most parsimonious trees (MPTs) found in this first analysis were subjected to a second round of TBR, resulting in 192 MPTs of 1.467 steps (CI = 0.325; RI = 0.673). Clade support was measured using resampling techniques [47] and decay indices [48–49]. Frequency difference (CG) bootstrap values were computed in TNT [46], based on 1,000 pseudoreplicates. “Bremer support” was calculated with the appropriate TNT script.

### Fieldwork permit and repository information

All necessary permits were obtained for the described study, which complied with all relevant Brazilian regulations. As requested in the ordinance number 4.146 from March 4th, 1942, the field work and fossil collection permit was issued by the Departamento Nacional de Producão Mineral (DNPM), in the given case (the permit, dated from August 13th 2004, has no number) by the head of the 1st DNPM district in Rio Grande do Sul, Sérgio Bizarro Cesar, allowing the research to be conducted by the private Brazilian university Univesidade Luterana do Brasil (ULBRA), where the specimen described in this work is permanently deposited and accessible to other researchers, under the number ULBRA-PVT016, in the collection of the Museu de Ciências Naturais, Av. Farroupilha 8001, Canoas 92425–900, Rio Grande do Sul, Brazil.

### Institutional abbreviations

CAPPA/UFSM, Centro de Apoio à Pesquisa Paleontológica da Quarta Colônia da Universidade Federal de Santa Maria, São João do Polêsine-RS, Brazil; MCP, Museu de Ciências e Tecnologia, Pontifícia Universidade Católica do Rio Grande do Sul, Porto Alegre-RS, Brazil; PVSJ, Divisíon de Paleontologia de Vertebrados del Museo de Ciencias Naturales y Universidad Nacional de San Juan, San Juan, Argentina; ULBRA, Museu de Ciências Naturais, Universidade Luterana do Brasil, Canoas-RS, Brazil.

## Systematic palaeontology

**DINOSAURIA** Owen, 1842 [50]

**SAURISCHIA** Seeley, 1887 [51]

**SAUROPODOMORPHA** Huene, 1932 [52]

cf. **SATURNALIIDAE** (nom. trans. ex Saturnaliinae Ezcurra, 2010 [31])

Definition: Saturnaliidae represents the maximal sauropodomorph clade to encompass *Sa*. *tupiniquim* Langer, Abdala, Richter & Benton, 1999 [53], but not *Plateosaurus engelhardti* von Meyer, 1837 [54].

***PAMPADROMAEUS BARBERENAI*** Cabreira, Schultz, Bittencourt, Soares, Fortier, Silva & Langer, 2011 [14]

Holotype: ULBRA-PVT016 [14], disarticulated partial skeleton including a semi-articulated cranium set with right premaxilla, maxilla, lacrimal, left palatine, and an indeterminate partial palatal bone; skull bones including right frontal, prefrontal, postorbital, and pterygoid, left nasal, parietal, jugal, squamosal, quadrate, and pterygoid; nearly complete left dentary, with possible portions of the angular and surangular; partial right dentary; semi-articulated set of postdentary bones of the right lower jaw including, angular, surangular, articular, and prearticular; left prearticular; vertebrae including atlas/axis complex, third neck vertebra, eleven trunk vertebrae, articulated pair of sacral vertebrae and ribs, and 17 tail vertebrae; various neck and trunk ribs and haemal arches; partial left scapula; right scapula, humerus, and ulna; partial ilia; proximal portion of the left ischium; femora, tibiae (badly preserved), and fibulae; left metatarsals I and II, partial right metatarsal II, partial metatarsals III, and right metatarsal IV; two (probably pedal) phalanges.

Referred material: Two nearly complete left femora–CAPPA/UFSM 0027 [55] and 0028 [56]–were referred to *Pam*. *barberenai*, the latter probably representing a juvenile individual. Although we agree with Muller *et al*. [55–56] that, given the matching morphology and provenance, *Pam*. *barberenai* is the most likely attribution for those specimens, we refrain from including them in the current anatomical account. This is because (1) they were already described in detail in the above-mentioned papers, and (2) there is a pair of reasonably well-preserved femora in ULBRA-PVT016. Hence, we prefer to restrict anatomical description and cladistic scoring to the holotype of *Pam*. *barberenai*. We feel that the small amount of additional information provided by those two isolated femora does not compensate the risk of including rogue phylogenetic data derived from remains potentially not belonging to the taxon.

Locality and horizon: Janner site (53°17′34.20″ W, 29° 39′10.89″ S), Agudo municipality, Rio Grande do Sul, Brazil [14]; uppermost part of the Alemoa Member of the Santa Maria Formation, i.e. lower part of the highstand systems tract of the “Santa Maria 2” sequence of Zerfass *et al*. [57] = Candelária Sequence of Horn *et al*. [16]. A late Carnian (c. 233 Ma) U-Pb radioisotopic age has been assigned to mudstones of the site known as Waldsanga [58], which are equivalent in terms of lithology and sequence stratigraphy context to those of the Janner site. Yet, whereas the Waldsanga fauna is dominated by the rhynchosaur *Hyperodapedon*, the cynodont *Exaeretodon* is the most common taxon at the Janner site [12]. A replacement of *Hyperodapedon* by *Exaeretodon* has been postulated as occurring between the lower third and upper portions of the Ischigualasto Formation [3, 59], suggesting that the Janner site fauna may be somewhat younger than that of the Waldsanga site. *Pam*. *barberenai* and *Ba*. *agudoensis* may be, therefore, somewhat younger than other Santa Maria Formation dinosaurs, such as *Sa*. *tupiniquim*, *Bu*. *schultzi*, and *Staurikosaurus pricei*.

Diagnosis: A small (around 1.5 m long) sauropodomorph dinosaur that differs from all other early members of the group and Carnian dinosauromorphs, for which the corresponding anatomical parts are known, by the following unique traits: partially fused zygapophyses in the primordial sacral pair; ulna longer than 80 per cent the humeral length (but see description of the ulna for a discussion on their association); intercondylar groove of the femur broader lateromedially than both the lateral and medial condyles; metatarsal 1 with an L-shaped proximal outline, including a lateral expansion that covers part of the cranial surface of metatarsal II. In addition, *Pam*. *barberenai* can be distinguished from all other Carnian sauropodomorphs as summarized below, except for *Ba*. *agudoensis*, the distinctiveness of which relative to *Pam*. *barberenai* has been recently reviewed [12].

*Eoraptor lunensis*, from the Ischigualasto Formation, Argentina (PVSJ 512 = *; PVSJ 559 = †; [34, 60]), differs from *Pam*. *barberenai* in the following features: elongated dorsomedial ramus of the caudal premaxillary process *; base of the dorsal ramus of the maxilla lacks a large rostrally opening lateral foramen *; no promaxillary fossa *; rounded ridge forming the ventral margin of the antorbital fossa *; web of bone spans rostroventrally from the junction between rostral and ventral rami of lachrymal to laterally overlap the dorsocaudal corner of the antorbital fenestra *; raised lip forming the surangular margin of internal mandibular fenestra *; set of anterior foramina at the lateral surface of the dentary, below the first three teeth *; first premaxillary tooth with denticles *; less than 20 maxillary teeth *; main dentary and maxillary tooth crowns with concave distal margin, denticles set perpendicular to the tooth margin, and not restricted to their apical part *; pterygoid with a transverse row of palatal teeth *; first dentary tooth inset from the rostral margin of the bone and not mesiodistally compressed *; short scapular blade relative to its minimal craniomedial breadth *; dorsal margin of the acromion process forms a low angle to the cranial margin of the scapular blade *; distal end of the humerus less than one third the bone length *; brevis shelf closely connected to the supracetabular crest *; femur with non-hypertrophied fibular condyle †; tibia with well-developed fibular crest †; metatarsal IV with a broader than deep distal outline*.

*Panphagia protos*, from the Ischigualasto Formation, Argentina (PVSJ 874; [30,33]), differs from *Pam*. *barberenai* in the following features: parietal with a reduced rostrolateral process; enlarged quadrate foramen; first dentary tooth inset from the rostral margin of the bone and not mesiodistally compressed; main maxillary and dentary tooth crowns with small denticles; short scapular blade relative to its minimal craniocaudal breadth and with the dorsal margin more expanded in that same direction; pubic peduncle of the ilium with a rounded dorsal margin; caudal end of the brevis shelf not projecting ventral to the “posteromedial shelf”.

*Chromogisaurus novasi*, from the Ischigualasto Formation of Argentina (PVSJ 845; [31–32]), differs from *Pam*. *barberenai* in the following features: enlarged olecranon process of the ulna; a more strongly concave ventral margin of the iliac medial acetabular wall; supracetabular crest reaches the distal end of the pubic peduncle; non-hypertrophied fibular condyle in the femur; tibia with well-developed fibular crest; fibula lacks a rugose iliofibularis muscle insertion.

*Saturnalia tupiniquim*, from the Santa Maria Formation, Brazil (MCP-3844PV = *; 3945PV = †; 3846PV = ‡; [28–29, 61]), differs from *Pam*. *barberenai* in the following features: skull shorter than 2/3 the femoral length †; shorter frontal, less than four times its minimal breadth and with the orbital fossa reaching the rostral margin of the bone in the ventral surface †; less than 18 dentary teeth†; main maxillary and dentary tooth crowns with small denticles set perpendicular to the tooth margin †; caudal margin of the scapular blade concave along its entire length *†; enlarged olecranon process of the ulna *; supracetabular crest reaches the distal end of the pubic peduncle *‡; femur with non-hypertrophied fibular condyle *†‡; better developed fibular crest in the tibia *†‡; distal articulation of metatarsal I lateromedially broader than dorsoventrally deep *†.

*Buriolestes schultzi*, from the Santa Maria Formation, Brazil (ULBRA-PVT 280 = *; CAPPA/UFSM 0035 = †; [5, 35]), differs from *Pam*. *barberenai* in the following features: premaxilla with a second anterior foramen and a caudoventrally directed ventromedial ramus of the caudal process *; clear-cut rounded excavation at the caudal margin of the premaxillary alveolar margin, indicating the presence of a subnarial gap *†; no promaxillary fossa *†; web of bone spanning rostroventrally from the junction between rostral and ventral rami of lachrymal to laterally overlap the dorsocaudal corner of the antorbital fenestra †; bone sheet between the rostral and ventral processes of the prefrontal †; ventral ramus of the postorbital much broader than the caudal ramus †; rostral and caudal rami of the jugal forming angle of more than 180° *†; forked portion of the caudal ramus of the jugal does not reach base of the dorsal ramus *†; main dentary and maxillary tooth crowns with concave distal margin, small denticles not restricted to their apical part and set perpendicular to the tooth margin *†; first dentary tooth not mesiodistally compressed *†; axis with dorsoventrally deeper neural arch and sigmoid dorsal margin of the neural spine †; dorsocaudally directed neural spine of the second primordial sacral vertebra*; distal end of the humerus less than one third of the bone length *; more laterally expanded supracetabular crest *†; pubic peduncle of the ilium with a rounded dorsal margin *†; caudal end of the brevis shelf does not project further ventral than the “posteromedial shelf” *†; femoral head with well-developed medial tubercle *†; tibia with well-developed fibular crest †.

## Description of ULBRA-PVT016

### Skull

Most of the skull bones are preserved scattered within the block, except for the semi-articulated right premaxilla, maxilla, lacrimal, and left palatine. The quadratojugal is the only dermal bone of the skull cover not present in the assemblage, whereas the palate did not preserve either vomer (but see the description of the palatine below) or ectoperygoids. No braincase elements were found.

#### Premaxilla (Figs 1 and 2)

The right premaxilla is preserved semi-articulated to the right maxilla, as part of a skull piece that also includes the right lacrimal, the left palatine, and an undetermined palatal bone. The lateral surface is currently the only exposed surface, whereas the medial surface is concealed by the resin armature used to hold the element for preparation. Yet, the anatomy of this surface was recorded by photographs taken when it was exposed. The bone is composed of a subtriangular, lateromedially compressed body, which forms the rostroventral tip of the skull, a dorsal process, and a bifurcating caudal process. Laterally, the dorsal portion of the dorsolateral ramus of the caudal process, the ventral portion of the dorsal process, and the dorsocaudal portion of the body are marked by the narial fossa. It deeply excavates both processes and extends as a feeble shallower depression over about half of the rostrocaudal length of the premaxillary body, where it lacks marked borders and merges smoothly with the facial surface of the bone. The lateral surface of the body is highly fractured, but the “anterior premaxillary foramen” of Sereno *et al*. [34] can be seen at its rostral tip, just dorsal to the first tooth. The thin dorsal process extends dorsocaudally, forming an arch to meet the nasal, but the disarticulation of these bones (and incomplete preservation of the latter) precludes observation of their interrelationships. Its tip is preserved in original position, but disconnected by a missing part from the rest of the process (Fig 2B). The medial surface of the premaxillary body is badly preserved, but a striated area rostral to the narial opening, extending onto the base of the dorsal process, probably represents the articular surface for the opposite element. The caudal process bifurcates into subparallel dorsolateral and ventromedial rami. The former extends as a straight wedge dorsal and lateral to the rostral ramus of the maxilla but deflects dorsally forming a narrow strip (Fig 2). This indicates that the premaxilla extended along the entire ventral margin of the external naris, although it probably terminated prior to the nasal so that the maxilla would have contributed to the caudal margin of the external naris. The ventromedial ramus is significantly displaced ventrally relative to the dorsolateral ramus, projecting caudally as an extension of the lingual alveolar margin of the premaxilla. It fits medial to the rostral ramus of the maxilla, whereupon the latter is embraced by both rami of the caudal premaxillary process. Starting rostrally as a more rod-like structure, the ventromedial ramus is dorsomedially flattened for most of its length. Its smooth dorsal surface receives the palatal process of the maxilla, which extends into a slot-shaped depression positioned medially between both caudal rami of the premaxilla. Dorsal to this depression, a foramen is present at the base of the dorsolateral ramus. No lateral continuation of this foramen is seen, but this may be concealed by fractures on the lateral surface of the bone.

**Fig 1:**
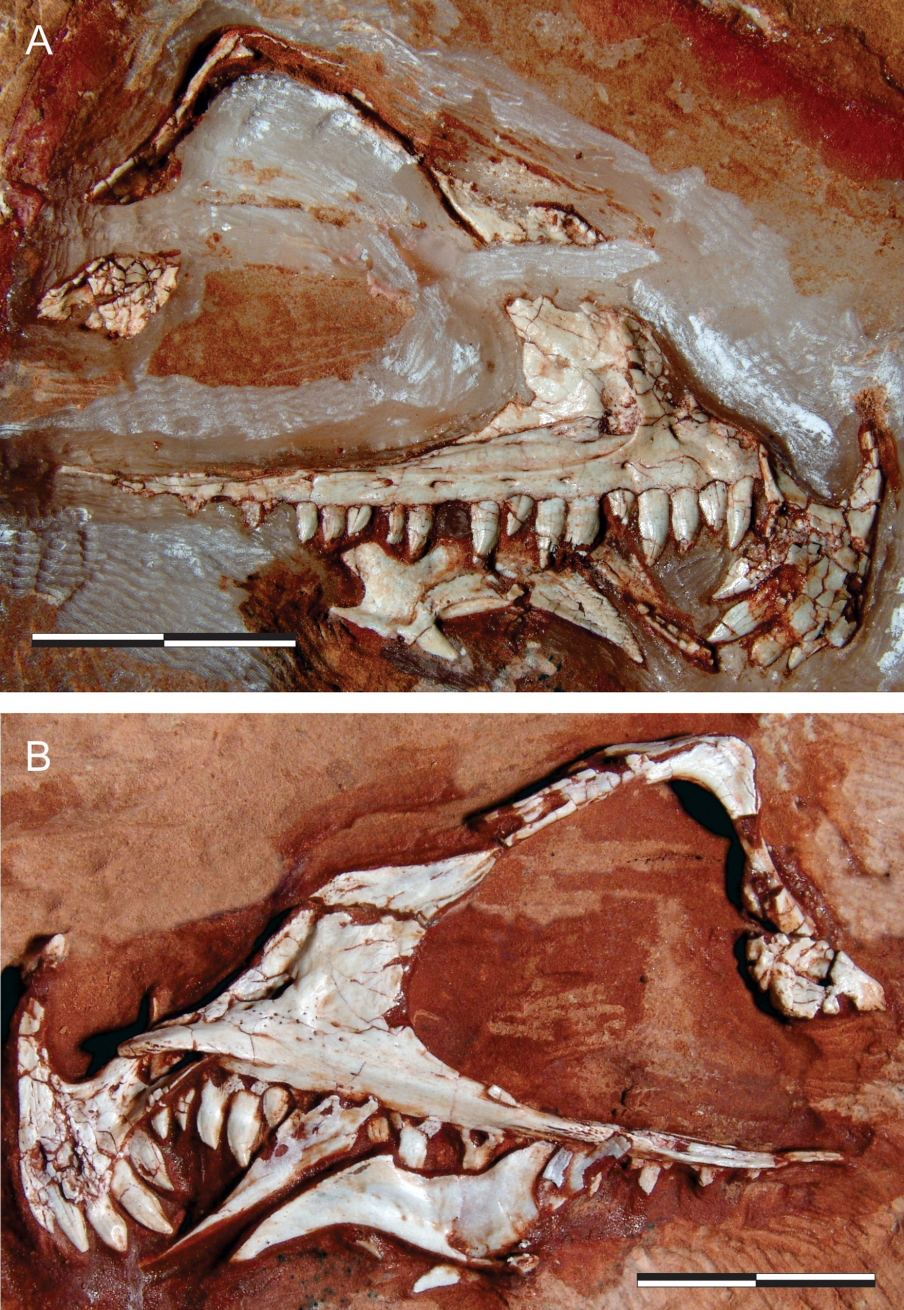
*Pampadromaeus barberenai* (ULBRA-PVT016), skull. Right articulated front part of the skull in A, lateral; and B, medial views. Scale bar = 2 cm.

**Fig 2:**
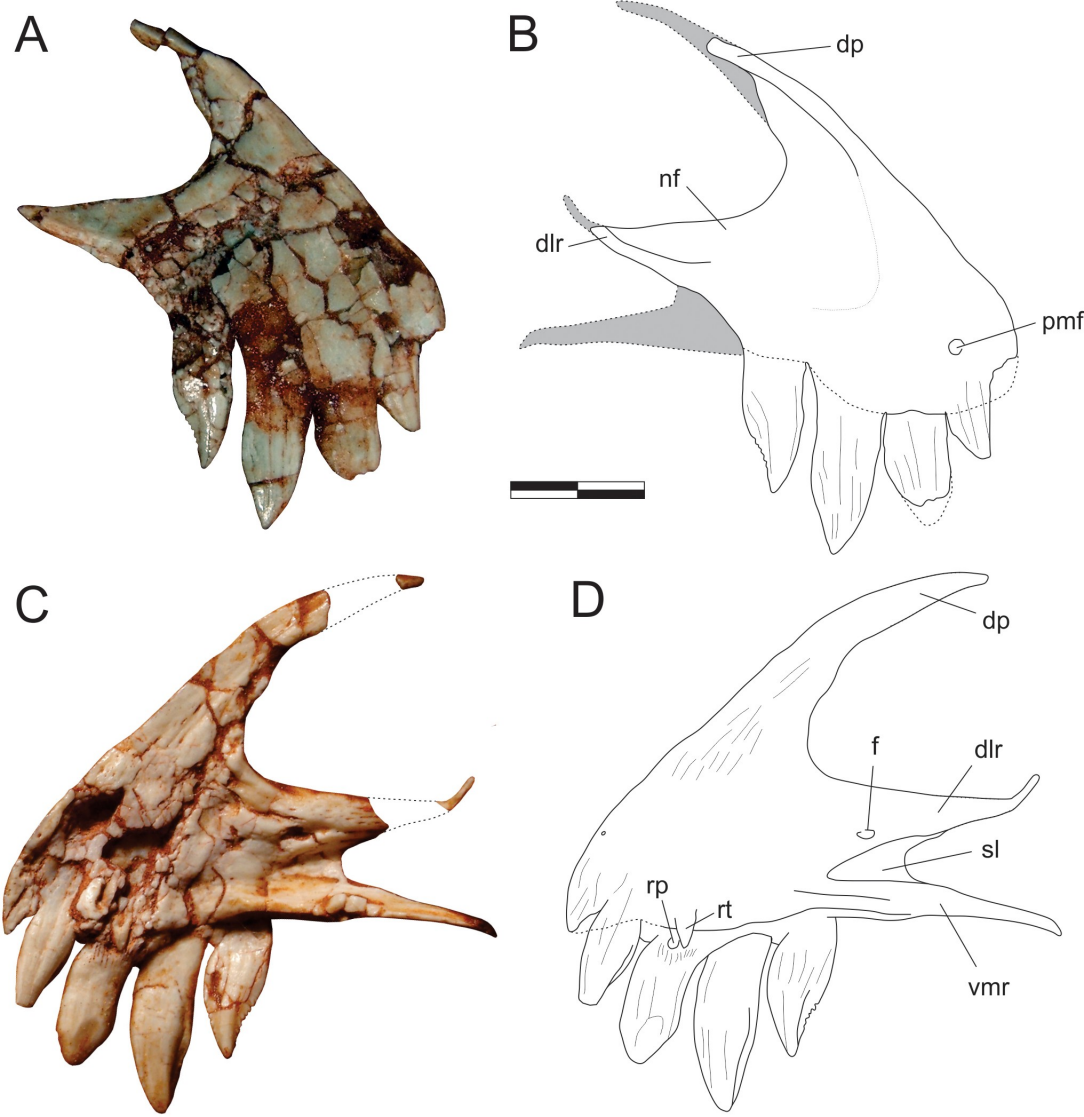
*Pampadromaeus barberenai* (ULBRA-PVT016), premaxilla. Photographs (A,C) and drawings (B,D) of the right premaxilla in A-B, lateral; and C-D, medial views. Abbreviations: dlr, dorsolateral ramus of the caudal process; dp, dorsal process; f, foramen; nf, narial fossa; pmf, anterior premaxillary foramen; rp, replacement pit; rt, replacement tooth; sl, slot; vmr, ventromedial ramus of the caudal process. Dashed lines and grey areas indicate inferred skeletal margins and surfaces. Scale bar = 5 mm.

Digitally reconstructing the premaxilla-maxilla articulation demonstrates that there is no space for a large diastema between the last premaxillary and the first maxillary tooth. Furthermore, as the rostral tip of the maxillary alveolar margin is not dorsally deflected as in forms with a well-developed subnarial gap [62], we assume that the latter feature is absent in *Pam*. *barberenai*. Nonetheless, when the maxillary palatal process is articulated with its receiving slot on the premaxilla, the alveolar margin of the latter element displays a slight ventral offset of c. 150–160° relative to the maxilla.

#### Maxilla (Figs 1 and 3–5)

**Fig 3:**
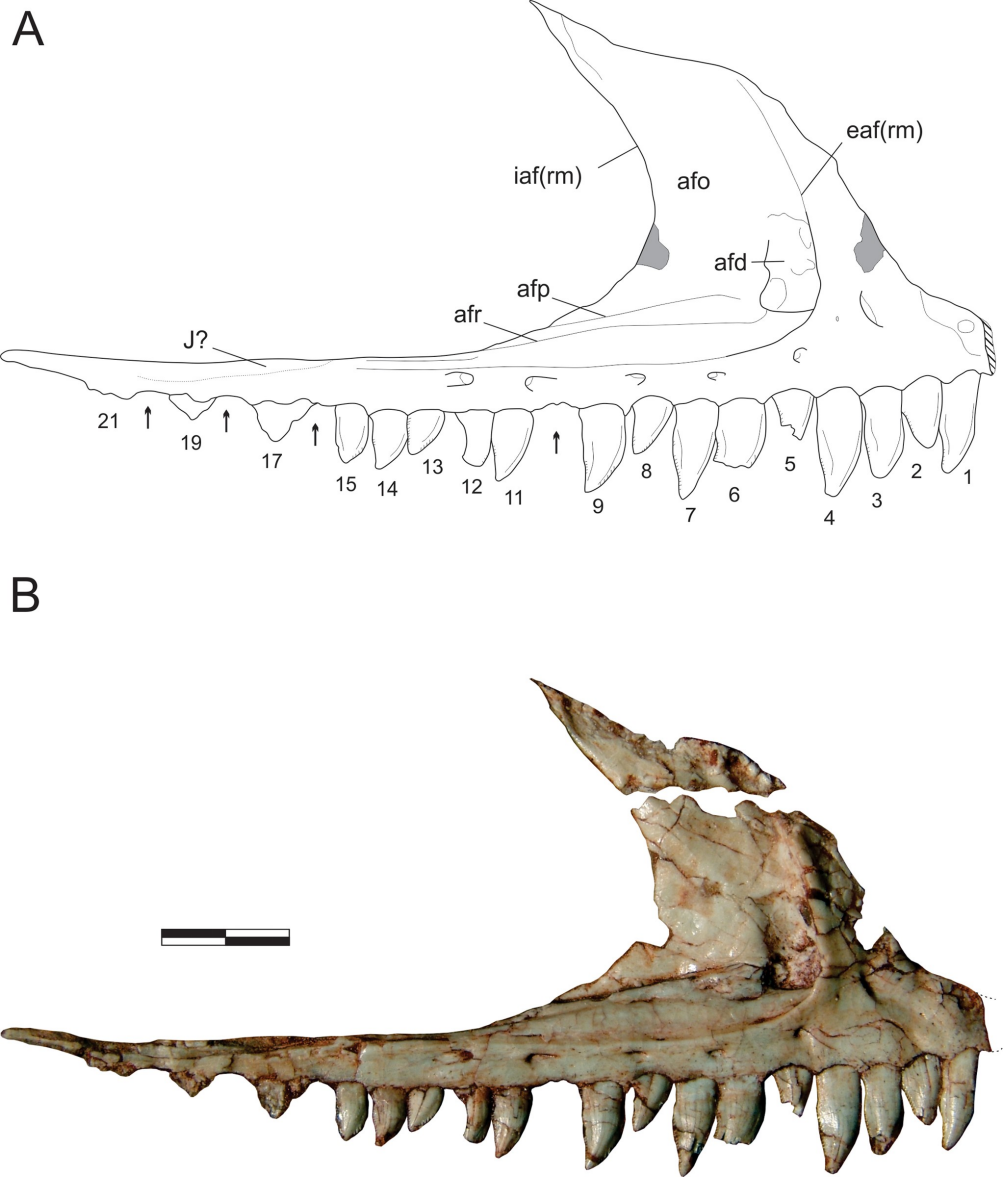
*Pampadromaeus barberenai* (ULBRA-PVT016), maxilla. Drawing (A) and photograph (B) of the right maxilla in lateral view. Abbreviations: afd, antorbital fossa depression; afo, antorbital fossa; afp, antorbital fossa platform; afr, antorbital fossa ridge; eaf(rm), external antorbital fenestra (rostral margin); iaf(rm), internal antorbital fenestra (rostral margin); J?, possible jugal articulation area; 1–21, numbered teeth. Arrows indicate tooth position without exposed teeth; dashed lines indicate inferred skeletal margins; hatched/grey areas indicate broken surfaces. Scale bar = 8 mm.

**Fig 4:**
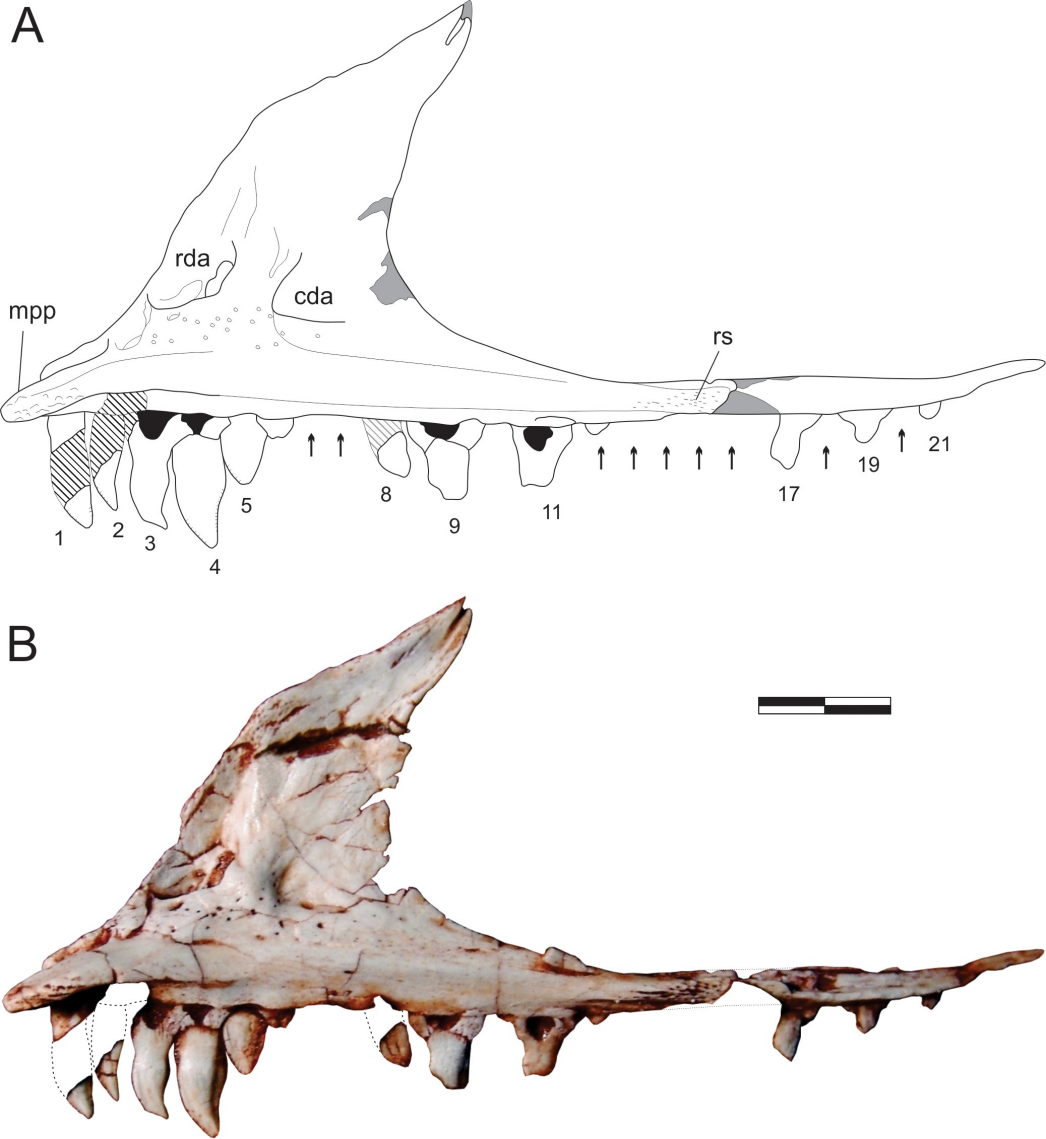
*Pampadromaeus barberenai* (ULBRA-PVT016), maxilla. Drawing (A) and photograph (B) of the right maxilla in medial view. Abbreviations: cda, caudal depressed area; mpp, maxillary palatal process; rda, rostral depressed area; rs, rugose surface; 1–21, numbered teeth. Arrows indicate tooth position without exposed teeth; dashed lines indicate inferred skeletal margins; hatched/grey areas indicate broken surfaces. Scale bar = 8 mm.

The main body of the maxilla is composed of caudal and rostral rami, as well as the dorsal ramus and the palatal process. The lateral surface of the rostral ramus bears a subtle depression at its rostrodorsal corner, which was covered by the dorsolateral ramus of the caudal process of the premaxilla when in articulation. The ovoid aperture in centre of the depression more likely represents a breakage leading to the root of the first preserved tooth than a genuine foramen. The dorsal margin of the rostral ramus of the maxilla is ventrorostrally to dorsocaudally inclined, forming a continuous concave outline (in lateral/medial views) with the rostral margin of the dorsal ramus. The latter extends dorsally and slightly caudally, tapering dorsocaudally at its tip. As currently preserved, the tip of the dorsal ramus of the maxilla is separated from its base (Fig 1); however, it was digitally dragged to its likely natural position (Fig 3) following the medial view contour observable in photographs taken before the breakage (Figs 1B and 4). The tip of the dorsal ramus is not exposed facially, but laterally overlapped the lacrimal, extending inside the antorbital fossa. A longitudinal groove on its medial surface received a ventral crest located on the rostrolateral edge of the lacrimal.

**Fig 5:**
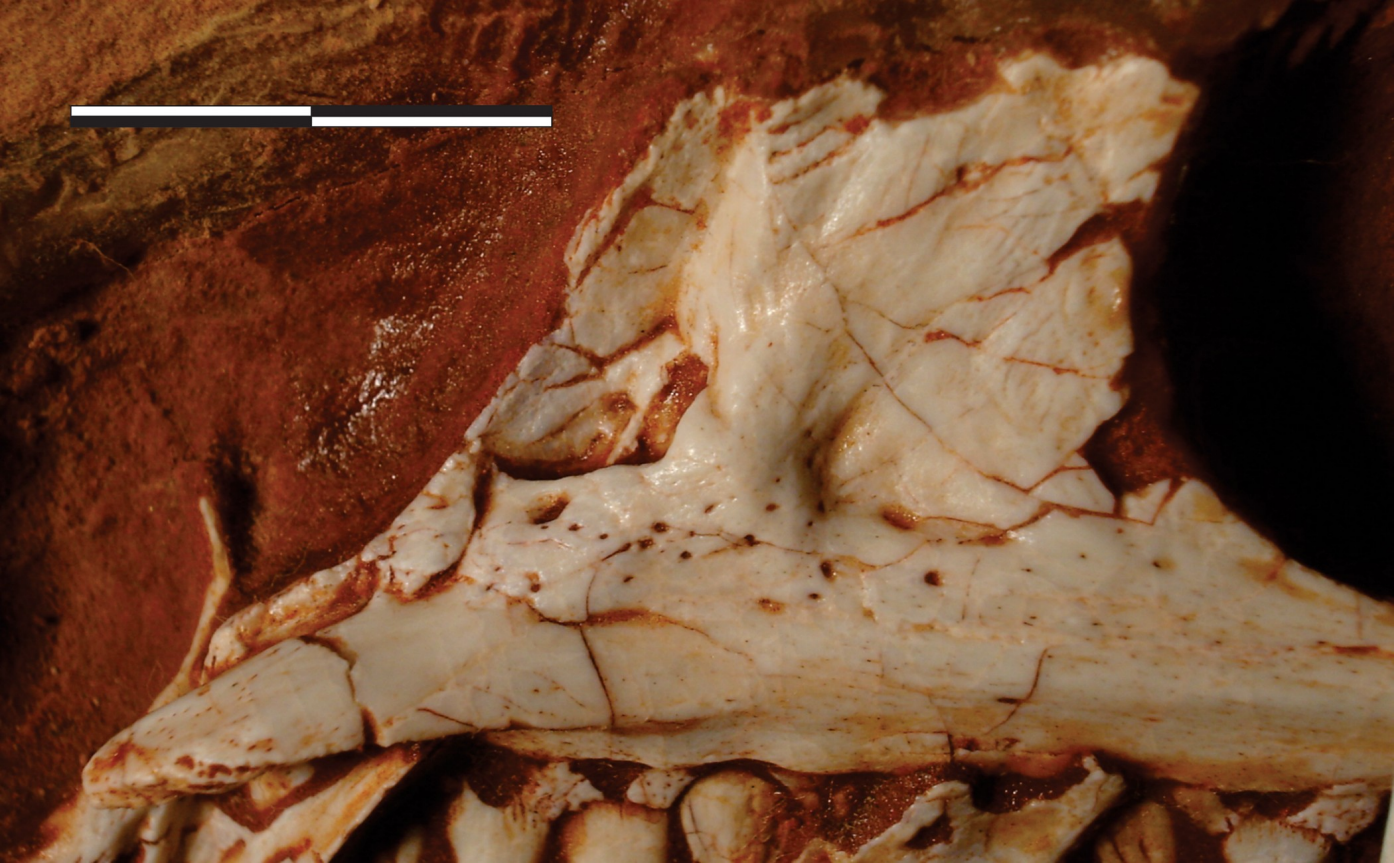
*Pampadromaeus barberenai* (ULBRA-PVT016), maxilla. Detail of the right maxilla in medial view. Scale bar = 1 cm.

The antorbital fossa excavates most of the lateral surface of the dorsal ramus, forming a very steep rostral rim which is especially prominent on its slightly everted ventral half. This rim extends caudally along the caudal ramus of the maxilla, forming the ventral margin of the fossa. It projects slightly laterally, forming a subtle ridge that smoothly merges with the dorsal and ventral bone surfaces. Ventral to that, no other ridges are present on the facial surface of the maxilla, but a secondary ridge (“afr” in Fig 3) extends inside the antorbital fossa, subparallel and dorsal to the ridge that marks the ventral margin of the fossa. This ridge is sharper rostrally, with a smoother caudal half, and a better developed version of which is seen in *B*. *schultzi* (ULBRA-PVT 280). In *Pam*. *barberenai*, it reaches roughly the level of the 11^th^ preserved maxillary tooth crown, at the caudal edge of the medial wall of the antorbital fossa, which thus does not extend the entire caudal length of the antorbital fenestra of the maxilla. Caudal to the termination of the medial wall, the internal and external antorbital fenestrae share a single ventral margin. Above the 13^th^ to 15^th^ maxillary tooth positions, the ventral margin of the fenestra is formed by the main ventral ridge, which sharper rostrally than in this portion. Within the antorbital fossa, dorsomedial to the secondary ridge, there is an inclined (dorsomedial to ventrolateral), narrow platform (“afp” in Fig 3). Medial to this platform lies a groove that delimits the lateral edge of the laminar vertical portion of the medial wall of the antorbital fossa, which expands dorsally and delimits the concave rostral margin of the internal antorbital fenestra. No aperture pierces the medial wall of the antorbital fossa, but a broad depression (“afd” in Fig 3) is visible rostral to the above mentioned narrow platform and dorsal to the rostral portion of the internal ridge. Its dorsal half is relatively smooth compared to the rugose ventral half. Facing that depression, at the base of the caudal surface of the facial portion of the dorsal maxillary ramus there is an area where bone surface gives way to a mixture of poorly preserved bone and sediment, possibly representing a filled connection/channel to the more rostral of the lateral foramina and/or to the aperture on the medial surface of the bone. As mentioned in Cabreira *et al*. [14], this depression is in the same position as the promaxillary fenestra/foramen of many early theropods and may correspond to an incipient version of that.

The facial surface of the maxilla has six well-developed neurovascular foramina. The more rostral of these is slightly larger than the following two. It lies at the base of the dorsal ramus of the bone and opens rostrally/rostroventrally towards the rostral ramus. The other foramina are aligned around the ventral margin of the external antorbital fenestra and get larger as they pass caudally. The last foramen leads to a groove that extends caudally for more than twice its dorsoventral diameter. It is larger than the other foramina, but not as markedly as in *E*. *lunensis* [34]. There is no evidence of a subnarial foramen, as seen in *E*. *lunensis* [34], but this may be obscured by the dorsolateral ramus of the premaxillary caudal process which covers the rostral tip of the maxilla. Caudal to the 15^th^ tooth position, the caudal ramus of the maxilla tappers more abruptly, and it cannot be determined (Fig 3) if that portion of the bone is dorsally overlapped by the rostral ramus of the jugal, as is the case in *E*. *lunensis* [34].

The palatal process is clearly exposed on the medial surface of the maxilla (Figs 4 and 5), and medially parallels the rostral ramus of the bone. It is dorsoventrally flattened, articulating dorsally with the ventromedial ramus of the caudal process of the premaxilla. The rostral tip of the palatal process is ventrally curved. It has a rugose medial surface and laterally articulates with the medial slot between the two caudal rami of the premaxilla. The medioventral margin of the palatal process forms a keel, which continues caudally as the ventral margin of a more lateromedially thick portion of the caudal ramus, including a rugose surface (“rs” in Fig 4) at roughly the level where both the internal and external antorbital fenestra share a single ventral margin. Ventral to that, a slightly less lateromedially expanded part of the bone forms the alveolar margin. The rostral ramus of the maxilla articulates medially with the medioventral ramus of the caudal premaxillary process, slotting between that and the dorsolateral ramus of that process. Its medial surface is obscured in the available photographs by the former ramus and the palatal ramus of the maxilla, precluding the observation of further details. The dorsal ramus of the maxilla has a rather complex medial surface, with depressed areas on the rostral and caudal ends of its ventral portion (“rda” and “cda” in Figs 4 and 5). The latter has a deep, right-angled rostroventral corner and nearly mirrors in shape the antorbital fossa and its rugose depression on the lateral surface of the maxilla. The rostral depression is a broad pocket that probably opens laterally towards the rostral wall of the antorbital fossa. That depressed area is much deeper near its right-angled caudoventral corner, and shallower dorsorostrally. Both depressions are ventrally bound by a rugose, medially bulging area that is penetrated by several vascular apertures; a particularly large one is located ventral to the rostral pocket. This is continuous to an equally rugose and medially bulging pillar-like structure that extends dorsally between the depressions. This structure flattens and expands rostrocaudally as it proceeds dorsally, including a sharper rostral ridge.

#### Nasal (Fig 6A and 6B)

**Fig 6:**
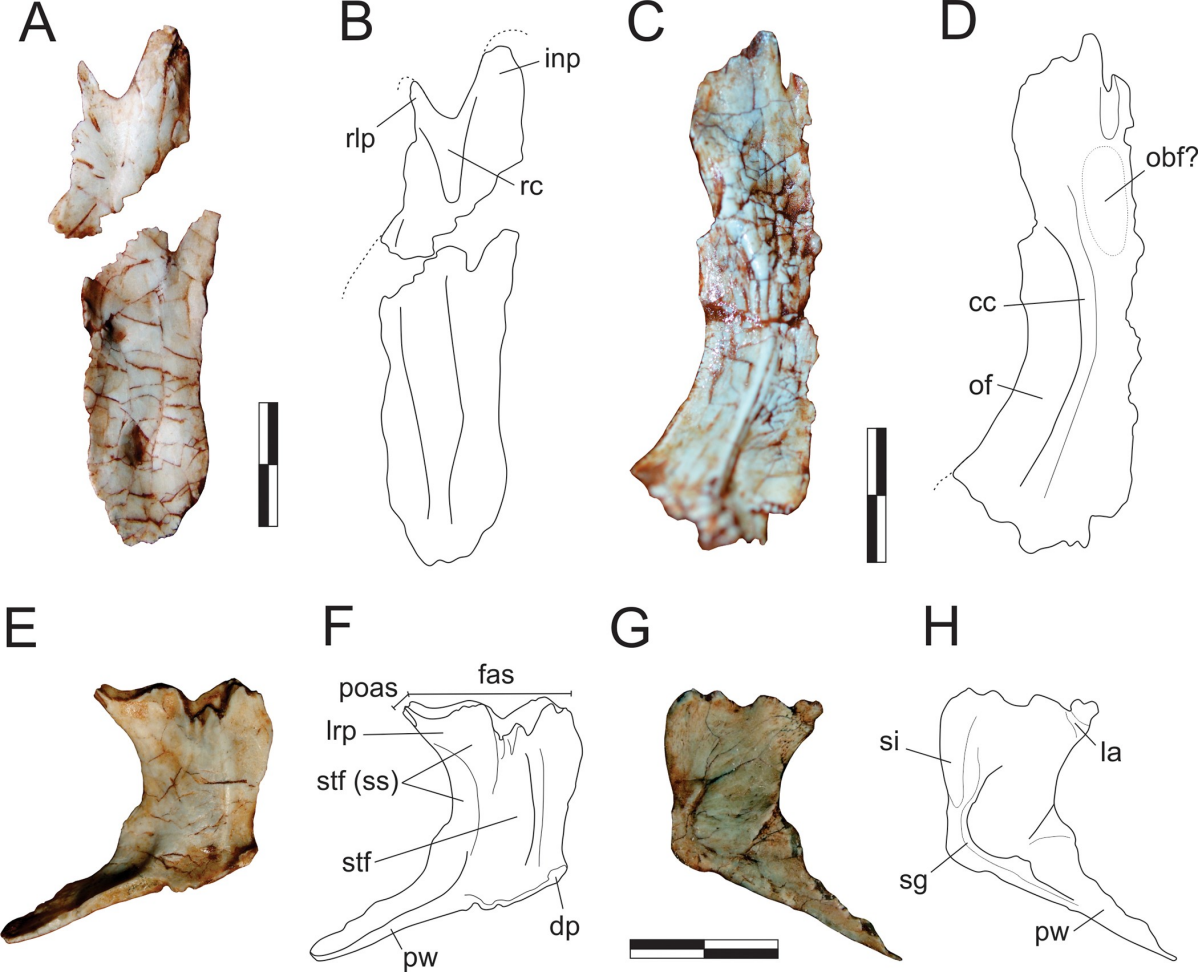
*Pampadromaeus barberenai* (ULBRA-PVT016), nasal, frontal, and parietal. Photographs (A,C,E,G) and drawings (B,D,F,H) of the left nasal in dorsal view (A-B), right frontal in ventral view (C-D), and left parietal in dorsal (E-F) and ventral (G-H) views. Abbreviations: cc, crista cranii; dp, dorsal protuberance of the parietal; fas, frontal articulation surface in the parietal; inp, nasal internarial process; la, laterosphenoid articulation; lrp, parietal laterorostral process; obf, olfactory bulb fossa; of, orbital fossa; poas, postorbital articulation surface in the parietal; pw, parietal wing; rc, nasal rostral concavity; rlp, nasal rostrolateral processes; sg, parietal sinuous groove; si, parietal sinus; stf, supratemporal fossa; stf (ss), supratemporal fossa (*sensu stricto*). Dashed lines indicate inferred skeletal margins. Scale bar = 1 cm.

The left nasal is preserved isolated in the block and exposed in dorsal view. It is 42 mm long as preserved, extending for less than half the estimated skull length [14]. Except for its rostrolateral portion, which forms the caudal margin of the external naris, the remaining outer margins of the bone are incomplete, so that its general shape is elusive. The midline suture is nearly straight, although slightly deformed by a fracture that spans the lateromedial width of the bone. As its lateral margin is missing, it is not clear if the caudal half of the bone was laterally expanded as in *E*. *lunensis* [34] and *Pan*. *protos* [30]–although this is hinted by a small inflection (reconstructed in Fig 6B) of the caudolateral border that potentially represents the rostral beginnings of the expansion–nor if it possessed a caudolateral process overlapping the lacrimal as in most early saurischians [30, 34–35, 63]. Nonetheless, considering the shape of the rostral ramus of the lacrimal (see below), it is most likely that the nasal contacted the dorsal margin of the antorbital fossa. Although the dorsal surface of the bone lacks the deep rostral fossa seen in *Pan*. *protos* [30], a slight elongate concavity (“rc” in Fig 6B) is observable in the corresponding area, as well as transversally across the entire caudal portion of the bone. Both the internarial and the rostrolateral processes are incomplete. The latter extends ventrally from the rostrolateral corner of the bone to cover the maxilla and most probably did not reach the premaxilla.

#### Frontal (Fig 6C and 6D)

The isolated right frontal is preserved in the block and exposed in ventral view. It is an elongate bone, five times longer than transversally broad at mid-length (more than twice longer than the maximal caudal breadth) and subequal in length to the nasal (as preserved). As the caudal margin is not well preserved, its participation in the upper temporal fenestra and articulations with the postorbital and parietal cannot be clearly defined. Nonetheless, the caudal outline of the bone appears to have been rounded, with a slightly more caudally expanded lateral portion and a midline inflection that may have accommodated the rostral tip of the parietal. Although more complete, the rostral and medial margins of the frontal have ambiguous outlines. The medial portion of the rostral margin appears rostrally expanded relative to the lateral portion, possibly tapering to a point along the midline suture. In dorsal/ventral views, the medial margin of the bone is virtually straight and the orbital margin strongly concave. Hence, the rostral and caudal portions of the bone are transversally expanded, the latter much more conspicuously. In ventral view, the transversally concave and lateroventrally facing orbital fossa is separated from the medial portion of the bone by a single, medially arching crista cranii [33]. The fossa occupies half the lateromedial breadth of the frontal at the narrowest section of the bone, but increases in transverse breadth caudally. It is caudally displaced relative to the rostrocaudal midpoint of the bone, extending along its caudal two-thirds and not reaching its rostral margin. The olfactory bulb fossa [33] is not clear, but a subtle concavity is seen mediorostral to the orbital fossa. In the medial portion of the rostral margin, a clear cleft likely received a projection from the nasal. Lateral to that, the ventral surface of the frontal is smooth, and the cavum nasi ridges and depression [33] cannot be clearly recognized. Caudal to the point of maximum expansion of the orbital fossa, the surface medial to the crista cranii forms the transversely concave roofing of the telencephalon.

#### Parietal (Fig 6E–6H)

The left parietal is preserved displaced towards the front of the semi-articulated bones from the right side of the skull, close to the premaxilla. Its ventral surface is exposed in the lateral aspect of the other skull bones, and its dorsal surface in the medial aspect. The bone is composed of a main body and the parietal wing, which expands laterocaudally at an angle of about 110–120° relative to the sagittal line (in dorsal/ventral view) and slightly dorsally. The parietal body is dorsoventrally flattened, whereas the wing expands along the vertical plane, forming a rostrocaudally compressed wall. The dorsal surface of the parietal bears a well-defined longitudinal and medially arching ridge which forms the medial boundary of the supratemporal fossa. Medial to this ridge the bone surface is slightly lateromedially concave, whereas the supratemporal fossa slopes gently ventrolaterally. The aforementioned median ridge intercepts the medial corner of a large inflection that excavates the rostral margin of the parietal. This inflection is probably part of the fronto-parietal suture, and thus the frontal would have been excavated by the supratemporal fossa. Lateral to this inflection, the rostral margin of the parietal projects laterorostrally and slightly dorsally to form the laterorostral process. The articulation of this process is rostrally oriented, probably meeting the frontal inside the supratemporal fossa. In the laterorostral process, but particularly laterocaudal to that, the fossa is deeper and more abruptly sloping, extending towards the dorsoventrally concave rostral surface of the parietal wing. This could be interpreted as the supratemporal fossa *sensu stricto*, the caudalmost portion of which, within the parietal body, is medially bordered by a ridge that represents a (mediorostral) continuation of the dorsal surface of the parietal wing. The laterorostral process bears a tapering laterorostral projection with a reduced articulation area facing that same direction, which potentially reached the postorbital inside the supratemporal fossa. The parietal contribution of the internal supratemporal fenestra is entirely concave laterally, except for a slight lateral swelling at its mediocaudal corner. This represents a lateral expansion of the ventral surface of the parietal, wherein it articulated to the braincase. The mediocaudal corner of the parietal bears a small dorsally raised protuberance (“dp” in Fig 6F), which probably represents an attachment point for the supraoccipital. The parietal wing is subequal in length to the midline interparietal articulation. Its ventral margin is slightly more rostrally positioned than the dorsal, so that the bone wall that forms the caudal margin of the supratemporal fossa is not strictly vertical, but rostroventrally to caudodorsally oriented. No further detail of the parietal wing anatomy is available in the currently exposed ventral view of the bone. The ventral surface of the parietal resembles that described for *Pan*. *protos* [33]. A slightly depressed and rostrocaudally elongated area (= sinus) marks the rostral half of the area adjacent to the interparietal articulation. This is laterally bordered by a subtle ridge that is continuous with another subtle ridge that marks the caudal half of the interparietal articulation and the caudal margin of the parietal wing. These ridges are bordered laterally by a sinuous groove, which is somewhat continuous to a short groove that perpendicularly reaches the margin of the upper temporal fenestra. As in *Pan*. *protos*, the rostrolateral corner of the bone is dorsally projected and bordered medially by a rugosity that may correspond to the laterosphenoid articulation [33].

#### Lacrimal (Figs 1 and 7)

**Fig 7:**
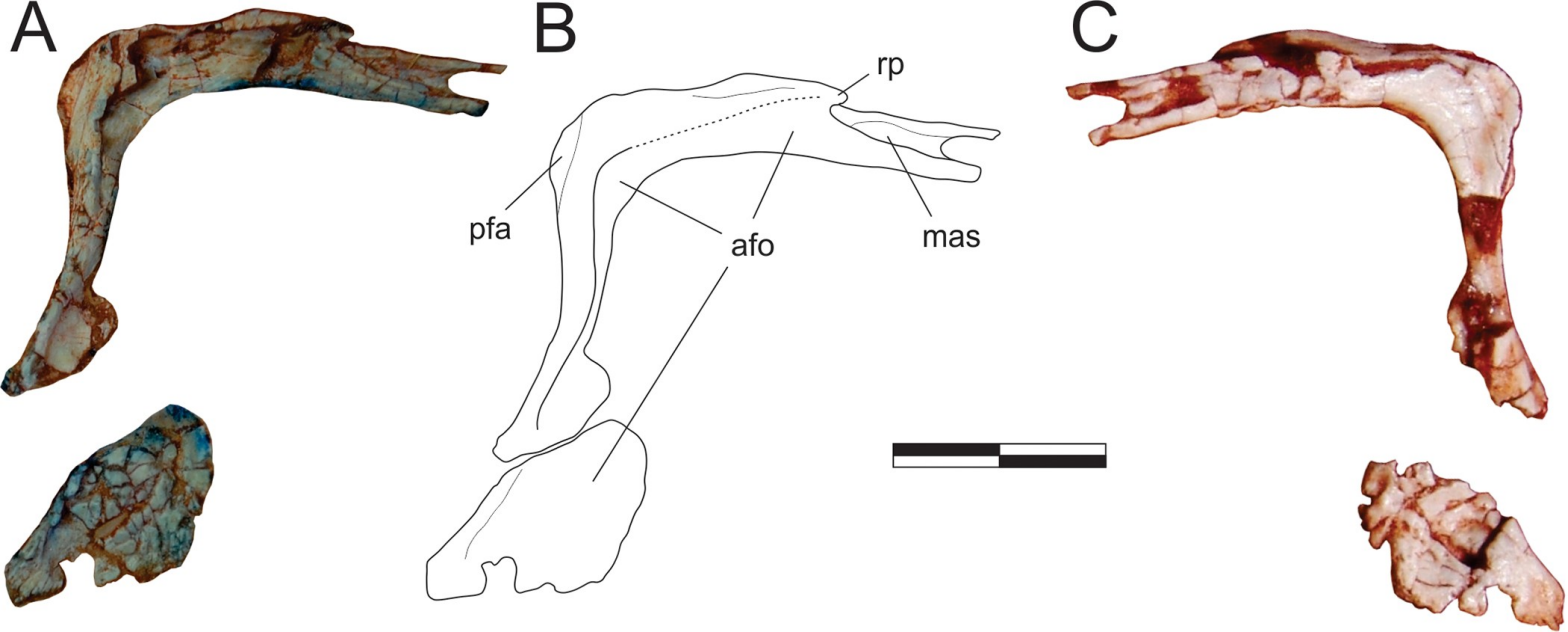
*Pampadromaeus barberenai* (ULBRA-PVT016), lacrimal. Photographs (A,C) and reconstructed drawing (B) of the right lacrimal in lateral (A-B) and medial (C) views. Abbreviations: afo, antorbital fossa; mas, maxillary articulation facet; pfa, prefrontal articulation; rp, rostral process. Dashed lines indicate inferred skeletal margins. Scale bar = 1 cm.

The right lacrimal is preserved semi-articulated to the right maxilla, as part of a skull piece that also includes the right premaxilla, the left palatine, and an undetermined palatal bone. It has an “inverted-L” shape, with the rostral ramus corresponding (as preserved) to nearly 80% the length of the ventral ramus. In lateral view, the dorsocaudal corner of the bone bears a depressed area with a straight, oblique (ventrocaudally to dorsorostrally oriented) rostral margin. This corresponds to the articulation surface for the prefrontal, but the lacrimal apparently lacks a rugose ridge extending from that bone, as seen in *E lunensis* [34]. Although the facial portion of the rostral ramus has a poorly preserved lateral margin, it clearly did not expand ventrally beyond the dorsal margin of the internal antorbital fenestra. As a result, a small strip of the ventral surface of the medial wall of the antorbital fossa is exposed in lateral view, as is also observed in *E*. *lunensis* [34]. The facial portion of the rostral ramus ends rostrally in a small nub of bone (“rp” in Fig 7B) that fits between the caudolateral process of the nasal (not preserved; see above) and the main body of that bone. This corresponds to the caudal, dorsally raised portion of “p2” of Chapelle & Choiniere [64]. Rostral to that, the lacrimal has no facial expression, extending further rostrally onto the antorbital fossa and below the nasal. The lateral surface of that area bears a lateral depression that receives the caudal tip of the dorsal ramus of the maxilla, similar to what is seen in *E*. *lunensis* [34]. The ventral and dorsal borders of that depression form laterally raised and rostrally extensive prongs, the ventral of which fits into a groove on the medial surface of the maxilla. From that area, the antorbital fossa extents caudally along the entire rostral ramus of the lacrimal, but its dorsal margin is mostly broken. The fossa is also exposed laterally at its dorsocaudal corner. As such, although incompletely preserved, it is clear that the dorsocaudal margin of the external antorbital fenestra does not overhang as to laterally hide the medial wall of the fossa. It is, nonetheless, slightly everted as in *E*. *lunensis* [34], with the fossa invaginating dorsocaudally. A short rostral expansion of the facial surface of the lacrimal is seen slightly ventral to that corner. In *E*. *lunensis* [34] and *B*. *schultzi* [35], this expansion is more dorsally positioned, so as to contribute to the lateral covering of the dorsocaudal corner of the antorbital fossa. In *Pam*. *barberenai*, the expansion divides the antorbital fossa into dorsal and ventral portions. In lateral view, the former tappers ventrally from its dorsal summit, whereas the latter expands ventrally along with the entire ventral portion of the bone. This forms the ventrocaudal corner of the fossa, whereby the facial surface represents only about 10% of the rostrocaudal width of the bone at its ventralmost portion. Yet, the ventral end of the lacrimal is very poorly preserved, precluding assessment of its relationship with the maxilla and jugal. The medial surface of the lacrimal is nearly flat, with subtle depressed areas around the dorsocaudal corner of the internal antorbital fenestra and also mirroring the impression of the antorbital fossa onto the medial surface of the ventral ramus. The latter could, however, be a result of taphonomic bone collapse.

#### Prefrontal (Fig 8A and 8B)

The right prefrontal is preserved isolated in the block and is exposed in lateral and dorsal views. It forms the dorsorostral margin of the orbit and is composed of caudal, ventral, and rostral rami, the latter of which is the shortest and less robust of the three. A rugose ridge extends along the lateral surface of the caudal ramus, separating the skull roof from the orbital fossa. Rostrally, it reaches the juncture of the rostral and ventral rami, which diverge from one-another at an angle of 60° (in lateral view). At this point, the prefrontal was laterally covered by the lacrimal, and the ridge possibly contacted that bone. Indeed, as in all known Carnian forms [30, 34–35], the prefrontal of *Pam*. *barberenai* lacks the sheet of bone covering the lacrimal, which is present in various sauropodomorphs [65]. The rostral process bends slight dorsally and medially at its rostral end. The ventral ramus is curved; concave caudally and convex rostrally. Ventrally, a longitudinal slot occupies more than half of the rostral surface of the ventral ramus, wherein it articulated with part of the lacrimal. Except for its rostral-most margin, the entire lateral surface of the ventral ramus is occupied by the orbital fossa.

**Fig 8:**
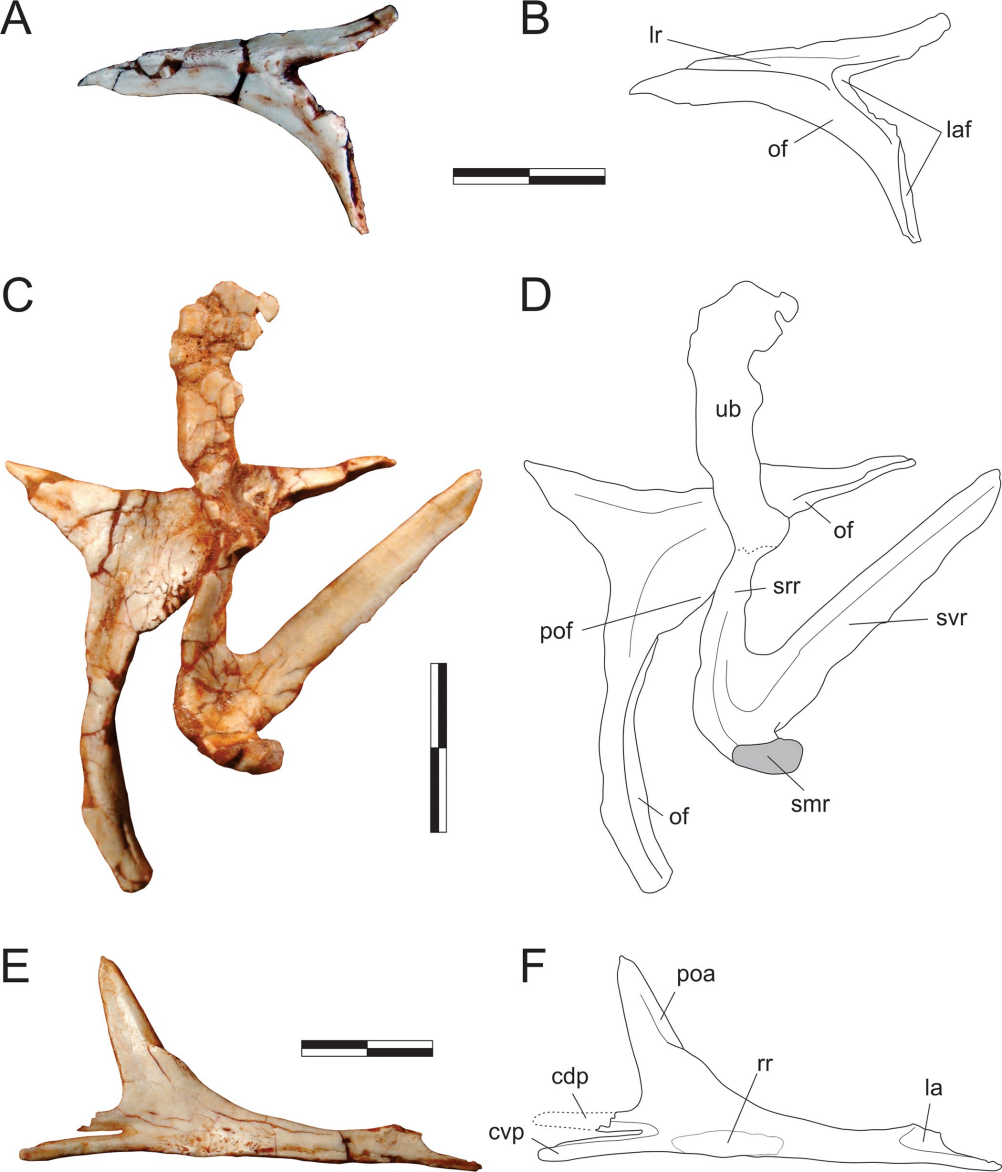
*Pampadromaeus barberenai* (ULBRA-PVT016), prefrontal, postorbital, jugal, and squamosal. Photographs (A,C,E) and drawings (B,D,F) of the right prefrontal in lateral view (A-B), right postorbital in lateral and left squamosal in medial views (C-D), and left jugal in medial view (E-F). Abbreviations: cdp, caudodorsal prong; cvp, caudoventral prong; la, lacrimal articulation area; laf, lacrimal articulation facets; lr, lateral ridge; of, orbital fossa; poa, postorbital articulation; pof, postorbital flange; rr, jugal rugose ridge; smr, squamosal medial ramus; srr, squamosal rostral ramus; svr, squamosal ventral ramus; ub, undetermined bone. Dashed lines indicate inferred skeletal margins; grey areas indicate broken surfaces. Scale bar = 1 cm.

#### Postorbital (Fig 8C and 8D)

The right postorbital is preserved together with the left squamosal and an undetermined bone fragment, disarticulated on the block and exposed in lateral view. It is a triradiate element, with a main central body and three (caudal, rostral, and ventral) rami. The ventral ramus is the longest, followed by the rostral ramus. A flange extends ventrorostraly from the juncture of the rostral and ventral rami, slightly overlapping the dorsocaudal margin of the orbit. This represents a thickening of the orbital border, and continues as the ventral margin of the rostral ramus and the rostral margin of the ventral ramus. The rest of the bone is less lateromedially expanded, including the dorsocaudal portion of the body and the more laminar caudal ramus. Whereas the ventral ramus is formed entirely of the thickened orbital border, the base of the rostral ramus has a small laminar dorsal portion. The relationship of the postorbital to the surrounding skull bones is not clear. A subtle rostral inflection is observed at the ventral end of the ventral ramus, but not to the extent seen in *E*. *lunensis* [34]. This suggests that this part of the bone rostrally overlapped the dorsal ramus of the jugal, although no articulation facet is visible.

#### Squamosal (Fig 8C and 8D)

The left squamosal is preserved together with the right postorbital and an undetermined bone fragment, disarticulated within the block and exposed in medial view. It preserves the rostral and ventral rami, as well as the base of the caudal and medial rami. The ventral ramus is twice the length of the rostral ramus. Both of them are laminar (lateromedially compressed), and diverge at a 45° angle from one another. The rostral ramus curves ventrally. Its lateral surface is not exposed, and this renders uncertain if it bore a slot for articulation with the postorbital. The ventral ramus is straight and elongate, more than five times longer than the maximum breadth of its base. Although its lateral surface is not exposed, the ramus is more laminar towards its caudal margin, suggesting that this portion was laterally overlapped by the quadrate. The dorsally arching base of the medial ramus indicates that it was not laminate, but more rod-like. It forms an angle of 130°, 90°, and slightly over 90° to the ventral, caudal, and rostral rami, respectively. If not deformed, it is probable that the medial ramus was more caudally than rostrally oriented. The upturned base of the caudal ramus (which is hidden lateral to the medial ramus in medial view; Fig 8C and 8D) shows that it was lateromedially compressed, likely covering the quadrate head dorsally and overlapping the parietal wing laterally. It is laterally continuous with the rostral ramus, forming an angle of about 140°; the angle with the ventral ramus is slightly over 90°.

#### Jugal (Fig 8E and 8F)

The left jugal is preserved isolated in the block and exposed in medial view, not lateral as described by Cabreira *et al*. [14]. Accordingly, features of the lateral surface of that bone are inaccessible. The jugal is typically triradiate, with rostral, dorsal, and caudal rami. Its ventral margin forms a nearly straight line, with no offset between the rostral and caudal rami. The rostral tip of the rostral ramus has an upturned dorsal margin, but is incomplete rostral to that. This upturned margin is excavated by a medial depression that likely marks the contact area for the lacrimal, where the jugal was probably medially covered by that bone. The ventral margin of the caudal end of the rostral ramus bears a rugose longitudinal ridge. This is covered by subtle pits and radiating grooves, and probably received the ectopterygoid. The ridge gets less rugose as it extends rostrally until the mid-length of the rostral ramus, whereupon it forms a sharp lateroventral margin. A subtle concave surface extends along the caudal part of the dorsal ramus and dorsal part of the caudal ramus. The dorsal ramus forms an angle of 120° to the rostral ramus and bears a slot along its rostral margin for reception of the ventral ramus of the postorbital, with the latter laterally covering the former. The caudal termination of the caudal ramus is bifid, forking into subequal dorsal and ventral prongs to meet the rostral ramus of the quadratojugal. Although the dorsal prong was lost during preparation, its length can be reconstructed based on the impression left in the matrix. The fossa that borders the slot in medial view receives the rostral ramus of the quadratojugal, with only the central part of this ramus exposed in lateral view: its dorsal margin being laterally covered by the dorsal prong and its ventral margin covered laterally by the ventral prong. A similar arrangement is described for *E*. *lunensis* [34].

#### Quadrate (Fig 9A and 9B)

The left quadrate is preserved isolated in the block and exposed in caudal view. The vertical plane through the condyles is aligned perpendicular to the pterygoid flange and subparallel to the lateral flange. The medial condyle is more ventrally expanded than the lateral, so that the ventral margin across the condyles is oblique. They are set apart by a well-developed rostrocaudally oriented groove. The quadrate body bows rostrally as it expands dorsolaterally from the medial condyle, forming a pillar that separates the lateral and pterygoid flanges and dorsally forms the quadrate head. Both flanges are depressed adjacent to the vertical pillar. The pterygoid flange has a typically crescentic dorsomedial margin, extending for almost 80% of the total length of the bone. It does not reach as far ventrally as the lateral flange, forming a waisted ventral margin. The lateral flange is slightly bent rostrally. Its outer margins are incomplete in the area of the quadrate foramen. This area is broken and depressed, suggesting that the foramen is mostly enclosed in the quadrate, but clear vestiges of the foramen are missing. The tip of the quadrate head is missing.

**Fig 9:**
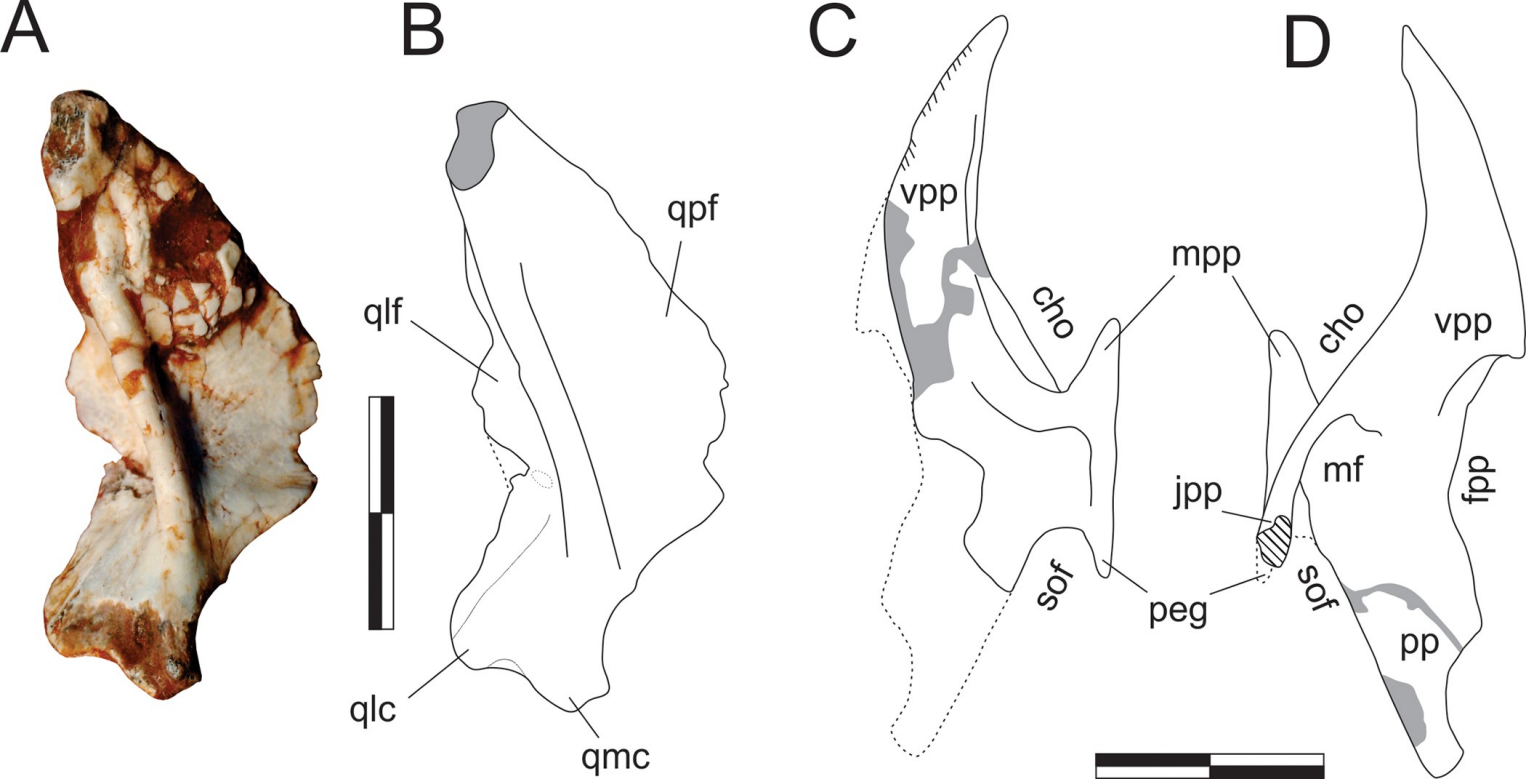
*Pampadromaeus barberenai* (ULBRA-PVT016), quadrate and palatine. Photograph (A) and drawings (B-D) of the left quadrate in caudal view (A-B) and left palatine in medial (C) and lateral (D) views. Abbreviations: cho, choanal aperture; fpp, fenestra pterygopalatine; jpp, jugal process of the palatine; mf, muscular fossa; mpp, maxillary process of the palatine; peg, palatine peg; pp, pterygoid process; qlc, lateral condyle of the quadrate; qlf, lateral flange of the quadrate; qmc, medial condyle of the quadrate; qpf, pterygoid flange of the quadrate; sof, suborbital fenestra; vpp, vomeropterygoid process. Dashed lines indicate inferred skeletal margins; hatched/grey areas indicate broken surfaces. Scale bar = 1 cm.

#### Palatine (Figs 1, 9C and 9D)

The left palatine is preserved medial to the right maxilla, semi-articulated with the skull piece that also includes the right premaxilla and lacrimal. Its medial surface is visible when those bones are viewed in lateral aspect (Figs 1A and 9C), its lateral surface in the obverse (Figs 1B and 9D). The bone is mostly complete, but displaced rostrally and ventrally from its original position in the palate. The flattened, elongate, and rostrally tapering element observed rostrodorsally to the palatine may represent the rostral tip of either the pterygoids or potentially (although less likely) part of the vomeri. Its shape approximates that of the rostral part of the left palatine, but it lacks the striated medial surface of the dorsal margin seen in that element. It bears instead a thickened margin facing the palatine and is not here regarded as its counterpart. The palatine is a lateromedially flattened, anvil-shaped element with dorsomedial and ventrolateral bodies separated by a rostrocaudally constricted area that separates the choanal aperture from the suborbital (= postpalatine [34]) fenestra [66]. The ventrolateral body expands into rostral and caudal portions, the straight lateral margin of which serves for the articulation with the maxilla and, probably, the jugal. The short rostral prong (= maxillary process [67]) is completely preserved, but the caudal part of the ventrolateral body is partially covered by matrix and difficult to interpret. As preserved, it is composed of a short and slightly laterally expanding jugal process [67]. The entire length of the jugal process, as well that of a second medial prong (“peg” of Prieto-Márquez & Norell [65]) cannot be confirmed. On the lateral surface, the jugal process is continuous with a ridge that extends rostrally forming the lateroventral margin of the caudal portion of the vomeropterygoid process [67]. This crest rises laterally relative to the depressed muscular fossa, caudodorsally [68], and the maxillary process, rostroventraly. The dorsomedial body of the palatine corresponds to a rostrocaudaly extensive and lateromedially flattened structure. Its rostral portion represents to the vomeropterygoid process [67], whereas the caudal part corresponds to the pterygoid process of Eddy & Clarke [69] (= “posterior process” of Prieto-Márquez & Norell [65]), which is entirely occupied by the muscular fossa. These two elements are separated by a concave dorsal margin, which correspond to the fenestra pterygopalatina [66]. The vomeropterygoid process has a medially striated dorsal edge, which corresponds to the midline articulation of the palatines. The rest of the medial surface of the bone is mostly flat, but bears a slightly elevated ribbon-like platform, which encompasses the rostral prong of the ventrolateral body, extending dorsomedially to loop towards the vomeropterygoid process. Shallow fossae are visible rostrally and caudally to the platform, respectively occupying the caudal part of the ventral margin of the vomeropterygoid process and the central part of the platform constricted portion of the palatine.

#### Pterygoid (Fig 10)

**Fig 10:**
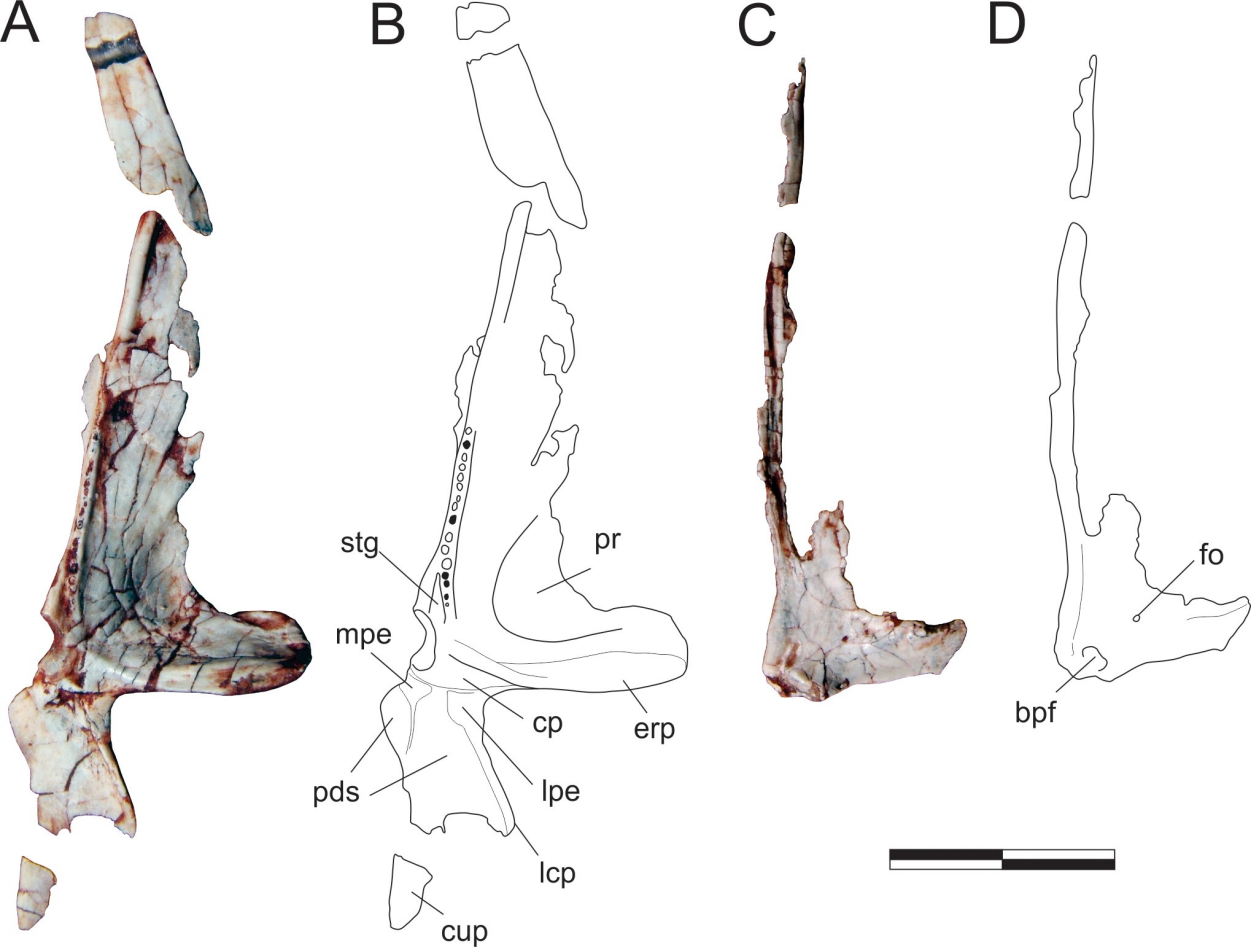
*Pampadromaeus barberenai* (ULBRA-PVT016), pterygoid. Photographs (A,C) and drawings (B-D) of the left pterygoid in ventral (A-B) and right pterygoid in dorsal (C-D) views. Abbreviations: bpf, basipterygoid flange; cp, central platform; cup, caudal process; erp, ectopterygoid ramus of the pterygoid; fo, foramen; lcp, laterocaudal process; lpe, lateral protruding eminence; mpe, medial protruding eminence; pds, pneumatic depressions; pr, pneumatic recess; stg, subtriangular groove. Scale bar = 2 cm.

Right and left pterygoids are preserved isolated in the main block, the right exposed in dorsal view and the left in ventral view. The more completely preserved left element shows that the pterygoid is a triradiate bone, with a long rostral (= vomeropalatine) ramus, and shorter lateral (= ectopterygoid, mandibular) and caudal (= quadrate) rami. As preserved in the left element, the rostral ramus corresponds to more than 60% of the entire length of the pterygoid, although it must be missing its rostral tip. Although generally laminar (dorsoventrally flattened) in structure, the rostral ramus bears a thickened medial margin along its caudal two thirds. This margin manifests as a mediolaterally thin and ventrally raised platform that is marked by a row of fifteen tiny dental alveoli within its caudal half, five of which (painted black in Fig 10B) preserve teeth. The caudal-most part of this platform deflects laterally, diverging from the ventrally raised medial margin of the bone and creating a shallow a subtriangular groove (“stg” in Fig 10B) between them. Immediately caudal to this divergence the medial margin of the pterygoid forms a small but pronounced concavity, also seen in various other early dinosaurs [45, 70]. This forms the lateromedially expanded caudal portion of the interpterygoid vacuity, presumably below the base of the parabasisphenoid rostrum in the articulated palate. The lateral ramus of the pterygoid expands laterally, forming a right angle to the rostral ramus. The caudal margin of that ramus is braced by a thickened, ventrally raised ridge that extends along its entire preserved length, shallowing out directly lateral to the abovementioned medial concavity. Rostral to the thickened caudal margin, the lateral ramus is ventrally excavated, forming a dorsoventrally flattened lamina that is continuous with the lamina that comprises the main body of the rostral ramus. The confluence of those two laminae is strongly depressed at its centre, with the whole area bordered by the thickened margins representing a pneumatic recess [66]. The lateral margin of the lateral ramus is poorly preserved, thus it is not clear how much of the flange was ventrally covered by the ectopterygoid. Nonetheless, the laterocaudal corner of the flange appears to have formed a rounded articulation similar to that seen in the left pterygoid of *E*. *lunensis* [34]. Indeed, as discussed by Sereno *et al*. [34], the pterygoid of *Pam*. *barberenai* may have abutted the ectopterygoid, not extending caudal to it along the transverse palatal flange. The caudal ramus of the pterygoid is also dorsoventrally flattened and slightly dorsally oriented. It has a triradiated dorsal/ventral outline with an oblique (laterocaudal) lateral process and an elongate caudal process which correspond, respectively, to the ventral and dorsal processes of the quadrate ramus in *Pl*. *erlenbergensis* [65]. A stoutly projecting medial flange produces a convex outline between the rostromedial corner of the caudal ramus and its caudal process. In ventral view, the caudal ramus is separated from the rest of the bone by a transverse groove, immediately caudal to a platform (“cp” in Fig 10B). That groove is continuous with a short groove extending caudally. This separates two protruding eminences (“mpe” and “lpe” in Fig 10B) and is confluent with the excavated surface that occupies most of the ventral surface of the ramus. The lateral eminence is continuous to the thickened lateral margin of the oblique lateral process, whereas the medial eminence extends caudally as subtle ridge. This separates the ventral surface of the caudal ramus of the pterygoid into two (small rostromedial and large caudolateral) shallow depressions of possible pneumatic origin. The right pterygoid has a preserved main body (i.e. the confluence of the three rami), a good deal of the lateral ramus and parts of the rostral ramus. This includes its caudalmost portion, as well as most of its thickened medial margin, which extends until the rostral tip of the ramus as preserved. Well preserved specimens of other Carnian sauropodomorphs (e.g. *E*. *lunensis* [34]; *B*. *schultzi* [35]) lack such a complex caudal ramus of the pterygoid. Yet, this is a rather fragile element, which could have been easily lost by over-preparation. The right pterygoid is exposed in dorsal view and bear a small foramen at the base of the lateral ramus. No equivalently positioned ventral aperture is visible in the left bone, which would be considerably caudal to the foremen reported for *E*. *lunensis* [34]. The dorsal surface of the lateral ramus slopes ventrally within the caudal half, indicating that the ramus is rostrodorsally to caudoventrally oriented, a condition that is congruent with the left element. As preserved, the mediocaudal corner of the right pterygoid bears a protruding knob, which probably represents an underdeveloped version of the basipterygoid flange of other sauropodomorphs [65]. It is separated from the thickened medial margin of the bone by a groove and caudally continuous with a short and dorsally displaced platform. This whole structure represents the dorsal and caudomedially facing articulation for the basipterygoid process of the parabasisphenoid, in a position that corresponds (in the ventral surface of the right bone) to that of the medial concavity, at the caudal margin of the rostral ramus.

### Lower jaw

Lower jaw bones are mostly preserved scattered on the block, except for the semi-articulated right angular, surangular, articular, and prearticular. Vestiges of the coronoid and splenial have not been recognised in the assemblage.

#### Dentary (Figs 11, 12A and 12B)

**Fig 11:**
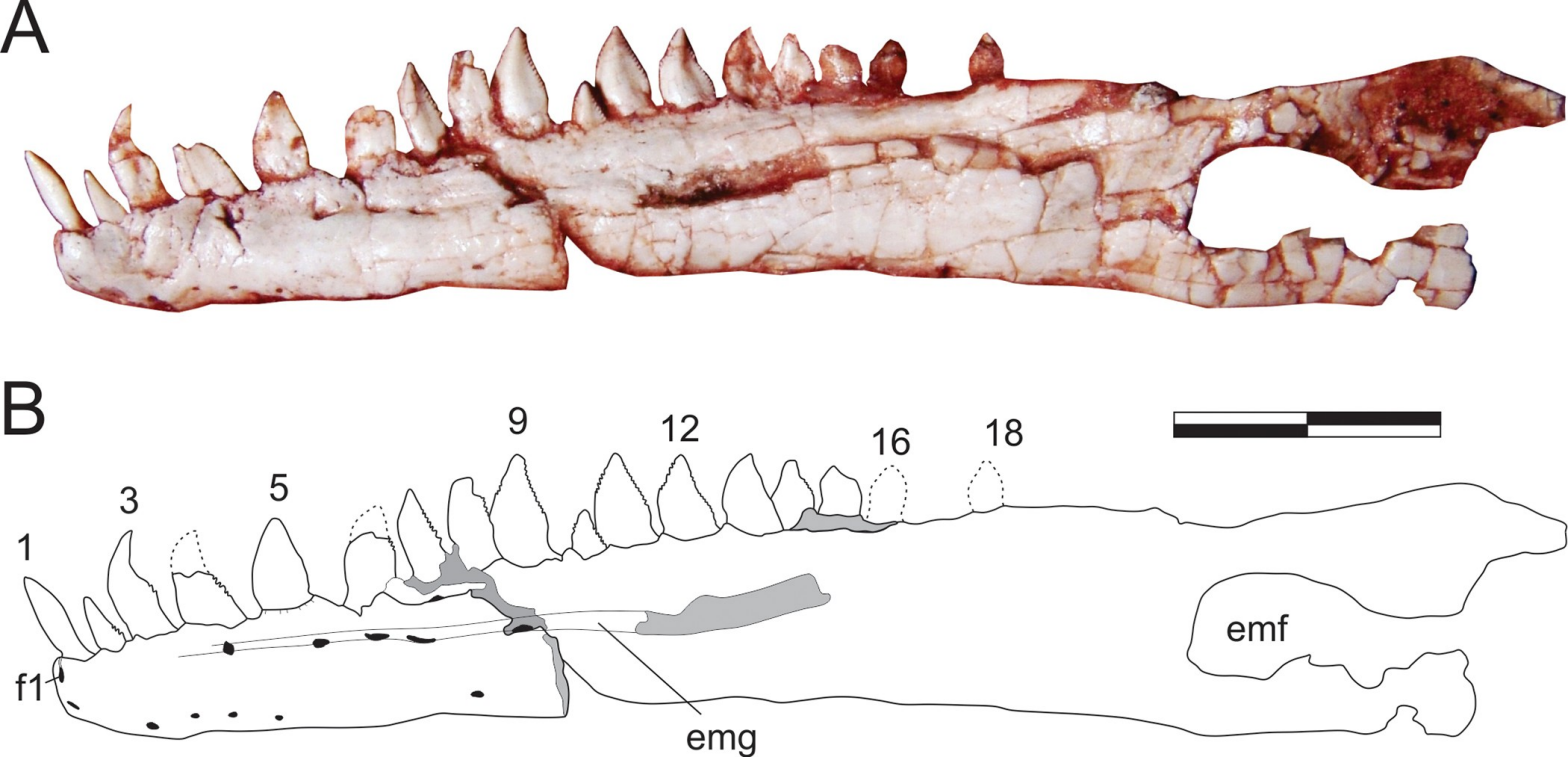
*Pampadromaeus barberenai* (ULBRA-PVT016), dentary. Photograph (A) and drawing (B) of the left dentary in lateral view. Abbreviations: 1–18, tooth positions; emf, external mandibular fenestra; emg, external mandibular groove; f1, foramen “1”. Dashed lines indicate inferred skeletal margins; grey areas indicate broken surfaces; foramina in black. Scale bar = 1 cm.

**Fig 12:**
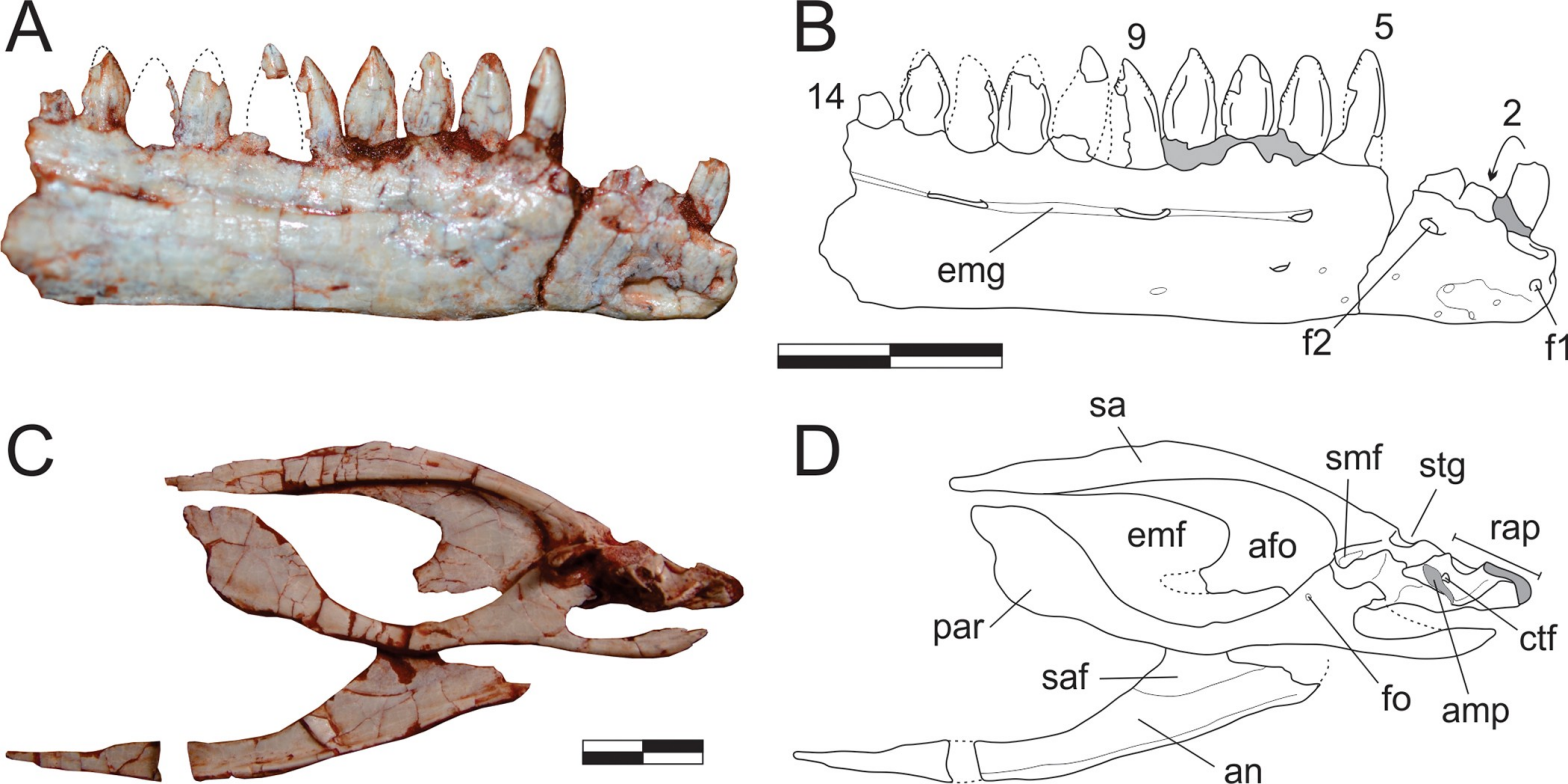
*Pampadromaeus barberenai* (ULBRA-PVT016), lower jaw. Photographs (A,C) and drawings (B,D) of the right dentary in lateral (A-B) and right postdentary lower jaw bones in medial (C-D) views. Abbreviations: 2–14, tooth positions; afo, adductor fossa; amp, articular medial process; an, angular; emf, external mandibular fenestra; emg, external mandibular groove; f1, foramen “1”; f2, foramen “2”; ctf, chorda tympani foramen; fo, foramen; par, prearticular; rap, retroarticular process; sa, surangular; saf, articulation facet for surangular; smf, surangular medial flange; stg, surangular transverse groove. Dashed lines indicate inferred skeletal margins; grey areas indicate broken surfaces; arrow connects broken parts of the second tooth. Scale bar = 1 cm.

The nearly completely left dentary is preserved isolated in the main block (Fig 11), possibly with the rostral portion of the angular and surangular adhered to it, and exposed in lateral view. Isolated on a smaller block, a rostral portion of the right dentary is also preserved in lateral view (Fig 12A and 12B). The twelve rostral-most teeth of left dentary are well preserved. Contra to what has been suggested for various other sauropodomorphs [34], the first mandibular tooth is not inset from the rostral tip of the dentary. The remaining dentary teeth are dorsally crushed by a phalanx. Nonetheless, five smaller teeth, as well as an empty tooth position, are clearly seen, indicating that *Pam*. *barberenai* had a minimum of 18 dentary teeth. In addition, the caudal-most region of the tooth row indicates space on the dentary that would potentially allow for two extra teeth. The caudal portion of the right dentary as preserved bears a sequence of ten alveoli, with seven well preserved and two fragmentary crowns. The rostral-most tip of this dentary is more poorly preserved and separated from the rest of the bone by a transverse fracture. It potentially accommodated an additional 3–4 teeth, but only the first empty alveolus is well preserved. The crown fragment positioned dorsal to the empty alveolus is likely related to the crown base preserved *in situ* in the preceding tooth (Fig 12B). That empty alveolus is ovoid (rostrocaudaly elongated) in outline and is separated from the rostral margin of the jaw by only the lip/border that surrounds the depression, confirming that the first dentary tooth is not caudally inset.

The shape of the right and left dentaries differs slightly in lateral view. The latter gradually papers along the rostral-most two centimetres of the bone, whereas the right dentary has nearly parallel dorsal and ventral margins until about one centimetre form its rostral tip, whereupon it tapers abruptly. This approaches the condition seen in *Pan*. *protos* [30] and *E*. *lunensis* [34], in which the dentary is dorsoventrally expanded near its rostral tip and has a ventrally inclined dorsal margin rostral to that. The caudal half of the bone has a slightly concave ventral surface, as reported for *P*. *protos* [30]. The lateral surface of the right dentary has a clear foramen near the rostral tip of the bone (“f1” in Fig 12B), followed by a series of small foramina along the lateroventral portion of the rostral 2 cm of the bone. Another group of more dorsally positioned foramina forms a row below the tooth-line. The rostral-most of these (“f2” in Fig 12B), is positioned directly below the third tooth position and is similar to the comparatively larger foramen reported for *E*. *lunensis* [34]. The following foramina are positioned on a groove (= external mandibular groove of Martínez & Alcober [30]) that extends below the nine caudal-most preserved tooth positions. The first of these latter foramina is subcircular in outline, whereas the two following intrusions, located below the fourth and seventh preserved teeth, are more elliptical in outline. The external mandibular groove is deepest caudal to the last of these foramina, as reported for *P*. *protos* [30]. The same groove is not as marked in the left dentary, but extends from caudal to the third tooth at least until a breakage on the lateral surface of the dentary below teeth 11–13, so that its continuation onto the suragular cannot be confirmed. It hosts five clear foramina, one below the fourth tooth, three forming a cluster below teeth 5–7, and one below teeth 8–9. As in the right bone, a large foramen is seen at the rostral margin of the bone, also followed by a series of small foramina on the lateroventral edge of the dentary. These were termed anterior dentary foramina in *E*. *lunensis* [34], and suggested as tentative evidence for the presence of a keratinous beak. However, in contrast to *E*. *lunensis*, these are not located on the lateral surface of the dentary tip in *Pam*. *barberenai*. It is unclear to what degree the large opening on the lateral surface of the caudal portion of the left dentary corresponds to the external mandibular fenestra. With the possible exception of its rostroventral corner, most of its margin is broken. In any case, although an external mandibular fenestra is most probably present, its size and shape cannot be safely reconstructed. Likewise, the caudal contact of the dentary to the other mandibular bones cannot be confidently determined.

#### Surangular (Figs 11, 12C, 12D and 13)

**Fig 13:**
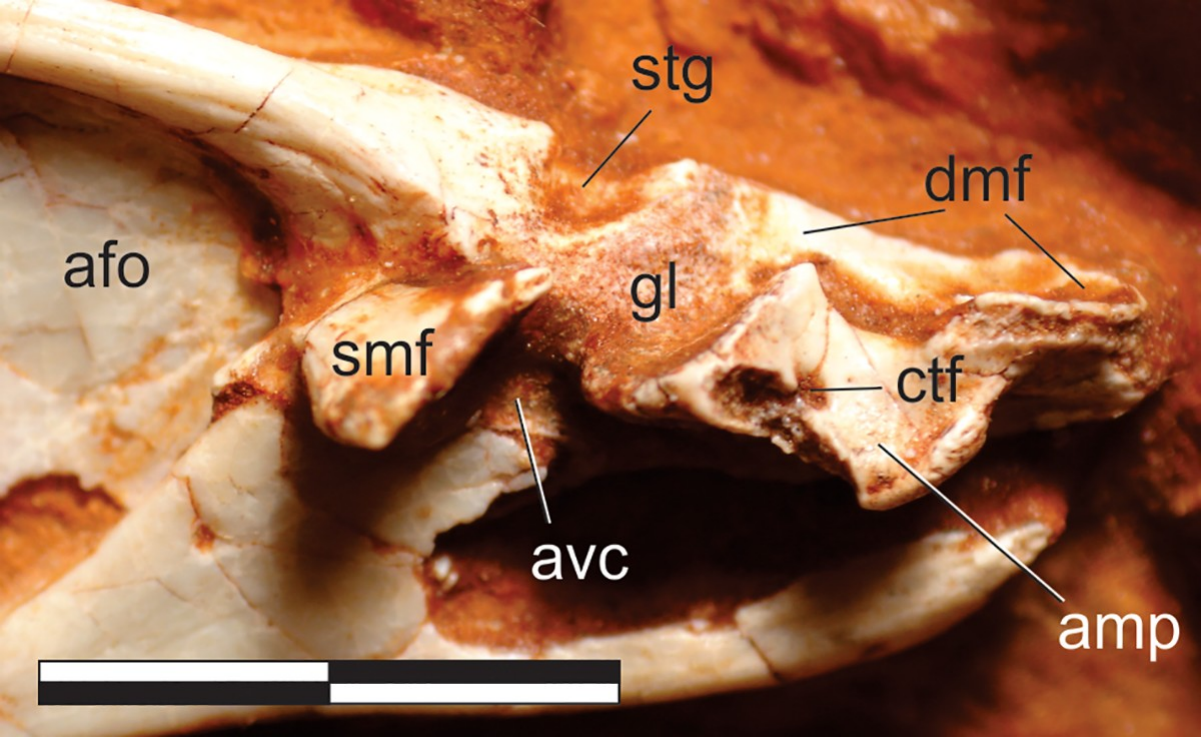
*Pampadromaeus barberenai* (ULBRA-PVT016), lower jaw. Detail of the lower jaw articulation in medial view. Abbreviations: afo, adductor fossa; amp, articular medial process; avc, articular ventral column; ctf, chorda tympani foramen; gl, glenoid; dmf, attachment facet for m. depressor mandibulae; smf, surangular medial flange; stg, surangular transverse groove. Scale bar = 1 cm.

The right surangular is exposed in medial view, articulated in the main block to other postdentary lower-jaw bones (Fig 12C and 12D), whereas the rostral tip of the left bone may be preserved with the respective dentary (Fig 11). The surangular is a rostrocaudally elongated and lateromedially flattened bone. It is comprised of a lateromedially thick and dorsally arched dorsal body, with a bony lamina extending ventrally from the lateral margin of its the caudal portion. This lamina laterally bounds the dorsocaudal portion of the adductor fossa, forms the dorsocaudal and caudal margins of the external mandibular fenestra, and is laterally overlapped by the angular at its ventral margin. The dorsal body frames the adductor fossa dorsally, also forming part of the dorsal margin of the external mandibular fenestra. The surangular gradually expands dorsoventrally towards its rostral end, with its ventral margin meeting the dorsal margin of the prearticular and enclosing the adductor fossa. The surangular-prearticular contact there was probably covered by the coronoid, although that bone is not preserved. The caudal portion of the surangular expands lateromedially to integrate the cranio-mandibular join (Fig 13). In this area, the dorsal body is truncated by a deep transverse groove (“stg” in Fig 13), also seen in *Pan*. *protos* [30], which is caudally and medially bordered by a rough surface. This surface contacts the articular caudally and medially, although the suture is not clearly visible. The medial flange of the surangular [71–72] expands medially as a plate-like bone projection from the medial surface between the transverse groove and the adductor fossa and is ventrorostrally to dorsocaudally oriented in medial view. It delimits the caudal margin of the adductor fossa and dorsally overhangs the prearticular. The rough bone surface, as well as the caudal surface of the medial flange, represents the surangular contribution to the glenoid. The surangular probably also expanded caudally, laterally overlapping the articular to form the lateral margin of the retroarticular process. However, as the lateral surface is not exposed, this cannot be confirmed, which also renders uncertain the presence/shape of the surangular foramina and ridge [30].

#### Angular (Figs 11, 12C and 12D)

The right angular is exposed in medial view, articulated to other postdentary bones of the lower-jaw (Fig 12C and 12D), whereas the rostral tip of the left bone may be preserved with the respective dentary (Fig 11A and 11B). It is a simple, rostrocaudally elongated and lateromedially flattened bone that forms most of the caudal portion of the mandibular ventral margin. As preserved in the right element, the rostral portion of the angular tapers towards the ventral margin of the mandible. It possibly bore a broken-off dorsal process, as seen in *Pan*. *protos* [30] and *E*. *lunensis* [34]. Although both the shape of the rostral portion of the bone and its contact with the dentary and splenial cannot be discerned in either element, it is nonetheless likely that the angular formed the greater part of the ventral margin of the external mandibular fenestra. The ventral margin of the caudal half of the angular expands medially to articulate with the prearticular, forming the floor of the adductor fossa. At this point, a laminar shelf expands dorsally to laterally cover the caudoventral portion of the adductor fossa, receiving the ventral projection of the surangular medially on a depressed articular facet (“saf” in Fig 12D). The caudal end of the angular tapers strongly (in lateral/medial views), but is unclear how far caudally it extended along the ventral margin of the mandible.

#### Prearticular (Figs 12C, 12D and 13)

Both prearticulars are preserved, the left within the main block and exposed in lateral (inner) view, whereas the right is exposed in medial (outer) view along with the other postdentary bones to which it is articulated (Fig 12C and 12D). It is a rostrocaudally elongate and lateromedially flattened bone with a lateral/medial outline composed of dorsoventrally expanded rostral and caudal portions separated by a central constriction, which forms the ventral margin of the internal mandibular fenestra. Its rostral expansion, which contacts the surangular dorsally, forms the rostral margin of that fenestra and lacks the raised lip described for *E*. *lunensis* [34]. In the isolated left element, the caudal expansion has a subtriangular lateral/medial outline, with a deeper rostral portion and tapering caudal end, as in *E*. *lunensis* [34] and *Pan*. *protos* [30], but its margins are not entirely preserved. In the right element, the rostral portion of the caudal expansion is pierced by a foramen, directly caudal to the adductor fossa. Caudal to that foramen, the prearticular is dorsally overhung by the surangular medial flange and its more caudal portion articulates laterally and dorsally with the articular. A strong embayment is present on the dorsocaudal margin of the prearticular, which probably represents a breakage as an equivalent morphology is not seen in its left counterpart. Nonetheless, an inflection at this point on the dorsal margin of the prearticular (also not clear in the left bone of *Pam*. *barberenai*), whereupon it receives the articular, has been described in other sauropodomorphs [30, 35, 65]. At its caudal end, the prearticular forms the lateroventral portion of the retroarticular process.

#### Articular (Figs 12C, 12D and 13)

The right articular is preserved with its medial and dorsal surfaces exposed, articulated to other postdentary bones of the lower-jaw (Fig 13). The articular is composed of a rostrocaudally elongated main body and the medial process [73]; “pyramidal process” of Prieto-Márquez & Norell [65]. The former takes part on a small (caudal) portion of the lateral part of the glenoid, at the contact with the surangular. The caudal part of the main body forms the excavated (for m. depressor mandibulae) lateral portion of the dorsal surface of the retroarticular process, which extends caudally beyond the caudal margin of its medial portion (formed by the medial process). The medial process contributes most of the medial portion of the glenoid, ventral to which the articular bears a columnar extension (“avc” in Fig 13) that meets the prearticular. The rostral margin of the medial process forms the subvertical and slightly concave caudal extension of the glenoid. The part of the medial process caudal to the glenoid is more dorsoventrally flattened and dorsoventrally traversed by the chorda tympani foramen. The medial process has a broken medial margin and a thickened and more rugose caudal margin. As in *Pan*. *protos* [30], the whole glenoid is rostromedial to caudolaterally oriented, ventrally dipping towards the medial side, and not transversely boarder than the rostral portion of the retroarticular process.

### Marginal dentition

The marginal dentition of *Pam*. *barberenai* is typically polyphyodont, with an alternated pattern of tooth replacement [74]. This results in teeth being preserved in different eruption phases, including advanced stages of dental exfoliation. Some alveoli are empty, whereas in the opposite extreme of tooth replacement, some are fused to fully erupted teeth. Such fusion occurs by the ankylotic apposition of attachment bone composed of cementum-like tissues of dental origin [75–76]. This often forms a protruding ring around the base of the crown, extending the alveolar margin apically. The tooth-alveolus fusion in *Pam*. *barberenai* occurs near the cervical constriction, so that most of the cervical margin would take part of the tooth crown, *de facto* exposed in the mouth cavity. This suggests that teeth were retained even in advanced stages of root exfoliation, extending their lifespan, as more commonly seen in herbivorous taxa. Root resorption would be, therefore, typically associated with a well-developed ring of attachment bone in senile teeth.

#### Premaxillary teeth (Fig 2)

The premaxilla of *Pam*. *barberenai* bears four teeth. The labial alveolar margin is preserved only in the second tooth. In all other teeth, the more apical part of the lateral surface of the root is exposed due to erosion of the lateral wall of the alveoli. Only the alveolar margin above the first tooth has been lost in the lingual surface. All teeth are labiolingually compressed, with well-developed mesial and distal carinae. The first tooth is inset c. 1.5 mm from the rostral-most margin of the bone. However, this is likely an artefact of its not being fully erupted at the time of death. Fully grown, it is likely the tooth would have left a much smaller rostral gap. Its crown is “leaf-shaped” in lingual view, i.e. with a faintly convex mesial margin and a sigmoid (convex at the base and concave apically) distal margin. As common to early sauropodomorphs [5, 34], the crown of the first premaxillary tooth is somewhat straighter and less mesiodistally expanded than more caudal tooth crowns. It also lacks a central convexity on its lingual surface, which is more developed and longitudinally expansive in the third and fourth premaxillary teeth. Crowns of both the first and second teeth lack denticles or serrations altogether. The crown of the second tooth, best observed in lingual aspect, is also “leaf-shaped” but more worn-down at the apex. This tooth is the only premaxillary tooth with a lingual resorption pit at its base, which is surrounded by longitudinal striations. Medial to this tooth, a small conical element projects from ventral floor of the tooth row, possibly representing the tip of a replacement tooth. Denticles are present in both carinae of the fourth premaxillary tooth, but only in the distal carina of the third. These two teeth also display “leaf-shaped” crowns, but this condition is more marked in the fourth tooth, which has a comparatively more convex mesial margin and more strongly sigmoid distal margin. Denticles are well preserved only in the distal carina of the fourth tooth, where at least eight units are visible over a c. 2 mm long margin, which represents the concave apical portion of the carina. The largest of the denticles is about 0.2 mm broad, and they are all angled 45° relative to the distal margin of the tooth. Subtle denticles are observable within the mesial carina of the fourth tooth and the distal carina of the third tooth, being also restricted to the apical part of their crowns.

#### Maxillary teeth (Figs 3, 4, 14A and 14B)

**Fig 14:**
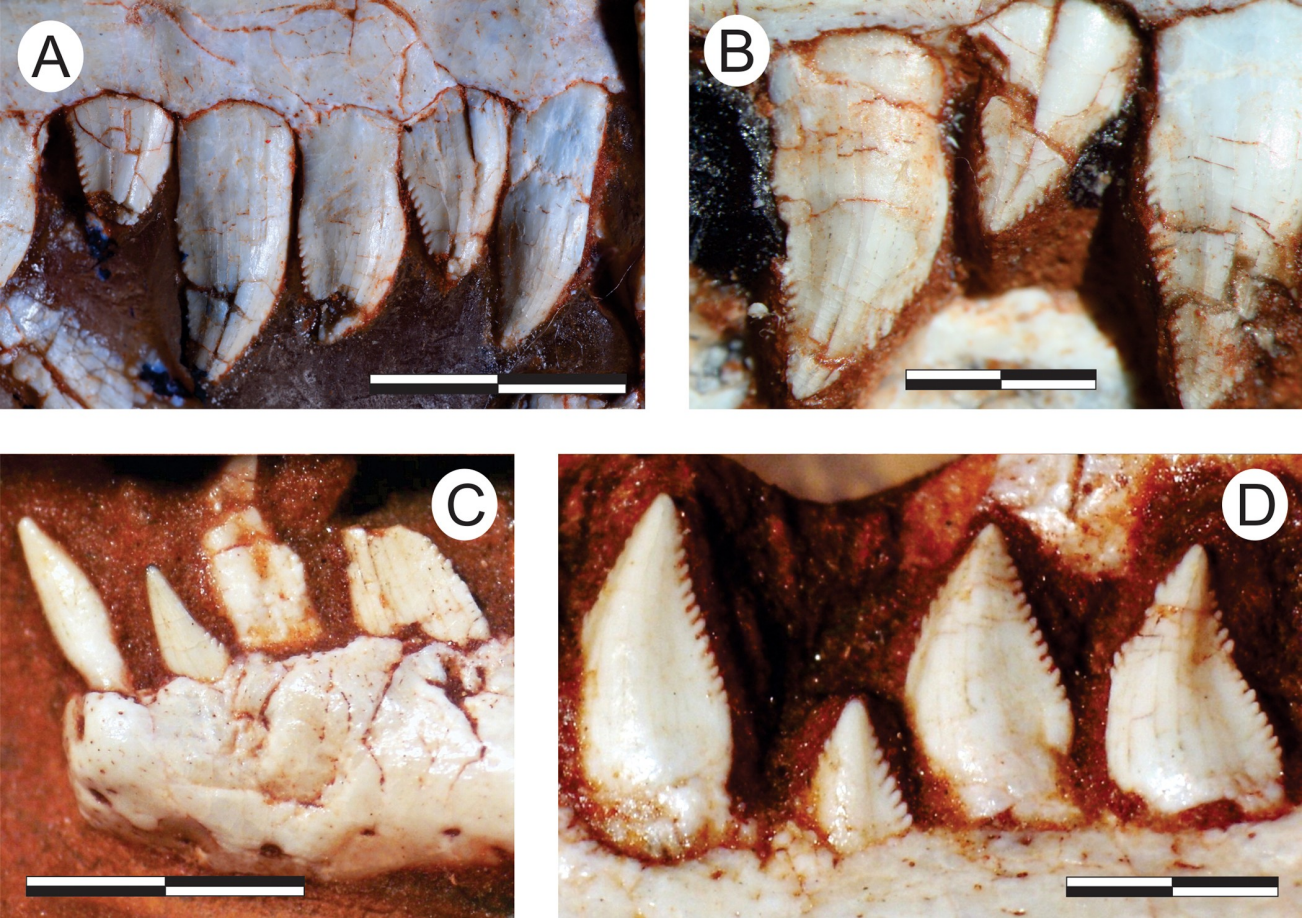
*Pampadromaeus barberenai* (ULBRA-PVT016), teeth. Details of the marginal dentition in lateral view. Right maxillary teeth 1–5 (A) and 7–9 (B); left dentary teeth 1–4 (C) and 9–12 (D). Scale bars = 5 (A,C), 2,5 (B,D) mm.

Except for the tenth element, the first 15 tooth crowns are well exposed in lateral view, many of which are also visible in medial view. Caudal to these teeth, three fragmented crowns (optimally observed in medial view), preceded by the concave margins of three alveolar sockets, indicate the presence of six additional teeth. Hence, *Pam*. *barberenai* had a minimum of 21 maxillary teeth. The lingual side of these teeth is no longer accessible following preparation of the lateral surface of the skull, and thus traits from this side are described based only on photographs. All teeth are labiolingually flattened, with well-developed mesial and distal carinae. Denticles are present in both carinae of all teeth and are obliquely oriented (about 45°) relative to the margin of the tooth (Fig 14). The general labial/lingual outline of each tooth is leaf-shaped, with a convex mesial margin and a sigmoid (convex at the base and concave apically) distal margin. In some teeth (e.g. 1, 3, 9, 15) the convex mesial margin is divided into basal and apical planes separated by a more marked inflection point. The former is generally straighter, perpendicular to the alveolar margin, and lacks serrations. The more apical segment is deflected ~ 45° clock-wise, is typically more convex, and bears denticles. Consequently, denticles are frequently restricted to the apical part of the mesial carina. Other teeth have a more continuously convex mesial margin, although there is no clear pattern regarding the distribution of these slightly different morphologies along the maxillary tooth series.

The first tooth is preserved slightly displaced from its original position in the alveolus, so that the apical part of the root is exposed. A subtle constriction is observable (in labial view) at the root/crown limit. The root slightly expands (mesiodistally) towards its base in labial view, whereas the crown (2,8 mm tall, 2 mm broad at the base) displays the leaf-shaped outline described above. Six denticles span a ~ 1 mm area of the apical third of the mesial carina. The distal carina possesses a minimum of eight denticles along a ~ 2 mm stretch of its apical (concave) margin, which are similar in size to those of the mesial carina. The labial surface of the crown is slightly expanded labially at the base, which is continuous with a subtle longitudinal bulge (Fig 14A). Judging from its position, the second tooth appears to be within the final stages of eruption. The crown is 3,8 mm tall and 2,5 mm broad at the base. Its tip is broken, but is clearly still observable in earlier images of the element taken in lingual view. Nine denticles are present in the apical-most 2 mm of the distal carina. Although denticles of the mesial carina are not so well preserved, they appear somewhat smaller and restricted to the apical-most part of the crown. The second tooth crown labially overlaps the distal margin of the third tooth crown and its labial surface lacks clearly defined longitudinal and basal bulges. The third tooth has an exposed root/crown juncture, where a marked mesiodistal constriction is seen. The root expands towards the base from that juncture and the crown is leaf-shaped (3,2 mm tall, 2,1 mm broad at the base). There is a basal bulge in both sides of the crown, and its distal quarter is labiolingualy compressed, so that longitudinal bulges are formed both lingually and labially. Denticles are easily discerned only in the distal carina, with four of them preserved in a ~ 0,7 mm stretch. A well-defined reabsorption pit is seen at the lingual surface of the root, bound mesially and distally by bone outgrowths that connect them to the lingual side of the alveolar margin. Concentric lines radiate from the labial side of the alveolus of the third and fourth teeth. The latter has a less marked crown/root constriction, and the distal margin of the crown is nearly straight in labial view, although a convex base and subtly concave denticulate margin is visible in lingual view. The crown is 4,0 mm tall and 3,0 mm broad at the base. Fourteen denticles are present on the distal margin, occupying most of the carina (3,5 mm) in lingual view. Mesial denticles are only visible in lingual view, six of which occupy a ~ 1,5 mm stretch of the apical half of the crown. As in the previous tooth crown, the distal quarter of the crown is labiolingualy compressed, forming subtle lingual and labial longitudinal bulges. Yet, the basal bulge is clearly seen only in the lingual surface of the crown. A resorption pit is seen lingually.

Less than half of the fifth tooth crown (3,5 mm tall, 2,3 mm broad at the base) is erupted. The distal carina is straight and the mesial convex. As better seen in lingual view, five denticles occupy the apical half (c. 1,5 mm) of the mesial carina. Seven denticles are seen in the basalmost 1,2 mm of the distal carina, apical to which the crown is broken. A subtle longitudinal bulge is present on each (lingual and labial) surface of the crown. It is clear, in lingual view, that the mesial margin of the fifth tooth lingually overlaps the distal margin of the fourth. The sixth and seventh tooth crowns are exposed only in labial view, their lingual surfaces being covered by the indeterminate palatal bone next to the left palatine. The former has a subtle crown/root constriction and a leaf-shaped crown (3,0 mm broad at the base) with a basal bulge and the apical third missing. Its distal carina overlaps labially the mesial margin of the succeeding tooth and bears 10 denticles along its basalmost c. 2 mm. The seventh tooth has subtle crown/root constriction and bulge at the base of the crown (4,2 mm tall, 2,5 mm broad at the base), but the longitudinal bulge is not as clear. Its mesial margin is poorly preserved, but has a general convex outline in labial view. In contrast, the distal margin has a small convexity at the base and is straight to subtly concave apically. Mesial denticles are not preserved, but at least 15 of them occupy a c. 3,0 mm segment of the distal carina. The eighth tooth is only partially erupted and poorly exposed lingually. In labial view, the crown (3,5 mm tall, 2,2 mm broad at the base) has a subtriangular outline, with subtly convex distal and concave mesial margins, with a clear longitudinal bulge. About four denticles are preserved within a 1 mm stretch of the apical part of both carinae, which are eroded basal to that.

The ninth and eleventh teeth are only partially exposed lingually, with their apical portions covered by the left palatine, but both show well-developed resorption pits and bone outgrowths surrounding them. The ninth tooth has a very subtle crown/root constriction. The crown (3,0 mm tall, 2,1 mm broad at the base) has a convex mesial margin and the distal margin has a small convexity at the base and is concave apically. The basal bulge is not marked, but the constriction of the distal fourth of the crown and the longitudinal bulge are clearly seen. This tooth bears about five denticles in a c. 1,2 mm segment towards the apex of the mesial carina, whereas a c. 2,8 mm stretch of the distal carina bears ten denticles. This tooth reflects a pattern that is also hinted by other elements (although not so clearly), especial towards the caudal part of the maxillary dental series, in which denticles on the distal carina tend to be smaller. The eleventh tooth is constricted at the crown/root juncture and has a somewhat shorter leaf-shaped crown (3,9 mm tall, 2,0 mm broad at the base) and a well-defined constriction of the distal fourth of the crown, as well as basal and longitudinal bulges. Denticles are broken off in the mesial carina, but five of them are seen on a c. 1 mm segment at the base of the distal carina. These are as small as those of the distal carina of the previous tooth. The teeth caudal to eleventh maxillary element are only exposed labially. The twelfth tooth crown is mostly broken, but a preserved part of the mesial carina bears some denticles. The 13th tooth is not completely erupted, so the crown/root transition is not exposed. It has a flattened labial surface, with unclear bulges. The crown is 3,0 mm tall and 2,0 mm broad at the base. Denticles are well-preserved within the partially erupted thirteenth tooth, with eleven of them visible along almost the entire length of the distal carina (1,8 mm), whereas only six occupy a c. 1 mm segment of the apical half of the mesial carina. The 14th tooth has a marked crow/base constriction and the crown has well developed basal and longitudinal bulges. The latter is, however, more mesiodistally expanded as the distal labiolingual constriction occupies less than a quarter of the crown. The crown (3,0 mm tall, 2,0 mm broad at the base) is leaf-shaped in labial view. Its apical part is missing, along with most of the denticulate part of the mesial carina. The preserved part of the distal carina (slightly more than a half of it) is 2,7 mm long and bears about 12 small denticles. The crown-base constriction and the labial bulges are subtle in the fifteenth maxillary tooth, the last that yields descriptive information The crown (3,2 mm tall, 1,8 mm broad at the base) has denticles preserved only on the apical half of the mesial carina, with five of them occupying a c. 1 mm segment.

#### Dentary teeth (Figs 11, 12A, 12B, 14C and 14D)

As in the upper jaw, all dentary teeth of *Pam*. *barberenai* are labiolingually flattened, with well-developed mesial and distal carinae. They are exposed only labially in both the right and left bones. The first tooth of the left dentary (Fig 14C) differs significantly from the more caudal elements. It is procumbent, slender (c. 3,5 mm tall; 1,0 mm of maximum breadth), and lacks denticles altogether. Both medial and distal margins of the crown are convex, but straighter in the tapering tip. The crown-root junction is mesiodistally constricted, and the exposed part of the root narrows down from that point. The second tooth is not fully erupted, so the shape of its entire crown is not possible to infer. The pointed tip and exposed mesial carina lack denticles, but four of these are seen in the basal-most 0,7 mm of the distal carina, forming ~ 45° angles to the crown margin. The third dentary tooth has a peculiar shape, which can be enhanced by the bad preservation of its margins. The crown (c. 4 mm tall; 2 mm of maximum breadth) is generally lanceolate, with sigmoid (concave apically and convex basally) distal and convex mesial margins, but its tip is atypically curved backwards. A pair of oblique denticles are seen in the maximal inflection of the convex portion of the distal margin, but other parts of the carinae are not well preserved enough to clearly infer the presence or absence of these elements. The fourth tooth is also incompletely erupted, but seems very different in shape compared to the second tooth. It is mostly subtriangular in lateral view, with straight mesial and distal carinae and a broken tip. No denticles are seen in the medial margin, but a 0,7 mm stretch of the distal margin bears four oblique elements. The broken front piece of the right dentary is inferred to have held four teeth. The base of the putative second and third elements are preserved in their alveoli, whereas the broken tip of the second tooth was displaced rostrally (Fig 12B).

Teeth five to twelve of the left dentary have a general shape shared with those same teeth in the right element, and are here described together. The completely preserved and erupted crowns are about four millimetres long and two millimetres broad (mesiodistally), with no significant increase or decrease in size along the series. The labial/lingual outline of the crowns is generally lanceolate, with convex mesial and sigmoid distal margins, so that the crown is mesiodistally expanded at the base, as well as tapering and caudally curved apically. In general, the labial surface of those teeth bears an elongate bulge that arches mesially as it extends apicobasally along the crown. This is shaped by the labiolingual compression of the crown margins, more invasive on the crown at the convex portion of its distal margin. That bulge is confluent to the general lingual transverse bulging of the crown base. Denticles are seen in all adequately preserved carinae, but absent from the most basal portions of the tooth margins, particularly in the mesial carina. Denticles are generally larger in the mesial carina, with five teeth per millimetre, forming low angles to the crown margin. Comparatively, denticles are smaller in the distal carina (about six per millimetre) and form steeper angles to the crown margin. These are usually close to 45°, but may reach near right angles near the tooth apex. The thirteenth tooth of the right dentary is not as elongated as the previous ones, but not much less mesiodistally broad. This results in a bulkier element, with a distal margin that is mostly convex in labial/lingual views and a less marked longitudinal budge. In the left dentary, the four caudal-most preserved teeth (14–16, 18) generally match this pattern. Crown overlapping is clear between teeth 8/9, 10/11, and 13/14 of the left dentary, and between teeth 11/12 of the right bone. In all cases, the distal margin of the preceding tooth labially overlaps the mesial margin of the following. Attachment bone forms a ring around the fifth tooth crown of the left dentary and around the eleventh of the right bone.

### Axial skeleton

Remains of 35 vertebrae are preserved, representing about half the total number of vertebrae that *Pam*. *barberenai* had. These are found scattered throughout the main block, with a prevalence of tail and caudal trunk elements. Missing elements were probably lost by syndepositional mechanical transport, which appears to have primarily affected the cranial part of the vertebral column. Likewise, ribs from the trunk area are more common than those from the neck. Preserved vertebrae are mostly isolated, with only minor articulated segments. The whole vertebral sample includes the isolated right atlantal neurapophysis, most of the atlas/axis complex articulated to a badly preserved third cervical vertebra, eleven isolated trunk vertebrae in various degrees of preservation (five still preserved in the main block and six prepared out), two articulated sacral vertebrae, and 17 vertebrae from different parts of the tail (four isolated, two pairs, and three sets of three articulated elements), as well as two centrum fragments freed from the main block that are probably referable to the trunk series and various neck/trunk ribs and haemal arches.

#### Neck (Fig 15)

**Fig 15:**
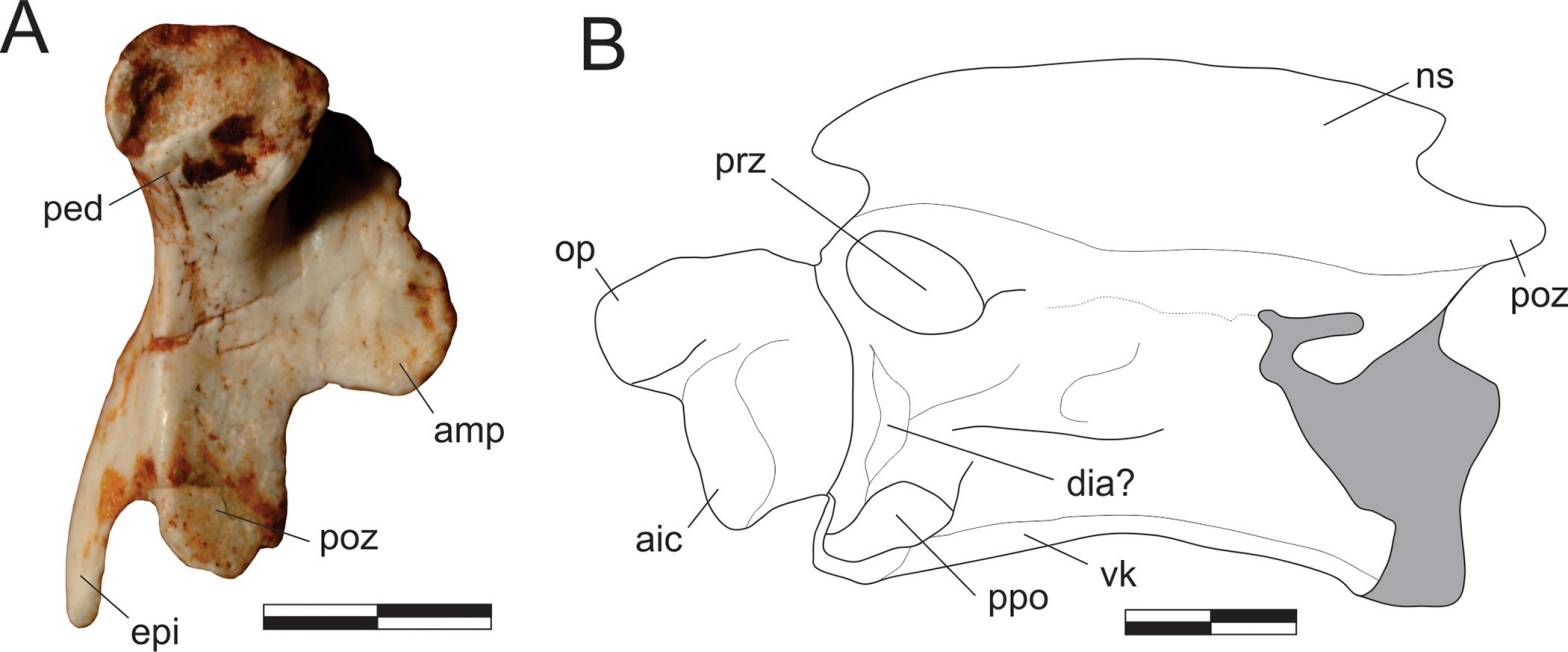
*Pampadromaeus barberenai* (ULBRA-PVT016), atlas-axis complex. Photograph (A) and drawing (B) of the atlas-axis complex. A, atlantal neurapophysis in ventral view; B, part of the atlas/axis complex in left lateral view. Abbreviations: aic, axial intercentrum; amp, atlantal medial process; dia, diapophysis; epi, epipophysis; ns, neural spine; op, odontoid process; ped, pedicel; poz, postzygapophysis; ppo, parapophysis; prz, prezygapophysis; vk, ventral keel. Grey areas indicate broken surface. Scale bar = 5 mm.

The right atlantal neurapophysis (Fig 15A) is preserved in ventral view within the main block. It has a spur-like epipophysis (but not as pointed as in *Herrerasaurus ischigualastensis* [77]; note that the atlantal neurapophysis was mistakenly identified in that paper as a right element and Fig 11D corresponds to a ventral view) that projects caudodorsally, extending slightly beyond the caudal margin of the postzygapophysis. The postzygapophyseal articular facet faces ventromedially and is elliptical in outline, with the long axis of the ellipsis oriented craniocaudally. The postzygapophysis is well-differentiated, forming a 90° angle from the caudal margin of the medial process in ventral view [78]. This process extends medially as a dorsoventrally flattened, fan-shaped element, forming the roof of the neural canal. However, as the medial margin is incomplete, it is uncertain if it reached its partner. The pedicel is restricted to the cranial portion of the neurapophysis. It surrounds the neural canal laterally, extending ventrally as a column that expands at its base, where it articulated to the atlantal intercentrum.

Apart from the atlantal neurapophysis, the only preserved cervical elements are the atlas/axis complex (Fig 15B) articulated to the following vertebra. These were recently prepared and not mentioned in the original description [14]. Only the left side of the axial neural arch is visible, although most of the centrum is free of surrounding matrix. The neural spine extends along the craniocaudal length of the element. It has a convex dorsal margin in lateral view, with the dorsal apex of the spine at roughly its craniocaudal midlength. Both cranial and caudal ends of the neural spine terminate as sharp hook-like extensions, with the cranial and caudal margins sloping back towards the main body of the axis. Although partially obscured by matrix, it appears that the caudal end of the neural spine bifurcated into short, laterally projecting arms. This same obstruction makes it difficult to confirm the presence of an epipophyseal ridge above the postzygapophysis; however, if present, it does not appear to have been particularly pronounced. The postzygapophysis appears to have been attached to the axial body via a low laminar ridge that extends cranioventrally, although this has potentially been exaggerated by poor preservation in this region of the element. The prezygapophysis extends craniolateroventrally from beneath the cranial margin of the neural spine as a pronounced lobe-shaped projection. As in the postzygapophysis, a low laminar ridge extends along the caudoventral margin of the prezygapophysis, connecting it with the axial body. Directly below the prezygapophysis, on the cranial margin of the centrum and ventral to its dorsoventral midpoint, the parapophysis is present as a stout, ventrolateral projection with a triangular-shaped terminal surface. The diapophysis is possibly represented by a subtle ridge extending dorsally from the craniodorsal corner of the parapophysis. The craniocaudal length (c. 20mm) of the axial centrum is approximately twice the dorsoventral height (c. 10mm) of its cranial end. The ventral surface of the centrum bears a pronounced keel that bifurcates caudally, producing a distinct drop-shaped ventral fossa. Both the bifurcation and attendant fossa are restricted to the caudal half of the centrum.

Both the odontoid process and axial intercentrum are preserved on the cranial face of the axial centrum. The ventral surface of the axial intercentrum, at the juncture of the cranioventral margin of the axial centrum, is extensively excavated by a deep fossa. However, this is most probably the product of poor preservation and/or temerarious preparation. The intercentrum is subequal in transverse width to the axial centrum, and separated from the latter via a shallow neck. The odontoid process is visible in ventral view, projecting from the dorsal margin of the intercentrum as a rectangular wedge of bone that is about half the transverse width of the intercentrum. Its dorsal surface is relatively flat and without obvious signs of a natural concavity.

The only exposed portion of the third cervical vertebra is the left side of the neural arch (above the low ridge connecting the zygapophyses), but it is clear that the vertebra is longer than the axis (neural arch craniocaudal length = 25mm). The neural spine is craniocaudally elongate, very low, and bifurcates at the caudal end. It clearly did not extend along the entire length of the element, as observed in the axis, and lacks any dorsal projections and raised borders. Similar (albeit more pronounced) to the condition in the axis, the cranial and caudal ends of the neural spine terminate in sharp spur-like projections. It is possible that a low epipophyseal ridge was present upon the dorsal surface of the postzygapophysis, although poor preservation makes this difficult to confirm. The prezygapophysis projects well beyond the cranial margin of the neural spine. In contrast, the caudal projection of the postzygapophysis beyond the caudal margin of the neural spine is proportionally much less pronounced.

#### Trunk (Figs 16 and 17)

**Fig 16:**
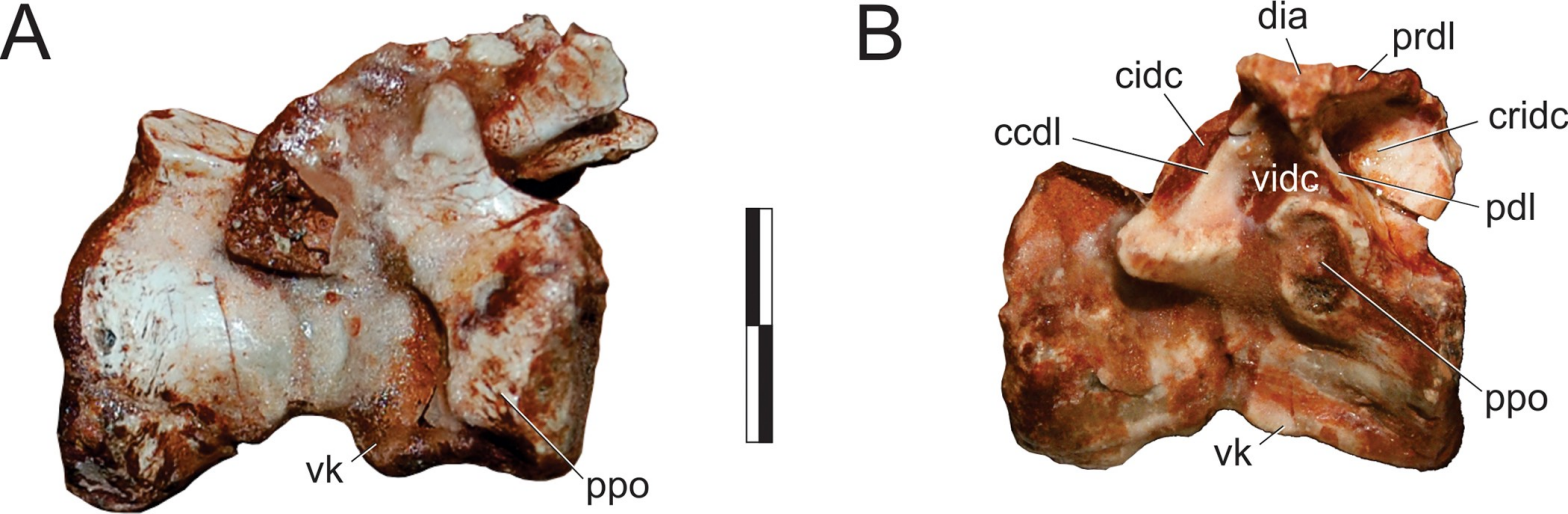
*Pampadromaeus barberenai* (ULBRA-PVT016), trunk. Cranial trunk vertebrae, photographs in lateral view. Abbreviations: ccdl, caudal centrodiapophyseal lamina; cidc, caudal infradiapohyseal chonos; cridc, cranial infradiapohyseal chonos; dia, diapophysis; pdl, paradiapophyseal lamina; ppo, parapophysis; prdl, prezygodiapophyseal lamina; vidc, ventral infradiapohyseal chonos; vk, ventral keel. Scale bar = 1 cm.

Two of the recovered trunk vertebrae belong to the cranial fourth of the series: one was extracted from the main block, but is adhered to the craniodorsal end of the scapular blade; the other remains in the block near a set of haemal arches. The identification of the latter (Fig 16A) is hampered by its poor preservation, as most of its neural arch is missing. Nonetheless, the presence of a ventral keel, the position of the parapophysis in the lower part of the cranial margin of the centrum, and a caudal articular face of the centrum that is ventrally displaced relative to the cranial face, suggests that it represents a vertebra from the cranial-most part of the trunk, or even the caudal part of the neck. The craniocaudal length of the centrum (24 mm) is 1.8 times the dorsoventral depth of the caudal articular face, and 2.0 times that of the cranial face. In the element adhered to the scapula (Fig 16B), the parapophyses have not yet risen entirely from the centrum to the neural arch, and thus the vertebra is likely from the first third of the trunk, but more caudal than the previous one. It lacks the postzygapophyses, the cranial tips of the prezygapophyses, and the neural spine is covered by sediment. The bases of the broken prezygapophyses bear flat/vertical medial margins, which may correspond to the hypantral articulation facets. The assortment of diapophyseal laminae (prezygodiapophyseal, caudal centrodiapophyseal, and paradiapophyseal laminae) are in strong evidence, and bound deep cranial, ventral, and caudal chonoe. The postzygodiapophyseal laminae have not been preserved along with the postzygapophyses. The diapophyses are oriented mainly laterally, albeit with a subtle dorsal inflection. The only observable (right) parapophysis is ovoid in shape and situated well-back from the cranial margin of the centrum, although still within its cranial half. Its dorsal half is positioned on the neural arch, whereas the ventral half is located on the centrum, although no clear vestige of the neurocentral joint is seen. The craniocaudal length of the centrum (20 mm) is 1.8 times the dorsoventral depth of the caudal articular face, and 2.2 times that of the cranial face. Both articulations are as lateromedially broad as deep, although the latter is potentially taphonomically warped. A strong ventral keel extends along most of the length of the centrum, and is more pronounced cranially than caudally.

**Fig 17:**
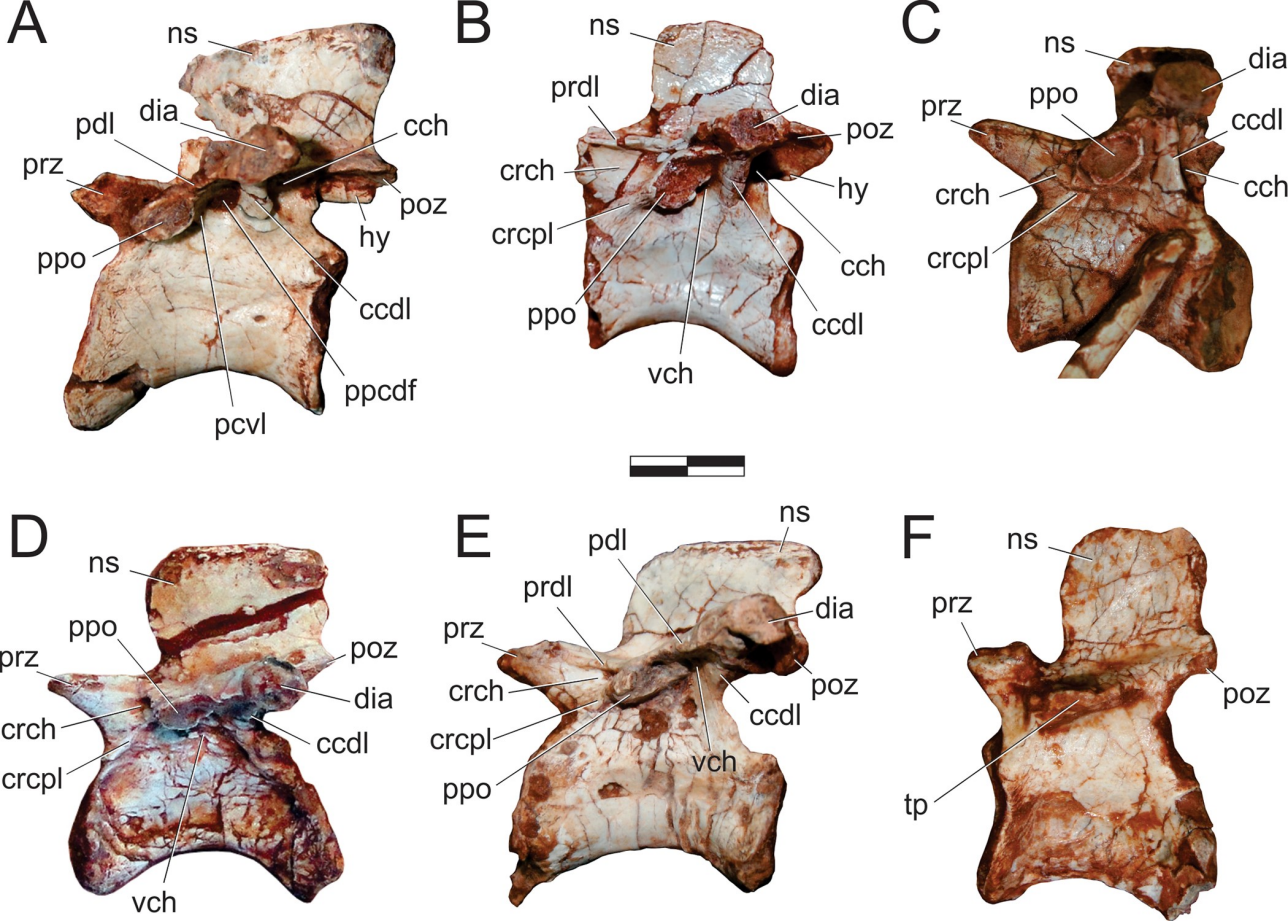
*Pampadromaeus barberenai* (ULBRA-PVT016), trunk. Mid-caudal trunk vertebrae, photographs in lateral view (C and D reversed). Abbreviations: ccdl, caudal centrodiapophyseal lamina; cch, caudal chonos; crch, cranial chonos; crcpl, cranial centroparapophyseal lamina; dia, diapophysis; hy, hypantrum; ns, neural spine; pcvl, parapophysis caudoventral lamina; pdl, paradiapophyseal lamina; poz, postzygapophysis; ppo, parapophysis; ppcdf, parapophyseal centrodiapophyseal fossa; prdl, prezygodiapophyseal lamina; prz, prezygapophysis; tp, transverse process; vch, ventral chonos. Scale bar = 1 cm.

All the remaining trunk vertebrae have parapophyses located entirely on the neural arch, and probably belong to the caudal three-fourths of the series (see [34–35]). Five of them have been prepared free of the excavation block, three of which are mostly complete (Fig 17A, 17E and 17F). One of the remaining two is represented by the neural spine and the central/caudal part of the left neural arch, whereas the other represents the cranial half of the centrum and a partial neural arch. The main block preserves four additional trunk vertebrae, with the majority observable only in lateral view (Fig 17B–17D). These form a loose cluster within the quadrant of the block typified mainly by the large scatter of trunk ribs. Centrum length of these vertebrae range from 18 to 24 cm, roughly representing 1.5–1.7 times the dorsoventral height of their articular faces. One exception is the probably caudalmost of them (Fig 17F), in which this metric is about 1.3. The shape of the caudal articulations range from ovoid (12 cm deep, 7 cm broad) to nearly circular (12 cm deep, 11 cm broad), so that all preserved trunk vertebrae are relatively lateromedially compressed. It is uncertain how much of this may have been exaggerated by preservation, as most of them are found with their sagittal plane parallel to the surface in which the bones are scattered (i.e. likely corresponding to the bedding surface). The abovementioned isolated centrum with fragmentary neural arch has a cranial articular face slightly broader than high.

The ventral surface of all mid-caudal trunk centra are craniocaudally concave and bear only shallow lateral depressions. One centrum has a somewhat deeper, longitudinally elongated depression at the dorsal part of its lateral surface (Fig 17B). None of the mid-caudal trunk elements appear to have possessed a distinct ventral keel. One centrum (Fig 17A) bears a foramen in its caudoventral portion. There is no clear evidence of suture lines between the centra and neural arches in any of the mid-caudal trunk vertebrae. The neural spine craniocaudal length of most mid-caudal trunk vertebrae (Fig 17A, 17D and 17E) is about 1.5 times the dorsoventral height. In contrast, the spine in some vertebrae is proportionally taller (Fig 17B and 17C), including one more caudally positioned (Fig 17F), with a dorsoventral height nearly equivalent to the craniocaudal length. All neural spines are roughly squared/rectangular in lateral view, with their dorsal margins rugose and slightly expanded laterally, but not forming a spine table. The caudal profile of most of the trunk neural spines is slightly concave in lateral view (Fig 17D and 17E), with the caudodorsal corner more acutely developed than the craniodorsal. The dorsal surface of the neural spine of one vertebra (Fig 17A) is inclined anteriorly, but this is likely due to deformation, as the centum has also suffered a degree of craniodorsal to caudoventral compression.

The dorsoventral height of the neural arches (when measured from the neurocentral junction to the zygapophyseal facets) is slightly less than the height of the caudal face of the respective centrum in all adequately preserved mid-caudal trunk elements; but the arch seems lower in the more caudal elements. Although poor preservation and adherent matrix makes it difficult to determine the condition of the neural canal in most vertebrae, it appears to have been relatively cylindrical in more cranial elements, with more caudal vertebrae having a comparatively dorsoventrally elongate (‘slot-shaped’) neural canal. The postzygapophyseal facets of all preserved mid-caudal trunk vertebrae are oriented mainly on the horizontal plane with, at most, a very subtle dorsal deflection of its lateral margin. In contrast, the prezygapophyseal articular facets slope inwards as a ‘V’ with sides forming angles of approximately 40° to the horizontal plane. A distinct hyposphenal ridge can be seen in some of the best preserved mid-caudal trunk elements (Fig 17A and 17B). They project vertically and are not lateromedially expanded at the ventral part.

The diapophyses/transverse processes of the mid-caudal trunk vertebrae are caudolaterally oriented, perhaps more so in the more caudal elements. Most also present a slight dorsal inclination, and narrow slightly (craniocaudally) as they extend laterally. The parapophysis and diapophysis are clearly separated in three of the best preserved mid-caudal trunk vertebrae (Fig 17A–17C), with the former positioned cranioventrally and connected to the latter via a prominent paradiapophyseal lamina. In contrast, in two more caudally positioned vertebrae (Fig 17D and 17E) these apophyses are aligned along nearly the same dorsoventral plane, with the parapophysis joining the base of the rod-like diapophysis. In the putatively caudal-most of the preserved trunk vertebra (Fig 17F), the transverse processes are not well preserved, but both apophyses seem to form a single, nearly horizontal platform. The three more cranial mid-caudal trunk vertebrae (Fig 17A and 17C) have prominent postzygodiapophyseal and caudal centrodiapophyseal laminae bounding the caudal chonos, as well as a cranial centroparapophyseal laminae ventrally delimiting the cranial chonos. Prezygoparapophyseal lamina are clearly absent from one of those vertebrae (Fig 17B), and cannot be confirmed in the remaining mid-caudal trunk vertebrae due to poor preservation (Fig 17A) and/or high placement of the parapophyses (Fig 17C). Indeed, the parapophysis may be connected to the prezygapophysis by a marked lamina, which dorsally roofs the cranial chonos (Fig 17B), but this probably represents the prezygodiapophyseal lamina, which has merged with the dorsal margin of the parapophyses due to the dorsal migration of the latter. Two of those vertebrae (Fig 17A and 17C) also bear a less prominent lamina extending caudoventrally from the parapophysis (“pcvl” in Fig 17A), dividing the usually shallow ventral chonos into smaller cranioventral (centroparapophyseal fossa of Wilson *et al*. [79]) and deeper caudodorsal (parapophyseal centrodiapophyseal fossa of Wilson *et al*. [79]) depressions. In two more caudal trunk vertebrae (Fig 17D and 17E), the parapophysis is cranioventrally adjacent to the diapophysis end elevated into the transverse process. This process is buttressed by well-developed cranial centroparapophyseal, prezygodiapophyseal, postzygodiapophyseal, and caudal centrodiapophyseal lamina. Because the diapophysis is set well dorsal and backwards, the caudal centrodiapophyseal lamina of those vertebrae is nearly vertical and the caudal chonos deflected mainly to the caudal surface to the vertebra. As a consequence, the ventral chonos occupies most of the lateral surface ventral to the transverse process. In the most caudal trunk vertebra (Fig 17F) only a subtle lamina is seen connecting the craniodorsal corner of the centrum to the transverse process, setting the limits of shallow cranial and ventral chonoe. The cranial neural canal aperture of that vertebra is subrectangular, one third deeper than broad. In all mid-caudal trunk vertebrae in which the corresponding area is preserved, the medial wall of the cranial and caudal chonoe is formed by the pedicel itself, with no additional bracing extending from the zygapophyses.

#### Sacrum (Fig 18)

Only two vertebrae are intimately associated into the sacrum of *Pam*. *barberenai*. These are well-preserved, lacking only parts of the transverse processes and ribs. The entire structure suffered some dorsal/right to ventral/left compression, so that the bones are displaced along that plane. The combined craniocaudal length of the lateral articular surfaces of the sacral ribs for both conjoined vertebrae is c. 6 cm. This closely matches the available area for rib articulation on the medial surfaces of both ilia, confirming that these elements likely represent the primordial vertebral pair of the archosaur sacrum. There are no preserved vertebrae caudal or cranial to these vertebrae, as well as no isolated vertebra within the assemblage that clearly fits the expected morphology of caudo- or truncosacral vertebra. The only possible evidence for additional vertebral incorporation into the sacrum is the presence of a rounded scar craniodorsal to the articulation of the first main sacral rib (near the dorsal margin of the lamina) on the left ilium, which may have hosted a reduced articulation for a trucosacral vertebra (or ‘sacralised’ trunk vertebra).

**Fig 18:**
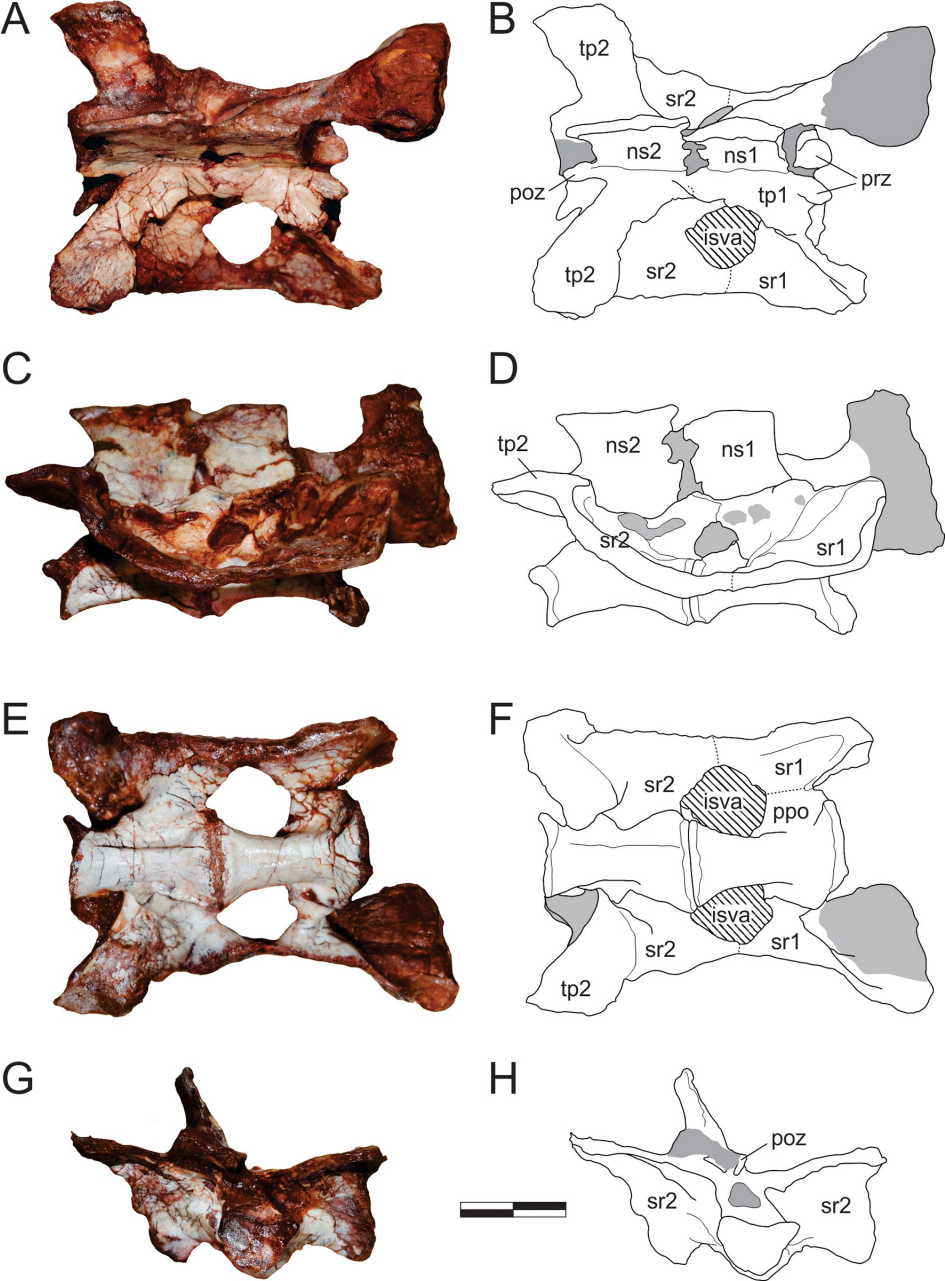
*Pampadromaeus barberenai* (ULBRA-PVT016), sacrum. Photographs (A,C,E,G) and drawings (B,D,F,H) of the sacrum in dorsal (A-B), right lateral (C-D), central (E-F), and caudal (G-H) views. Abbreviations: isva, intercostal space ventral aperture; ns, neural spine; poz, postzygapophysis; ppo, parapophysis; prz, prezygapophysis; sr, sacral rib; tp, transvers process. Numbers in the elements indicate correspondence to either sacral vertebrae. Dashed lines indicate inferred skeletal margins; grey areas indicate surfaces that are broken or covered with sediment. Scale bar = 2 cm.

The craniocaudal length (about 23 mm, slightly longer in the first) of both sacral centra is about twice that of the lateromedial and dorsoventral breadths of their articular faces. The cranial articular face of the first vertebra is slightly broader than deep, a condition exaggerated in the caudal articular face of the second vertebra. Regardless of the overall shape, the cranial and caudal articulations of the first sacral centrum are subequal in lateromedial breadth, but the caudal margin of the second centrum is significantly narrower. Both centra have concave ventral and lateral margins and lack ventral keels. Both the neurocentral junctions as well as the sutures with the respective ribs are easily observable. The ribs are positioned upon the cranial margin of the centra, arising from enlarged and laterally projected parapophyses. In contrast to the first sacral vertebra, the attachment of the sacral rib to the second sacral vertebra is much more craniocaudally extensive, extending from the cranial margin of the centrum to well-beyond its craniocaudal midpoint. On its left side, the first sacral centrum has a deep, craniocaudally elongated, eye-shaped furrow immediately caudal to the parapophysis and ventral to the neurocentral junction, which occupies most of its remaining middle section. However, no equivalent feature is observed on the opposite side.

In ventral view, the first sacral rib waists (craniocaudally) as it projects laterally from its contact with the centrum before undergoing a dramatic craniocaudal flaring, forming an expansive, fan-shaped sheet of bone that contacts with the ilium laterally and the second sacral rib caudally. In lateral view, the first sacral rib forms a “L” shaped articulation to the ilium. The articulation has a nearly straight, subhorizontal component that is slightly oblique (craniodorsally to ventrocaudally oriented) to the main axis of the centrum. This component cleanly articulates with all but the caudal corner of a straight, elongate facet that the crosses the ventral part of the medial surface of the iliac body, oblique to the line connecting the ventral tips of pubic and ischial peduncles (best seen in the left element). The cranial component of the articulation (best seen on the right side) expands dorsally to form the cranial wall of the sacral intercostal space, the cranial part of the ventral floor of which is formed by the subhorizontal caudal extension of the same rib. The lateral margin of the cranial wall fits cleanly into a subvertical groove on the medial iliac surface, immediately caudal to the ridge that extends from the pubic peduncle to the preacetabular ala. The lateral margin of the transverse processes of the first sacral vertebra is incomplete, precluding assessment of its participation in the iliac articulation. Nonetheless, the preserved (medial) portion of the right transverse processes shows that it is more craniocaudally extensive than the dorsal summit of the rib it covers, also expanding over areas caudal (sacral intercostal space) and cranial to that. If the transverse process extended as far laterally as its associated rib, as suggested by scarring on the medial surface of the ilium, the entire first sacral rib complex would have displayed the “C-shaped” iliac articulation typical to many other early saurischians (e.g. *Sa*. *tupiniquim* [28]). In ventral view, the suture between the two sacral ribs is craniocaudally level with the centre of the aperture (“isva” in Fig 18F) that pierces the floor of the intercostal between them.

In lateral view, the rib portion of the iliac articulation of the second sacral vertebra expands caudodorsally from the articulation point with the first sacral rib, forming a nearly straight oblique line whose cranial edge is slightly above the ventral margin of its respective centrum, whereas the caudal edge is well above the dorsal margin of the centrum. Caudally, the iliac articulation becomes nearly horizontal, corresponding to the portion of the rib-complex that is formed by the transverse process of the vertebra. Whereas the oblique rib articulation matches the sloping ventral margin of the cranial half of the postacetabular ala of the ilium, probably occupying a subtriangular facet cranioventral to the “posteromedial lamina/shelf”, the horizontal articulation of the transverse process fits onto a depression positioned above the cranialmost portion of that lamina/shelf (best seen in the right ilium). From its centrum articulation (medially) to that with the ilium (laterally), the second sacral rib expands as a fan-shaped structure, as seen both in ventral and caudal views. This forms most of the oblique wall that caudally bounds the sacral intercostal space. In dorsal view the transverse processes of the second sacral vertebra extend caudolaterally, with a slight craniocaudal expansion towards their lateral ends. As with the first sacral vertebra, the transverse process is more craniocaudally expanded than the dorsal summit of the respective rib. As such, its cranial margin partially roofs the sacral intercostal space, whereas is caudal margin extends beyond that of the rib summit. In caudal view, a (medially deeper) subtle furrow marks the transition of the oblique wall of the rib to the horizontal platform formed by the transverse process. Caudal to the rib articulation, the second sacral centrum bears a deep eye-shaped notch, similar to that of the first sacral element, and also only seen on the left side. It is shared amongst the centrum, the neural arch, and the caudal margin of the base of the rib.

The neural spines of both sacral vertebrae are tall (deeper dorsoventrally than craniocaudally long), subrectangular and, although lacking spine tables, undergo a slight lateromedial expansion towards the dorsal margin. The prezygapophyses of the first sacral element are lobe-like structures, held nearly horizontally, but facing slightly dorsomedially. The postzygapophyses are partially fused with the prezygapophyses of the second sacral vertebra, but a suture line between them is recognisable. An aperture between the neural arches of the two sacral vertebrae is seen in lateral view, below their fused zygapophyses and above the caudal edge of the first sacral centrum. The first sacral vertebra bears a subtle caudal centrodiapophyseal lamina.

#### Tail (Fig 19)

Among the 17 preserved caudal vertebrae, only one element corresponds to the proximal part of the series, but is preserved partially imbedded in the sediment, although isolated from the main block. It is relatively incomplete, lacking most of the neural spine, the prezygapopheses, the right transverse process, the proximal articular face, and most of the ventral surface of the centrum. Additionally, the postzygapophyses and the distal face of the centrum are obscured by the block of matrix that is acting as support for the element. The neural spine is represented by a thin sliver of bone encased in sediment. This clearly represents the distal edge of the spine and is suggestive of the dorsodistal orientation typical of caudal elements. The transverse process projects dorsolaterally and slightly distally. In dorsal view, the distal margin of the base of the transverse process houses a distinct concave embayment, with a shallower embayment also present on the base of the proximal margin. Thus, the transverse process appears to have possessed a basal ‘neck’ with a concurrent lateral expansion. The neural arch is possibly positioned on the centrum with a strong anterior bias, although poor preservation precludes a more precise understanding. The centrum, although incomplete, appears to have been relatively longer proximodistally than dorsoventrally tall.

**Fig 19:**
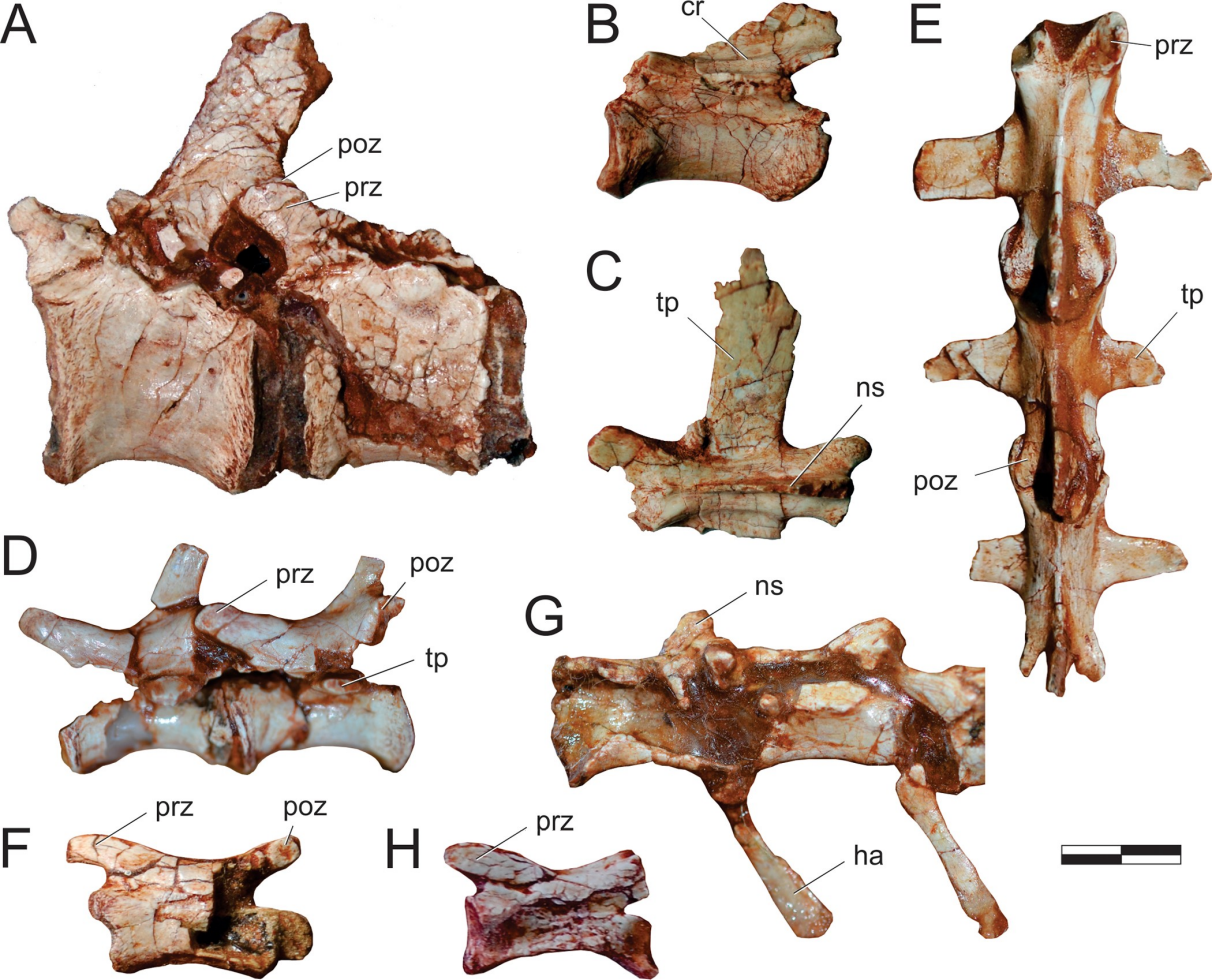
*Pampadromaeus barberenai* (ULBRA-PVT016), tail. Tail vertebrae, photographs in left (A-B,D,F) and right (G-H reversed) lateral, and dorsal (C,E) views. Abbreviations: cr, collateral ridge; ha, haemal arch; ns, neural spine; poz, postzygapophysis; prz, prezygapophysis tp, transverse process. Scale bar = 1 cm.

A pair of articulated caudal vertebrae (Fig 19A) extracted from the main block represent the mid-proximal part of the tail (around the 10^th^ caudal element). The neural spine of the better-preserved proximal element is the same dorsoventral length as the respective centrum and directed distodorsally. It terminates at a point immediately distal to the distal margin of the articular face of the centrum and does appear to have expanded proximodistally as it proceeds dorsally. The postzygapophyses are elevated on the distal margin of the neural spine and flush with the distal margin of the centrum, whereas the prezygapophyses project beyond its proximal margin. The transverse processes are mostly incomplete, but appear mainly short and laterodistally oriented on the left side of the distal element. As preserved, these centra are dorsoventrally taller than transversely wide, with a proximodistal length (c. 20 mm) 1.5 times their dorsoventral height. The ventral surfaces of the centra lack any kind of longitudinal ridges/keels and grooves/sulci and its distal end bears an oblique, flattened articulation facet for the chevron. The neurocentral junctions are closed.

The middle part of the tail (around the 15^th^ to 20^th^ caudal elements) is represented by an isolate vertebra extracted from the main block and a vertebral pair still imbedded in its matrix. The isolated vertebra (Fig 19B and 19C) lacks most of the dorsal length of the neural spine, the left transverse process and prezygapophysis. The neural spine is set well towards the distal end of the neural arch, although it extends proximally to the level of the prezygapophyses as subtle bifurcating ridges, with a small eye-shaped depression between them. The postzygapophyses are not elevated on the neural spine and their articular facets face lateroventrally. Extending between the zygapophyses, on either side of the neural spine and medial to the transverse processes, a pair of low collateral ridges (“cr” in Fig 19B) is observable. The right transverse process is well persevered and oriented laterally and slight caudally. It also undergoes a slight proximodistal expansion as it extends laterally. The articular facets are transversely broader (10 mm) than dorsoventrally high (9 mm), differing from the more proximal caudal vertebrae described above (possibly accountable due to taphonomic compression in either of the specimens). The ventral margin of the 19 mm long centrum is well preserved and clearly shows a pair of subtle ridges flanking a midline groove of equal subtlety. It would appear, therefore, that this feature is either variable along the caudal series, or characterises more distally located elements. As in the more proximal elements, an oblique, flattened articulation facet for the chevron is seen on the distoventral corner of the centrum. The neurocentral junction may not the completely closed, as subtle suture is recognisable. The centra of the two articulated vertebrae (Fig 19D) are about 15 mm long. They lack a ventral keel, bearing instead a flattened, almost (lateromedially) concave ventral margin, flanked by very subtle collateral ridges. Their neural spines are displaced distally on the neural arch, taller that proximodistally broad, slightly caudally inclined, and dorsally narrowing (proximodistally), but the dorsal margin is flatten in lateral view. The postzygapophyses are slightly elevated on the neural spine. The centrum and neural arch are separated by damage sustained by both elements; however, this does not follow the neurocentral suture, which is inferred to have been closed in these elements.

Vertebrae from the distal half of the tail (distal to the 20^th^ element) include thee articulated elements collected from the perimeter of the block and now preserved in a small plater jacket and other elements within the block. This includes a set of three articulated vertebrae, another set of three with a fourth associated element that is not articulated, and one isolated vertebra. The best preserved of these elements is the set of three vertebrae with the associated fourth, which likely represent the more proximal of the tail vertebrae descried from here onwards. The three articulate vertebrae (Fig 19E) are exposed mainly in dorsal view and bear laterally (not laterodistally) oriented transverse process. The neural spine is short, distally inclined, and continuous with a low ridge that extents proximally to bifurcate and reach the base of the prezygapophyses. The postzygapophyses are elevated on the neural spine. The associated fourth vertebra is from a more proximal position in the tail. Its centrum is 19 mm long, with a proximal articular face 11 mm deep and 12 mm broad. Its ventral surface bears a subtle longitudinal groove that is deeper towards the proximal end. The transverse processes are laterodistally directed.

The three distal tail vertebrae removed in a small plaster jacket are exposed on their left lateral surface. However, much of the surface is eroded and only the middle element (Fig 19F) is mostly complete. There is no sign of a transverse process, indicating that these elements are from a very distal part of the caudal series. The neural spine is highly reduced and positioned on the very distal part of the neural arch. The prezygapophyses extend somewhat beyond the proximal margin of the centrum, as is typical for early sauropodomorphs. The postzygapophyses are elevated on the small neural spine and their articulation facets are almost completely oriented towards the lateral. The centra are approximately three times longer (about 15 mm each) than high. The proximal portion of a small chevron is preserved between the first two elements. The set of three vertebrae mainly exposed in right lateral view (Fig 19G) within the block conform to the morphology of those just described. The proximal two are the best preserved, with centra of about 17 mm. The tail vertebra isolated in the block is exposed on its right side (Fig 19H), and probably represents the distalmost vertebra of the assemblage. Most of the neural arch and right surface of the centrum is broken, but both prezygapophises are well preserved. These expand proximal to the proximal margin of the centrum, which is 15 mm long.

#### Presacral ribs and chevrons (Fig 20)

There are at least two cervical ribs present in the block. Both of which have thin, rod-like straight shafts of about 4 cm. In the best-preserved element, the elongated tuberculum expands dorsocaudally. The spine is cranially tapering and over 5 mm in length. No capitulum is exposed for the cervical ribs. There are about a dozen preserved trunk ribs and trunk rib fragments, most of them clustered in a corner of the block. The best preserved of them are formed of a dorsally projecting tuber-like tuberculum, a longer medially expanding capitulum, and an elongated, slender shaft. Both the capitulum and the shaft are craniocaudally compressed, with an ovoid cross section (craniocaudal short axis). Beginning in the juncture between the tuberculum and the capitulum, a pronounced groove extends along the caudal surface of the shaft for most of its length. In cranial/caudal views, the strongest inflection point of the shaft is immediately distal to the tuberculum/capitulum juncture. Distal to that, the shaft is bows slightly outwards. There are eight preserved chevrons clustered in the centre of the block, four of them aligned between the prefrontal and the fibula. A mostly complete element is about 4 cm long. The saddle-shaped (convex proximodistally and concave lateromedially) articulation has an ovoid dorsal outline, with a lateromedially oriented long axis. In cranial/caudal views, the lateral margins of the articulation expand proximolaterally and have rugose lateral surfaces. As a result, the shaft is proportionally much narrower lateromedially. In lateral view, the articulation area expands distally, but not proximally, relative to the shaft. The haemal canal is dorsally closed in all available chevrons. The cranial aperture is slit-like, but the caudal is dorsally broader, resulting in a subtriangular outline. The chevron shaft is generally compressed lateromedially, but broader dorsally and ventrally tapering in cranial/caudal views. In lateral view, the shaft does not taper ventrally, and is even slight expanded at the ventral portion.

**Fig 20:**
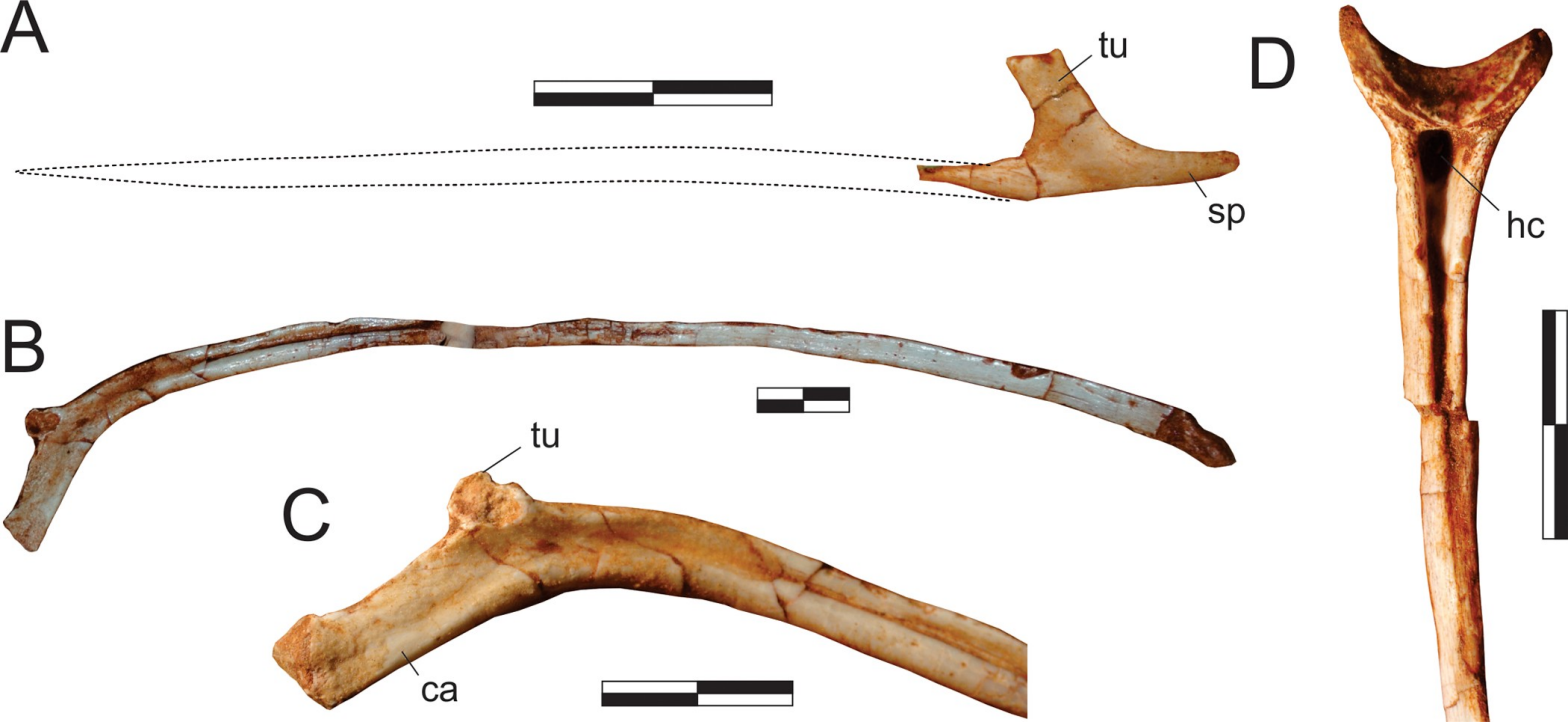
*Pampadromaeus barberenai* (ULBRA-PVT016) ribs and chevrons. Photographs of ribs and chevron. A, Cervical rib in lateral view; B-C, Trunk rib in caudal view. D, Chevron in caudal view. Abbreviations: ca, capitulum; hc, haemal canal; sp, spine; tu, tuberculum. Dashed lines indicate inferred skeletal margins. Scale bar = 1 cm.

### Scapular girdle and limb

Preserved elements of the scapular skeleton are found isolated, including right scapula, humerus, and ulna, and left scapula. The preservation of the scapula indicates that the scapulocoracoid is not coossified. No vestige of other forelimb bones has been recovered.

#### Scapula (Fig 21A)

The right scapula is nearly complete, preserved isolated in the main block, and exposed in medial view. Its left counterpart is also exposed in medial view, but most of the blade is missing. It agrees in all anatomical details with the right bone, which forms the basis for the following description. The scapula is lateromedially flatted and laterally arching, formed of a craniocaudally broad body (= *caput scapulae* [80]), and an elongated dorsal blade. The caudal margin of the blade is mostly straight, but curves caudally at its dorsalmost preserved portion. The dorsal curvature of the cranial margin is well marked, resulting in a cranially projected, acute craniodorsal corner of the blade. As such, the blade gradually expands dorsally, especially within its dorsal third, the medial surface of which is covered with longitudinal striations. The long axis of the blade is inclined slightly cranially, forming an angle of c. 70° relative to the scapular body, as seen in one specimen of *Sa*. *tupiniquim* [29]. The dorsal margin is not complete, lacking its caudal corner, but can be estimated to be approximately three times craniocaudally broader than the minimal breath of the blade, which is located within its ventral third. It is not clear, however, if the dorsal margin is perpendicular to the long axis of the blade or cranioventrally canted. A longitudinal ridge extends along the ventral half of the medial surface of the blade, bound cranially and caudally by equally elongated depressions, possibly for the origins of m. subscapularis and m. scapulohumeralis caudalis, respectively [29]. The ridge is closer to the caudal margin of the bone, better developed at its ventral portion, and extends ventrally towards the caudal part of the scapular body. The scapular body is laminate cranially and, due in part to the aforementioned ridge, lateromedially broader at its caudal portion. The dorsal margin of the acromion is set at an angle slightly lower than 90° to the main axis of the blade, forming a continuous curve towards its cranial margin. On the caudal portion of the bone, the line extending from the dorsal lip of the glenoid to the caudoventral margin of the blade is slightly concave, bearing a poorly developed tuber for the origin of the scapular head of M. triceps [29]. Cranioventral to that, and caudal to the ventral extension of the medial ridge of the blade, the medial surface of the scapular body is slightly concave. The ventral margin of the scapula is slightly sigmoid, convex at its caudal portion and concave cranially. The adjacent medial surface is heavily striated. A circular pit located above its cranial portion of that margin represents a breakage, with no relation to the coracoid foramen. The glenoid is caudoventrally and slightly laterally oriented, forming an angle of about 60° (in lateral/medial view) relative to the main axis of the ventral margin. The caudal outline of the glenoid is more than twice as long dorsoventrally than it is lateromedially broad at the ventral articulation with the coracoid, but this might in part be due to taphonomic compression. From its flat ventral margin, the glenoid extends dorsolaterally, narrowing towards its rounded dorsal margin.

**Fig 21:**
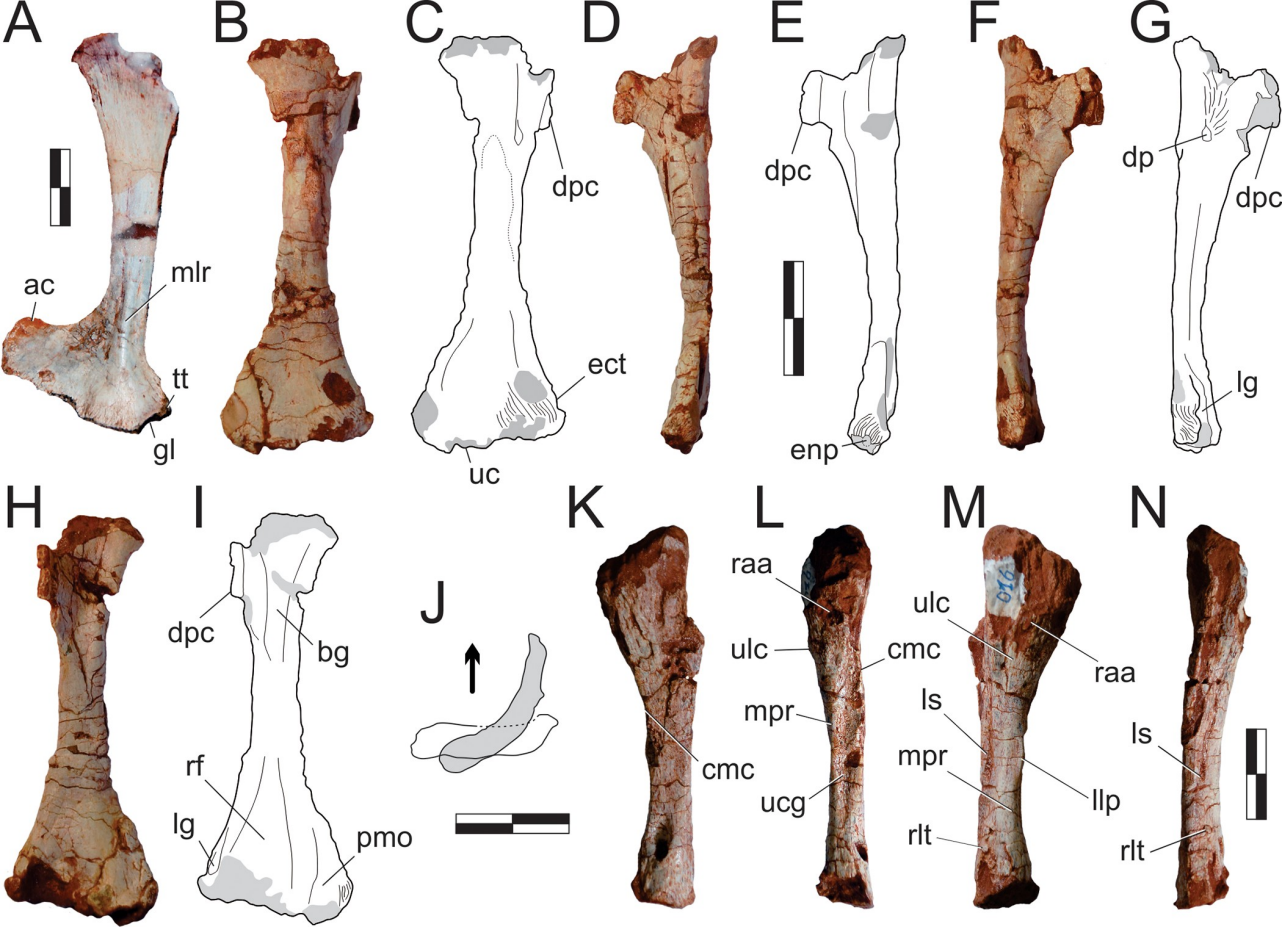
*Pampadromaeus barberenai* (ULBRA-PVT016), pectoral skeleton. Photographs (A-B,D,F,H,K-N) and drawings (C,E,G,I-J) of the right pectoral elements. A, Scapula in medial view. Humerus in medial (D-E), caudal (B-C), lateral (F-G), and cranial (H-I) views; J, relative position of proximal (in grey) and distal ends (arrow points cranially). Ulna in medial (K), cranial (L), lateral (M), and caudal (N) views. Abbreviations: ac, acromion; bg, biceps gutter; cmc, ulnar craniomedial corner; dp, deltoid pit; dpc, deltopectoral crest; ect, ectepicondyle; enp, entepicondyle pit; gl, glenoid; lg, ectepicondyle ligament groove; llp, lip-like projection; ls, lateral sulcus; mlr, medial longitudinal ridge; mpr, medial process ridge; rf, radial fossa; pmo, pronator muscles origin; raa, radial articulation area; rlt, radial ligament tubercle; tt, triceps tuber, uc, ulnar condyle; ucg, ulnar cranial groove; ulc, ulnar lateral crest. Sediment filled areas in grey. Except in J, grey areas indicate broken surfaces. Scale bars = 2 cm.

#### Humerus (Fig 21B–21J)

The right humerus is found isolated on the edge of the block during excavation. It lacks most of its proximal portion, precluding a precise assessment of the total length of the bone. However, a rough estimate yields a length of about 85 mm. The bone is lateromedially expanded in both the proximal and distal portions, the distal end being 27 mm broad lateromedially. Scaling this distal breadth based on those of the humeri of *E*. *lunensis* [34] and *B*. *schultzi* (ULBRA-PVT280) results in a humerus length for *Pam*. *barberenai* that is unrealistically long compared to what is in fact preserved of the bone. Therefore, we interpret the distal end of the humerus of *Pam*. *barberenai* to have been significantly transversely expanded relative to those of *E*. *lunensis* and *B*. *schultzi*, more closely matching the proportions seen in *Sa*. *tupiniquim* [29]. However, extrapolating the total length of the humerus from the distance between the distal end of the bone and the approximate apex (also incomplete) of the deltopectoral crest yields an estimated relative size for that crest closer to that of *E*. *lunensis* and *B*. *schultzi*, which is somewhat shorter than that of *Sa*. *tupiniquim*. The proximal portion of the humerus preserves most of the distal part of the deltopectoral crest (but not its very tip), which expands from the lateral margin of the bone, forming a roughly 65° angle to the transverse intercondylar axis. The crest forms a 45° angle relative to the long axis of the preserver portion of the expanded proximal part of the bone, which is rotated about 20° contraclockwise (as seen from a proximal viewpoint in the right bone) to the intercondylar line. Medial to the deltopectoral crest, the cranial surface of the humerus is excavated by the “biceps gutter” [29]. The lateral surface of the crest bears a subtriangular rugose area for the insertion of m. latissimus dorsi, distal to which there is a well-developed deltoid pit [29]. The preserved cranial margin of the deltopectoral crest, extending proximally from its broken tip, is thickened and slightly convex (cranially and medially) with a medially rugose area for the insertion of m. supracoracoideus. Distal to the lost tip, a sharp cranially concave segment extends distally, merging smoothly with the shaft. The preserved segments of the deltopectoral crest proximal and distal to the tip form an angle of about 120° to one another in lateral/medial view. The proximal part of the humerus is slightly craniolaterally bowed, whereas the shaft is continuously bowed caudally until the distal end. A such, the main axis of the bone is sigmoid in lateral/medial views, but virtually straight in cranial/caudal views. The shaft is ovoid in cross section, with its main axis lateromedially elongated. The distal end of the humerus expands lateromedially into well-developed epicondyles, with the entepicondyle much more expanded than the ectepicondyle. The entepicondyle bears a well-developed and rugose caudomedial border that surrounds a craniomedial pit (= pit “number 1” in *Sa*. *tupiniquim* [29]). A smooth, concave area is visible on the cranial surface of the entepicondyle (“pmo” in Fig 21I), possibly for the origin of pronator muscles [29]. The centre of the cranial surface of the distal humerus is occupied by a not very strongly marked radial fossa ([34]; = cuboid fossa. = fossa m. brachialis [29]). The ectepicondyle is more poorly preserved, but has a proximally extending lateral ridge and a proximodistally elongated ligament groove craniomedial to that [29]. The caudal surface of the ectepicondyle is rugose for the origin of mm. extensor carpi ulnaris and extensor digitorum communis [29]. The distal expansion of the humerus encompasses the radial and ulnar condyles, the latter extending slightly more distally than the former. Both are also expanded craniocaudally, but further details of the articulations themselves are lacking due to the poor preservation of the distal margin of the bone.

#### Ulna (Fig 21K and 21N)

Along with the humerus, a right ulna was recovered isolated on the perimeter of the block during its excavation. It is nearly the same length as the preserved part of the humerus and relatively more robust, extrapolating the expected size for an associated antebrachial element. Nevertheless, its anatomy matches what would be expected for an early dinosaur ulna and there is no clear evidence of other taxa in the sample. Therefore, that ulna is here described as tentatively belonging to the holotype of *Pam*. *barberenai*. Its shaft is relatively well preserved, but the proximal and distal articulations have been badly eroded. Overall, the ulna is medially bowed, with a straight medial margin and a concave lateral margin. Similarly, the caudal margin is also mostly straight, but the cranial is markedly concave. This shape is produced by the transverse and especially craniocaudal expansion of both distal and, especially, proximal portions of the bone. In lateral/medial views, the proximal articulation forms a c. 45° angle to the long axis of the shaft, whereas that angle is closer to a right angle for the distal articulation. As preserved, the olecranon process accounts for slightly more than 10% of the entire length of the ulna, but most of its bone surface is lost. Its size and rounded outline suggest that the olecranon of *Pam*. *barberenai* is not as proximally expanded as in *Sa*. *tupiniquim* [29] and possibly *C*. *novasi* ([31]; but see Martínez *et al*. [32]). The proximal part of the ulna is laterally expanded in the area of the “lateral process” [29], but the process itself is broken. This produces a subtriangular cross-section to this part of the bone, with mostly flat surfaces facing medially, craniolaterally, and caudolaterally. The crest extending from the area of the “lateral process” (“lateral crest” of Langer *et al*. [29]) shallowers as it proceeds distally. It laterally bounds a subtriangular, craniolaterally facing flat surface that is in turn bound craniomedially by the ridge emanating from the “medial process” (“mpr” in Fig 21L and 21M). This surface corresponds to the radial articulation area. The mid-shaft of the ulna is also subtriangular in cross-section, but with distinct medial, caudolateral, and cranial surfaces. At mid-shaft, the caudolateral surface has a proximodistally oriented shallow sulcus (“ls” in Fig 21M), which is strongly scarred. These scars expand distally, almost reaching the distalmost caudal portion of the shaft. The lateral margin of the distal half of the ulna is formed by a ridge that extends distally from the area of the “medial process” [29], forming a sharp craniolateral corner. This forms the lateral margin of the longitudinal groove that occupies most of the cranial surface of the ulnar shaft and might represent the origin area of m. pronator quadratus [29]. The groove is deeper at mid-shaft and bound medially by a ridge that forms the craniomedial corner of the bone. Both ridges extend distally towards the respective corners at the distal end of the ulna, merging with the shaft before the distal quarter of the bone. The craniolateral ridge forms a lip-like cranial projection at mid-shaft that, according to Sereno [81], “may represent the attachment site of an interosseous membrane between the radius and ulna”. The medial surface of the ulna is flat, but heavily striated in the proximal third. Details of the distal articulation surface are difficult to discern, but its caudal portion is flat, whereas the cranial surface is relatively concave. A small rounded tubercle is present on the lateral surface of the distal end of the ulna, which is possibly related to radial ligaments.

### Pelvic girdle and limb

Preserved elements of the pelvic skeleton include partial ilia, the proximalmost portion of the left ischium, partially preserved femora, very incomplete tibiae, nearly complete fibulae, various metatarsals, and possibly some pedal phalanges. No tarsal bones were recovered. The relative length of the limb elements is hard to precisely determine. The best-preserved right fibula is 150 mm long, whereas the left is 145 mm as preserved. In contrast, the nearly complete right femur is c. 130 mm long as preserved, suggesting that the bone is substantially shorter than the epipodium. However, both femora are preserved perpendicular to the bedding plane of the rock and probably suffered significant compression along their longitudinal axis. This is inferred with reference to the proximal flattening of the head and abundant shearing fractures along the shaft. Similar deformations are seen in both tibiae, which were collected with no orientation data from the perimeter of the block. The left tibia is c. 125 mm long as preserved, although parts of the proximal and distal ends are missing. In *Sa*. *tupiniquim*, the femoral length is twice that of the dorsal iliac blade. Presuming a similar ratio, this results in a femur nearly 16 cm long for *Pam*. *barberenai*. Based on these measurements/comparisons, it remains uncertain if *Pam*. *barberenai* had an epipodium significantly longer than the femur. Nonetheless a great deal of longitudinal compression would be required to reduce both femora from close to the length of the fibulae to 85% of their original length.

#### Ilium (Fig 22A–22H)

Both ilia are partially preserved, the left lacking most of the postacetabular ala, and the right lacking the ischial peduncle and the caudal part of the acetabulum. The right ilium was recovered isolated on the perimeter of the block. It is still imbedded in the matrix and exposed only in medial view. The left element was prepared out of the main block and is exposed in all views. The dorsal blade of the ilium is deep, representing more than 60% of the total dorsoventral depth of the bone at the acetabular area. Its dorsal margin is convex cranially and straight caudally in lateral view. In dorsal view, the blade is mostly straight, but medially convex at its cranial fourth, as its tip turns laterally. The cranial tip of the preacetabular ala does not reach the level of the cranial margin of the pubic peduncle. In lateral view, the preacetabular ala of the left ilium is more pointed (subtriangular) relative to its right counterpart, which is more rounded. The craniolateral surface of the ala is scarred for muscle attachment, probably M. iliotibialis cranialis [28]. The iliac preacetabular ridge [28] is subtle, forming a cranially concave crescentic ridge that extends from the lateral surface of the bone, craniodorsal to the acetabulum, to join the ventral margin of the preacetabular ala. Medial to that, the sharp dorsal edge of the pubic peduncle extends as a ridge onto the medial surface of the ilium. A groove (“preacetabular fossa” of Langer *et al*. [10]; “medial fossa” of Sereno *et al*. [34]) separates the two ridges, twisting from the lateral (ventrally) to the medial surface (dorsally) of the ilium. The remainder of the lateral surface of the iliac blade, as preserved in the left element, is smooth and subtly concave, with only a laterally raised caudoventral portion dorsal to the cranial edge of the brevis shelf.

**Fig 22:**
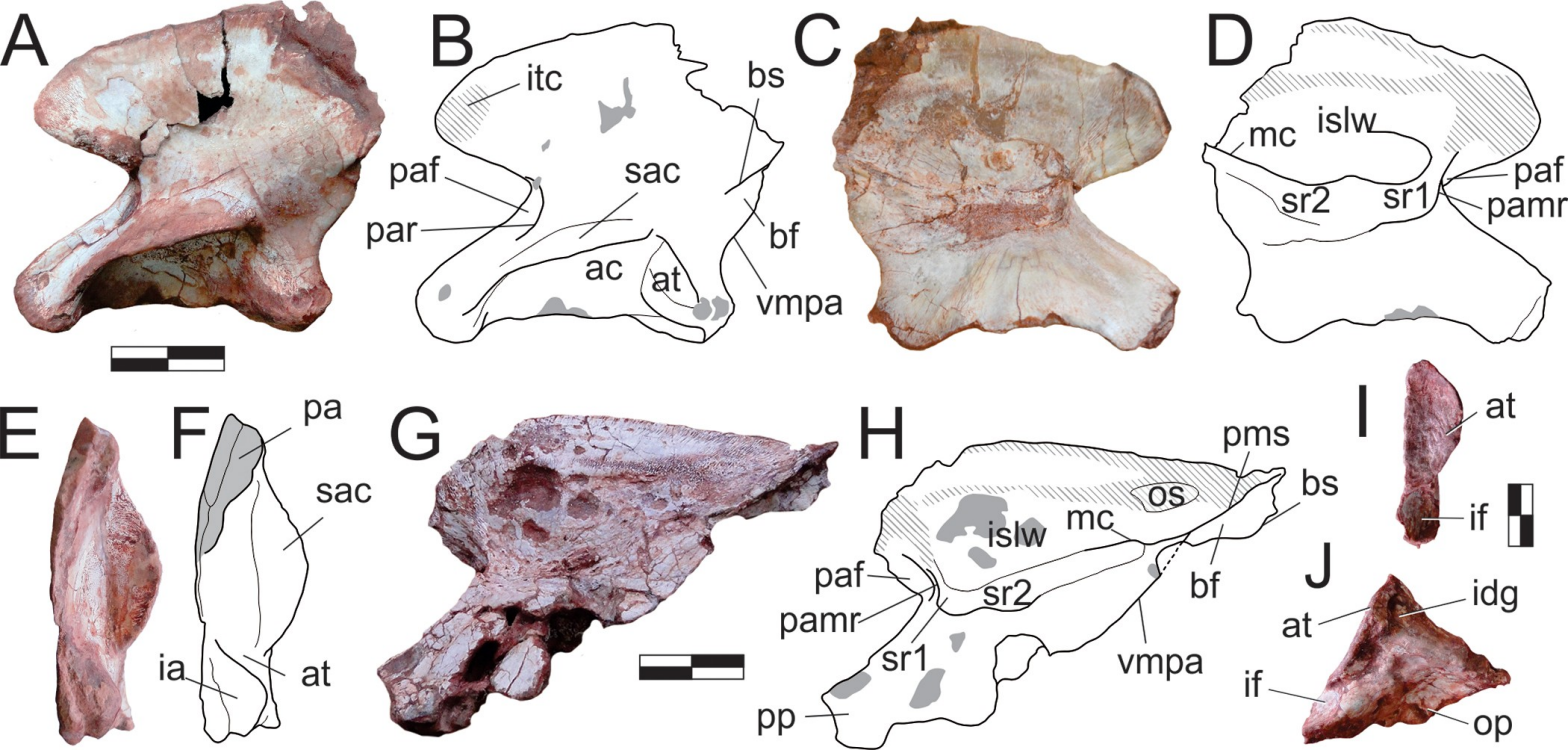
Pampadromaeus barberenai (ULBRA-PVT016), pelvic girdle. Photographs (A,C,E,G,I-J) and drawings (B,D,F,H) of the pelvic elements. Left ilium in (A-B) lateral, (C-D) medial, and (E-F) ventral views. Right ilium in (G-H) medial views. Left ischium in (I) proximal and (J) lateral views. Abbreviations: ac, acetabulum; at, antitrochanter; bf, brevis fossa; bs, brevis shelf; ia, ischial articulation; idg, ischial dorsal groove; if, ischial fossa; itc, M. iliotibialis cranialis scar; islw, lateral wall of the intercostal space; mc, medial crest; op, obturator plate; os, second sacral transverse process attachment area; pa, pubic articulation; paf, preacetabular fossa; par, iliac preacetabular ridge; pamr, iliac preacetabular medial ridge, pms, posteromedial shelf; pp, pubic peduncle; sac, supracetabular crest; sr, sacral rib articulations; vmpa, ventral margin of the postacetabular ala. Grey areas indicate parts broken or covered by sediment; hatched indicates muscle insertions areas. Scale bar = 2 cm (A-H); 1 cm (I-J).

The medial surface of the iliac blade is heavily scarred for the attachment of the sacral ribs and transverse processes. Its slightly depressed central part is smoother, forming the lateral wall of the sacral intercostal space. Such depressed area is subtriangular in outline, with cranial, dorsal, and caudoventral margins (Fig 22H). The former corresponds to the subvertical ridge (“pamr” in Fig 22D and 22H) which extends dorsally from the dorsal edge of the pubic peduncle, gradually flattening on the medial iliac surface, the caudal margin of which is excavated to receive the vertical (cranial) component of the first primordial sacral rib. Both the cranial and dorsal margins of the subtriangular depression are surrounded by striations related to ligaments attaching the ilium to the sacrum, which are continuous with similar striations extending to the dorsal margin of the ilium. The horizontal (caudal) component of the first primordial sacral rib forms a broad, dorsally concave crescentic articular facet which continues cranially and caudally to merge the articular facets of the vertical wall and second primordial sacral rib, respectively. The latter articular facet is also dorsally concave, with a ventral margin that is continuously straight with that of the first sacral rib, forming the caudal part of the main horizontal rib articulation of the medial surface of the ilium. Compared to the facet for the first sacral rib, the facet for the second sacral rib is dorsoventrally broader at its cranial end, but its caudal part tapers dorsocaudally. With the exception of the inconspicuous ventral margin of the second sacral rib articulation, the rib attachments all have sharp, lip-like borders on the medial surface of the ilium. The dorsal margin of the second sacral rib attachment forms an especially acute crest (“mc” in Fig 22) that extends caudally to merge with the ventral margin of the postacetabular ala, forming the medially expanding, sub horizontal “posteromedial lamina/shelf” ([31]; = “medial lamina/blade” of Martínez & Alcober [30]). Dorsal to this shelf, a scarred ovoid area (“os” in Fig 22H) represents the attachment point of the transverse process of the second sacral vertebra. The “posteromedial lamina” forms the medial margin of the transversely flaring caudal portion of the brevis fossa. In medial view of the right ilium, the more vertically oriented brevis shelf (“iliac spine” of Martínez & Alcober [30]) extends along the ventral length of the postacetabular ala, projecting further ventrally than the “posteromedial shelf”. The caudal tip of the “posteromedial shelf” projects further dorsally than the dorsal margin of the iliac blade, so that the dorsal profile of the ilium is slightly concave at its caudal end, although the dorsal margin of the blade itself remains virtually straight. The caudal margin of the postacetabular ala is concave in dorsal/ventral views, with the projecting tips of the brevis and posteromedial shelfs framing a caudal recess. In medial view the caudal margin of the postacetabular ala is sub-square shaped, with a rounded caudoventral corner. Only the cranial-most portion of the brevis shelf/fossa is visible in lateral view of the left ilium. The portion of the fossa preserved in that element is lateromedially narrow, whereas the shelf is subtle and positioned dorsal to the ventral margin of the postacetabular ala. There is no evidence for the attachment of a caudosacral vertebra on the caudomedial surface of the postacetabular ala.

The acetabulum of *Pam*. *barberenai* is substantially craniocaudally longer across its ventral edge than dorsoventrally deep at its caudal margin (c. 30 *vs* 20 mm). As its dorsal and cranial margins are cranioventrally to caudodorsally oriented, the acetabulum gets narrower both cranially and dorsally. Its medial wall is laterally concave, with the deepest inflection point projecting more than five millimetres medial to the cranial and caudal margins. The ventral margin of the acetabular wall is subtly concave in lateral/medial views, with an inflection of less than five millimetres from the line connecting the ventral margins of the public and ischial peduncles. This strictly configures a semi perforate acetabulum, albeit much less perforate than the condition inferred for *E*. *lunensis* [34]. The supracetabular crest expands over the dorsal and cranial margins of the acetabulum. As such, it is separated into two distinct planes, which meet at an angle of roughly 150°. The maximum lateral projection of the crest occurs at its craniocaudal mid-length, i.e. within the cranial half of the dorsal acetabular margin. It expands ventrally about 8 mm from the dorsal acetabular roof, slight overhanging the acetabulum laterally. The crest extends caudally, abruptly terminating at the caudal margin of the acetabulum. This takes place directly above a dorsocaudal extension of the acetabulum that separates the supracetabular crest from the antitrochanter area. The cranial portion of the supracetabular crest merges smoothly onto the lateral surface of the pubic peduncle, extending along most of its length, but terminating prior to its cranioventral edge. The cranial-most point of the crest is positioned cranial to the cranial margin of the acetabulum. The pubic peduncle extends cranioventrally from the centre of the ilium with a sharp dorsal margin. The peduncle expands in all dimensions as it moves distally, this being especially pronounced craniocaudally. This produces a subtriangular pubic articulation (the distal surface of which is mostly eroded), with the long axis perpendicular to the transverse one. The caudal margin of the pubic peduncle merges abruptly with the acetabular wall. The ischial peduncle is preserved only in the left ilium. Its long axis is oriented almost vertically in lateral view, with a slight caudal deflection at its distal end. As a result, the postacetabular embayment projects further cranially than the caudal tip of the peduncle. The lateral surface of the ischial peduncle merges more smoothly into the acetabular region than that of the pubic peduncle. That surface is, in fact, comprised of two nearly flat subtriangular portions. One is caudolaterally facing and extends from the area between the brevis fossa and the caudal margin of the supracetabular crest towards the caudal half of the distal margin of the peduncle. The other faces craniolaterally and extends from the cranial half of the distal margin of the peduncle towards a point ventral to the caudal tip of the supracetabular crest. It enters the acetabular depression, but bulges laterally to form a not much differentiated antitrochanter. The ischial articulation has a subtriangular outline, with medial, craniolateral, and caudolateral margins, the latter two representing the distal expressions of the two flat surfaces mentioned above.

#### Ischium (Fig 22I and 22J)

Only the proximal-most portion of the left ischium is preserved. It is composed of a robust dorsal portion and the thin obturator plate and has a mostly concave medial surface. Its proximal margin is mostly occupied by the large, lateromedially expanded and slightly concave antitrochanter–the proximal outline of which is medially flat and laterally rounded. The iliac articulation facet is reduced, occupying only the mediodorsal corner of the proximal surface, as in *Sa*. *tupiniquim* [28]. A lateral lip extends along the dorsal third of the antritrochanter, which is continuous with a ridge extending distally on the lateral surface of the bone. Two elongated depressions are visible dorsal and ventral to that ridge, the former of which (“idg” in Fig 22J) is continuous to the typically flat dorsal ischial surface of early dinosaurs, whereas the latter probably housed the origin of adductor muscles [10]. A dorsally raised ridge marks its medial limit, as well as the dorsomedial edge of the ischium itself. On the lateral surface of the bone, a subtler ridge bounds the ventral depression caudoventrally and reaches the ventral margin of pubic articulation area. Ventral to the iliac articulation, the “pubic peduncle” is incomplete, but was probably craniocaudally short and is laterally excavated by a deep fossa (“if” in Fig 22J). This separates pubic articulation from the antritrochanter, forming the acetabular incisure with the latter [28], but does not perforate medially. A similar fossa in *Sa*. *tupiniquim* [28] was misidentified as the ischial articular facet for the pubis. The incomplete obturator plate has a sigmoid ventral margin as preserved. Its medial surface is concave opposite to the aforementioned fossa, whereas its lateral surface is concave caudal to that.

#### Femur (Figs 23 and 24)

**Fig 23:**
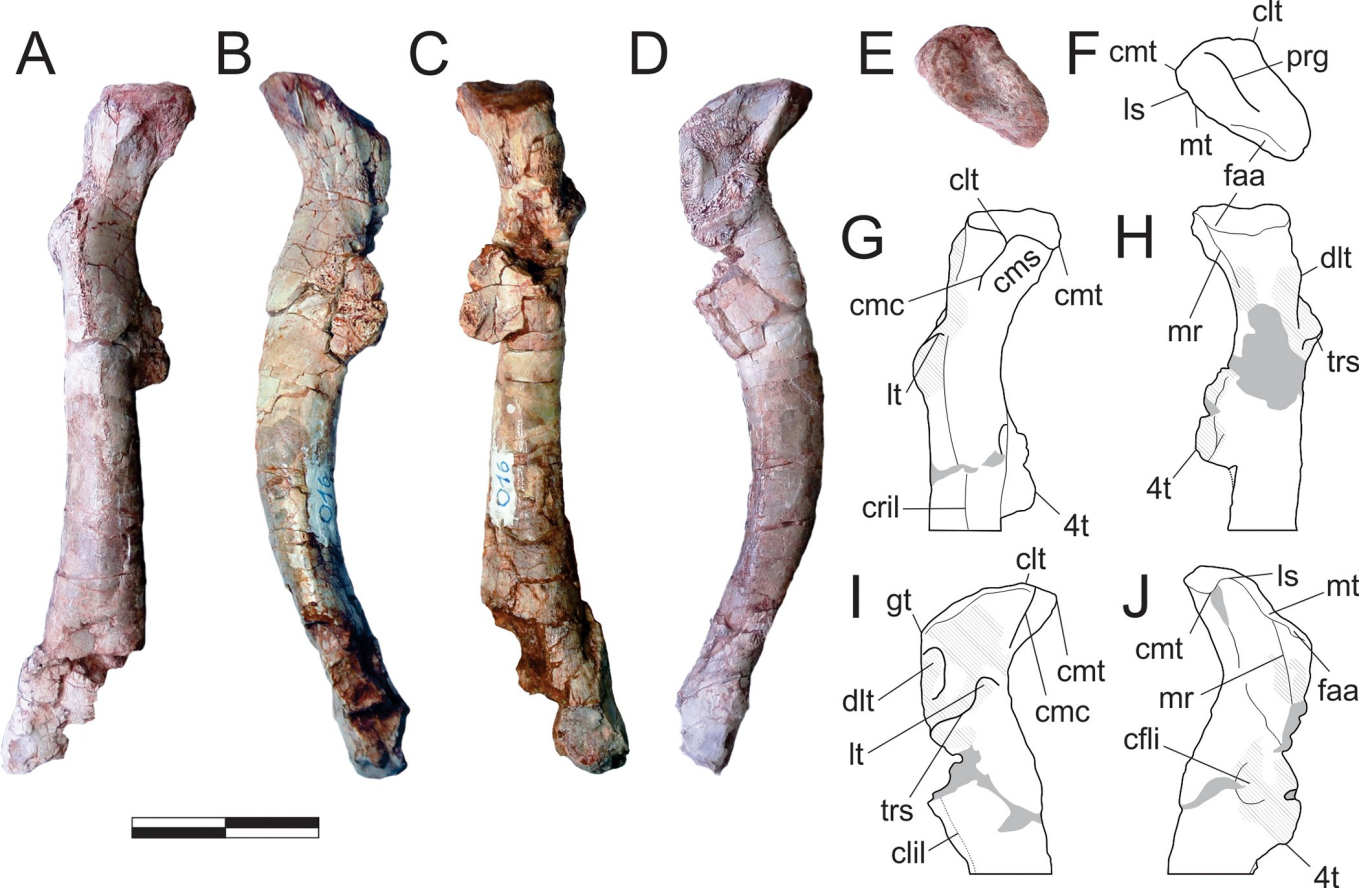
Pampadromaeus barberenai (ULBRA-PVT016), femur. Photographs (A-E) and drawings (F-J) of the right femur in (A,G) cranial, (B,J) medial, (D,H) caudal, (D,I) lateral, and (E-F) proximal views. Abbreviations: 4t, fourth trochanter; cfli, caudofemoralis longus insertion; clil, caudolateral intermuscular line; clt, craniolateral tuber; cmc, craniomedial crest; cms, craniomedial surface; cmt, craniomedial tuber; cril, cranial intermuscular line; dlt, dorsolateral trochanter; faa, facies articularis antitrochanterica; gt, greater trochanter; ls, ligament sulcus; lt, lesser trochantrer; mr, medial ridge; mt, medial tuber; prg, proximal groove; trs, trochanteric shelf. Grey areas indicate parts broken or covered by sediment; hatched areas indicate muscle insertions. Scale bar = 3 cm.

**Fig 24:**
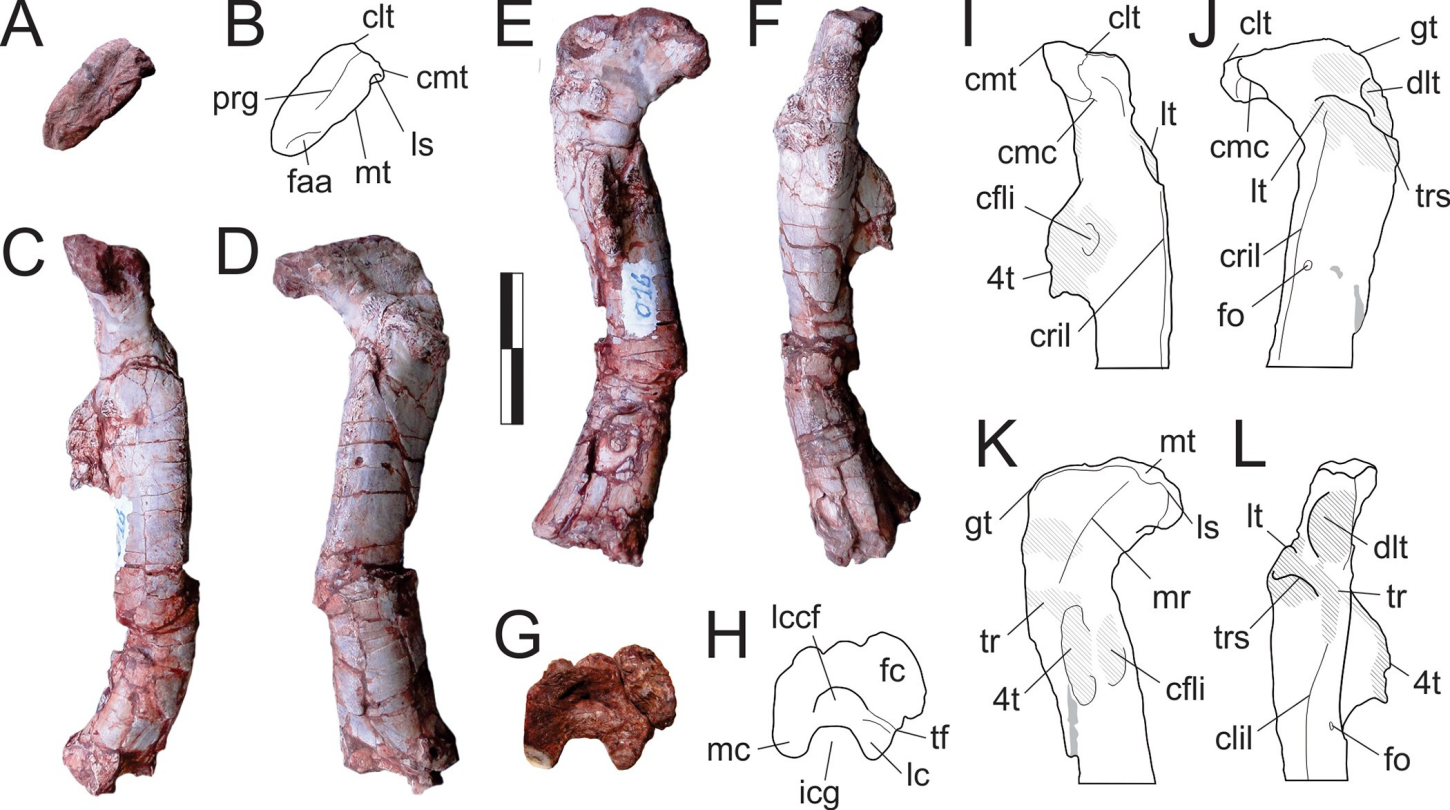
*Pampadromaeus barberenai* (ULBRA-PVT016), femur. Photographs (A,C-G) and drawings (B,H-L) of the left femur in (A-B) proximal, (C,I) craniomedial, (D,J) craniolateral, (E,K) caudomedial, (F,L) caudolateral, and (G-H) distal views. Abbreviations: as in Fig 23, plus fc, fibular condyle; fo, foramen; icg, intercondylar groove; lc, lateral condyle; lccf, facet for Lig. cruciatum craniale; mc, medial condyle; tf, trochlea fibularis; tr, transverse ridge. Grey areas indicate parts broken or covered by sediment; hatched areas indicate muscle insertions. Scale bar = 3 cm.

Both femora are incompletely preserved. The right femur (Fig 23) is missing much of its distal end, with the medial surface particularly badly eroded. The left femur (Fig 24) is represented by its proximal four-fifths (estimated based on its right counterpart) and the isolated distal articulation. The femoral heads of both elements have been distorted due to taphonomic compression, but their proximal surfaces are well preserved. The femoral shaft of the better-preserved right element is cranially bowed in lateral/medial view and straight to slightly medially bowed in cranial/caudal views for its entire length. The femoral head is oriented craniomedially, with its long axis forming an angle of 40° relative to the intercondylar line. The long axis is roughly twice the transverse breadth of the head. The proximal outline of the head is subtriangular, formed of craniomedial, craniolateral, and caudomedial margins, the former of which is the shortest. The former two margins meet at a c. 110° angle, forming the cranially oriented “craniolateral tuber” [4]. The craniomedial and caudomedial margins meet at a c. 90° angle, forming a rounded, medially oriented “craniomedial tuber” ([82], = “caudal lip” of Langer & Ferigolo [4]). The caudomedial margin is marked towards its medial end by the “ligament sulcus” and towards its lateral end by the facies articularis antitrochanterica [28], with both features separated by the “medial tuber” ([4]; = “posteromedial tuber” of Nesbitt [82]; = “posterior tubercle” of Yates [83]). The facies articularis antitrochanterica is not as clearly observable in the left femur, but this may be an artefact of the proximal compression suffered by the bone. A well-developed and sigmoid “proximal groove” (= “mediolateral dorsal sulcus” of Bittencourt & Kellner [84]) extends along the long axis of the proximal articulation. Despite deformation in the femoral heads, some aspects of the shape can be inferred from both preserved femora. In craniolateral/caudomedial views (i.e. those perpendicular to the long axis of the head) the element usually termed the “greater trochanter” (i.e. the corner between the proximal and caudolateral surfaced of the head) is angular (c. 120°). The ventral surface of the femoral head is mainly straight, sloping gradually towards the femoral shaft at an angle of about 140°. In the left element, the medial tip of the head is ventrally projected; i.e. hook-shaped, although this might have been exaggerated by the longitudinal compression suffered by the bone.

The constituent surfaces of the femoral head are marked by a series of muscle related structures, a detailed craniolateral view of which is given in the supplementary material of Cabreira *et al*. [14]. A pair of sharp ridges extend distally from the “craniolateral tuber” (“craniomedial crest” of Cabreira *et al*. [4]) and “craniomedial tuber”, bounding a flat craniomedial surface (“cms” in Fig 23G) between them. Lateral to the more caudal of those ridges, a groove extends distally from the “ligament sulcus” along the caudomedial surface of the femoral head. Lateral to that, an oblique ridge (“medial ridge” of Langer & Ferigolo [4]) extends distally from the “medial tuber”, reaching a rugose area (= anterior portion of the dorsolateral trochanter of Griffin & Nesbitt [85]), which extends laterally towards the dorsolateral trochanter. The distal portion of the craniolateral surface of the femoral head is bordered by rugose structures: the dorsolateral trochanter caudally, the trochanteric shelf distally, and the lesser trochanter cranially. It is mainly smooth between and proximal to those, but its central area is also scared, i.e. “dorsolateral ossification” [86]. The dorsolateral trochanter does not reach the proximal articular surface of the femur to form the “greater trochanter”. It is positioned on the caudolateral surface of the femoral head, forming a thick, cranially expanded eminence. The lesser trochanter is present as a rugose proximal extension of the trochanteric shelf and is not separated from the femoral shaft by a cleft. The trochanteric shelf is well expanded craniolaterally, with a breadth (how much it projects from the shaft) corresponding to more than one-third of the long axis of the femoral head. The lateral end of the trochanteric shelf curves distally towards the caudolateral intermuscular line, with a resulting sigmoidal profile for the entire lesser trochanter plus trochanteric shelf complex. As reported for *E*. *lunensis* [34], a rugose ridge, possibly representing the origin of m. ischiofemoralis [28], diverges from the trochanteric shelf and extends laterodistally to join the proximal margin of the fourth trochanter. From the area of the lesser trochanter, a proximally rugose cranial intermuscular line extends distally along the femoral shaft. The fourth trochanter projects caudally from the caudomedial side of the shaft, positioned about one-third the femoral length from the proximal margin of the bone. It is a prominent, asymmetrical sharp flange, the proximal end of which merges more smoothly onto the shaft than the distal. The trochanter is better preserved in the left femur, demonstrating a sub-rectangular profile with a slightly pendant distal tip. The medial surface of the fourth trochanter presents the typically rounded scarred area (i.e. a central depression bordered distally and cranially by a curved ridge) for the insertion of m. caudofemoralis longus [28]. At the level of the distal margin of the fourth trochanter, a foramen pierces the cranial surface of the femur immediately lateral to the cranial intermuscular line. Slightly distal to that, the nutritive artery of the proximal femur [28] pierces the caudal surface of the bone, lateral to the fourth trochanter.

The femoral mid-shaft is equally broad lateromedially and craniocaudally, with slightly distinct caudolateral and caudomedial corners formed by the respective intermuscular lines. In the right femur the distal third gradually expands lateromedially towards the distal articulation, where muscle scars are visible on the cranial surface of the bone. This expansion is also evident in the larger piece of the left femur (Fig 24A–24F), the broken distal end of which is nearly twice broader lateromedially than craniocaudally. The distal articulation of the left femur is too poorly preserved to proffer adequate description, so the below pertains to the isolated distal end of the right element (Fig 24G and 24H). The distal femur is slightly broader transversally than craniocaudally and bears well-developed medial, lateral, and fibular condyles, as well as a deeply excavated facet for Lig. cruciatum craniale [28]. The latter extends caudolaterally as a groove that separates the lateral (= crista tibiofibularis) and fibular condyles, reaching the trochlea fibularis [87]. The caudally rounded medial condyle occupies the entire medial surface of the distal femoral articulation. The fibular condyle is much larger than the lateral, expanding both laterally and cranially. As a result, the cranial margin of the distal end faces craniomedially, forming an angle of c. 120° relative to the medial margin. However, the shape and orientation of the distal articulation may partially result from craniomedial deformation, as indicated by fractures along its cranial margin, possibly leading to the exaggerated craniolateral projection of the fibular condyle. The intercondylar groove excavates nearly one-third of the craniocaudal thickness of the distal femoral surface and is as lateromedially broad as the fibular and medial condyles.

#### Tibia (Fig 25A and 25B)

**Fig 25:**
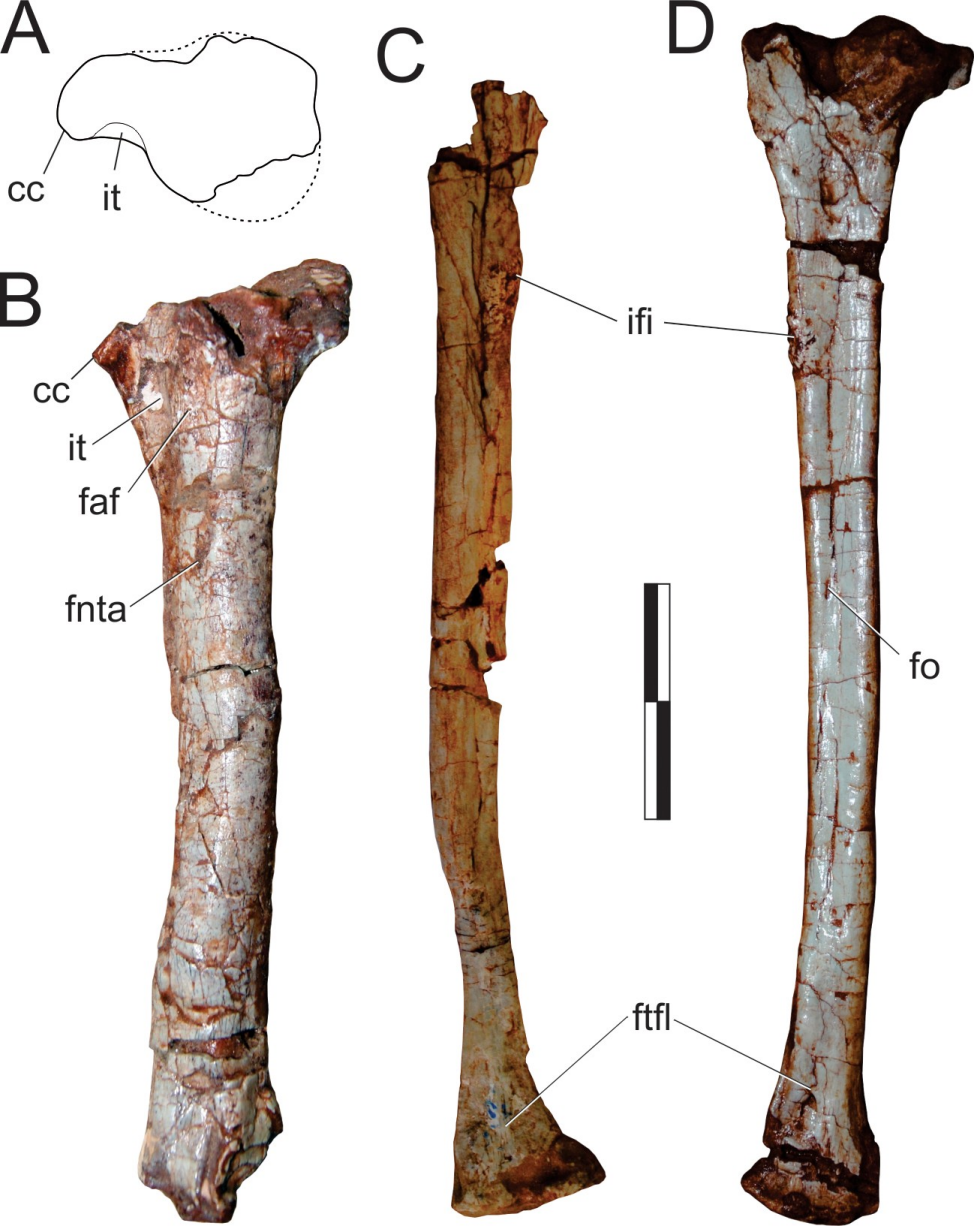
*Pampadromaeus barberenai* (ULBRA-PVT016), tibia and fibula. Drawing (A) and photographs (B-D) of the epipodium. Left tibia in (A) proximal and (B) lateral views. Medial view of the (C) left and (D) right fibulae. Abbreviations: cc, cnemial crest; faf, fibular articular facet; fnta, foramen for the nutritive tibial artery; fo, foramen; ftfl, facet for the tibiofibular ligament; ifi, m. iliofibularis insertion; it, insisura tibialis. Scale bar = 3 cm.

Both tibiae are very incomplete and were recovered isolated on the perimeter of the block. If it was originally of the same length as the fibula, only the proximal 85% of left (Fig 25A and 25B) tibia is preserved, but the proximal articular surface is also missing, and the bone suffered some longitudinal compression. The right tibia is missing its proximal portion until the distalmost extension of the cnemial crest, which is recognized by a cranial expansion of the shaft. It is only slightly shorter than the preserved portion of the left tibia, so that a greater portion of the distal part of the bone must be preserved. The cnemial crest mainly projects cranially, with a slight lateral turn at its cranial tip. The proximal surface of the left tibia is badly preserved, with the fibular condyle missing most of its caudolateral margin. The proximal outline of the bone was reconstructed (Fig 25A) with a maximum transverse breadth of about 70% of its craniocaudal length and a larger fibular condyle, but this is rather uncertain. The proximal portion of the shaft lacks a fibular flange on its lateral surface, but the area caudolateral to the cnemial crest is excavated by the insisura tibialis [88], which is expressed as a craniolateral notch in proximal outline. Caudal to this furrow, the lateral surface bears a rugose rounded bump for the articulation of the fibula and attachment of the tibiofibularis ligament [28]. A foramen, possibly related to the nutritive tibial artery [28, 89–91], is observable distal to the rugose bump. The poorly preserved distal end of the left tibia is not significantly broader lateromedially than craniocaudally, suggesting that any transverse expansion of the bone only occurred distal to that. In contrast, the distalmost preserved portion of the right tibia is lateromedially broader than craniocaudally long and curves slightly cranially. However, this portion of the bone has potentially been affected by taphonomic processes, with a flattened cranial surface and eroded lateral surface. Although most of the distal articulation appears represented in the right tibia, its poor preservation precludes a detailed assessment of its morphology.

#### Fibula (Fig 25C and 25D)

Both fibulae are nearly complete and exposed in medial view. The left element was prepared out of the main block, and lacks its proximal margin, whereas the right is still imbedded in the bearing rock. The fibula is a long, lateromedially flatted (along its entire length) bone that expands craniocaudally at its proximal and distal ends. The former expansion is more marked, especially towards the caudal side. The proximal margin is four times longer (craniocaudally) than broad (lateromedially) and slightly concave medially. The fibular shaft narrows distally, with its distal end about 70% the craniocaudal breath of the proximal end (both excluding the articular expansions). In medial view, a subtle longitudinal ridge extends along the centre of the planar, proximal portion of the bone. On the cranial margin of the fibular shaft, at a distance of roughly 30% of the total length of the bone from its proximal margin, there is a rugose scar for the insertion of m. iliofibularis [90]. The distal third of the fibula is slightly cranially deflected and contraclockwise rotated (as seen from proximal standpoint on the right side), so that the long axes of the proximal and distal margins are not parallel (the latter has a craniomedially to caudolaterally oriented long axis). This is further enhanced by taphonomic deformation in the left fibula. Slightly proximal to the midlength of the bone, a foramen [89] is visible on the cranial part of its medial surface. As in *C*. *novasi* [31], a subtle longitudinal groove extends along most of the medial surface of the fibular shaft. Near the distal end, the craniomedial margin of the fibular shaft is formed by a sharp ridge, which is bounded caudally by an elongated groove for the attachment of the tibiofibular ligament [28]. No proximally expanded “articular facet for the astragalar ascending process” [28] is observable distal to this ridge. The caudal margin of the distal portion of the fibula expands caudal to the distal articular surface, as to form a kind of “posterolateral prominence” [90] (= “posterior tuberosity” of Sereno *et al*. [34]). The distal margin of the bone is slightly everted in medial view.

#### Matatarsus (Fig 26)

**Fig 26:**
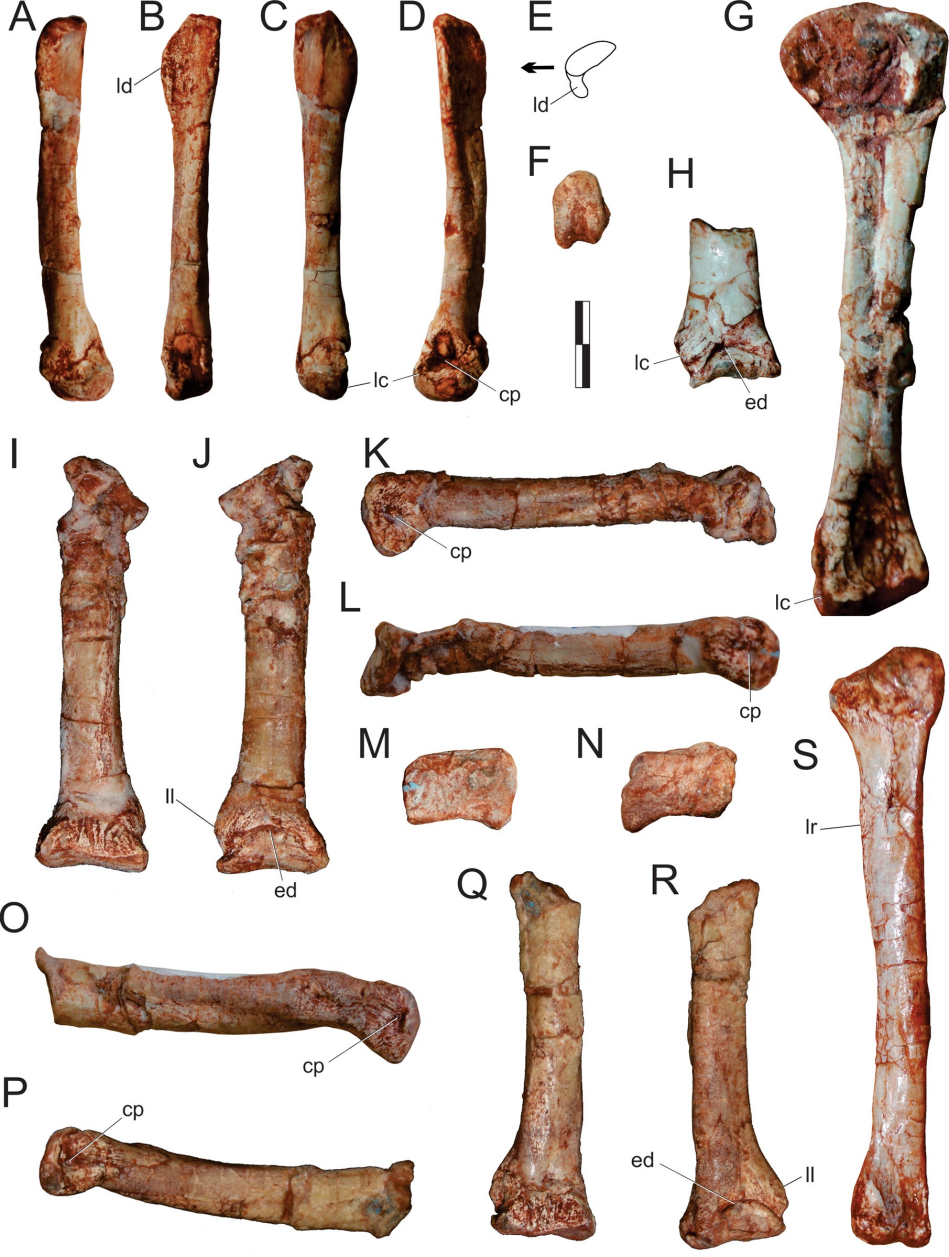
*Pampadromaeus barberenai* (ULBRA-PVT016), metatarsus. Photographs (A-D,F) and drawing (E) of the metatarsus. Left metatarsal I in (A) medial, (B) caudal, (C) cranial, (D) lateral, (E) proximal (arrow points cranially), and (F) distal views. Left metatarsal II in (G) caudal view. Distal portions of right metatarsal II in (H) dorsal view; right metatarsal III in (I) ventral, (J) dorsal, (K) medial, (L) lateral, and (M) distal views; left metatarsal III in (N) distal, (O) medial, (P) lateral, (Q) ventral, and (R) dorsal views. Right metatarsal IV in medial (S) view. Abbreviations: cp, collateral ligament pit; ed, extensor depressions; lc lateral condyle; ld, lateral deflection; ll, lateral lip; lr, longitudinal rugose ridge. Scale bar = 1 cm.

Preserved metapodial elements include complete left metatarsals I and II and the distal half of both metatarsals III, found isolated in the perimeter of the block. In addition, the distal part the right metatarsal II and a complete isolated right metatarsal IV are preserved in the main block. Metatarsal I (Fig 26A and 26F) is 45 mm long. Its proximal end is craniocaudally expanded and laterally flattened to fit the proximomedial margin of metatarsal II. Its cranial margin is lobe-shaped and laterally deflected to cover part of the cranial surface of metatarsal II, producing a craniocaudally concave lateral surface. The cranial and caudal margins of the proximal part of the bone are scarred for ligament insertion. The cranial scar and the lateral concavity extend distally for about two thirds of the entire length of the bone. The distal end of metatarsal I is rotated nearly 50° clockwise (when viewed from a proximal standpoint) relative to the long axis of the proximal surface. The distal articulation is much deeper dorsoventrally than lateromedially broad. The lateral condyle has a well-developed collateral ligament pit, in contrast to the shallow collateral pit present in the medial condyle, and the almost neglectable dorsal extensor depression. In addition to being lateromedially larger, the lateral condyle also expands further distally and dorsally than the medial condyle.

The left metatarsal II (Fig 26G) is 72 mm long, i.e. nearly 50% the length of the epipodium. It is exposed only in caudal view in mini plaster-jacket and crushed craniocaudally. Although the proximal articulation is partially obscured by matrix, it appears lateromedially expanded, with the long axis oblique to that of the distal condyles. The distal end is 13 mm broad and bears a deep lateral collateral ligament pit, whereas the medial surface of the medial condyle is covered by matrix. The caudal surface of the bone is deeply excavated in this area and the lateral condyle expands further distally than the medial. The distal end of the right metatarsal II (Fig 26H) is 12 mm broad and exposed in cranial view. It was misidentified as a phalanx in the upper-left corner of the main block, as depicted in Cabreira et al. [14]. The dorsal surface of the distal end bears a semilunate extensor depression surrounded by rugose areas.

Based on general comparison with the length of metatarsal II, approximately the distal half of both metatarsals III (Fig 26I–26R) are preserved. Their midshaft cross-section is slightly broader lateromedially than craniocaudally and the distal half of the shaft is slightly bowed medially. The dorsal margin of the lateromedially expanded distal articulation is traversed by a distally concave crescentic groove (i.e. extensor depressions), surrounded proximally by a rugose ridge. This ridge expands laterally so as to produce a lateral lip (“ll” in Fig 26) and a squared lateral margin of the ginglymus that extends further proximally than the medial. Thus, the preserved lateral margin of the shaft is more concave than the lateral. In the left element the medial condyle extends slightly more distally than the lateral. The distal condyles have well-developed collateral ligament pits. Both the lateral margin of the lateral condyle and the ventral surface of the distal end bear rugose surfaces. The distal margin is slightly concave in cranial/caudal views. The distal outline is sub-rectangular, about1,5 times broader lateromedially than craniocaudally, with straight lateral, dorsal, and medial margins and a concave ventral margin. The mediocaudal corner is caudally pinched, expanding further caudally than the laterocaudal corner.

The complete right metatarsal IV (Fig 26S) is 70 mm long (about 45% the length of the epipodium). It is still imbedded in sediment, with only the medial surface of the bone exposed. This surface bears a distinct longitudinal rugose ridge (for the ligamentous attachment to metatarsal III) which forms the cranial margin of the proximal third of the shaft. The distal surface is only partially visible, but appears deeper dorsoventrally than lateromedially broad.

#### Phalanges

The pedal digits are represented by only two disarticulated non-ungual phalanges, of which their inferred positions in the foot are tentative. One of them was prepared free, whereas the second is still in the main block, over the mid dorsal portion of the left dentary. These are, respectively, referred to here as phalanx A and B. Both are generally well-preserved, but phalanx A has a plantar-dorsal flattening due to compression. It is longer than phalanx B, measuring 2,6 cm and 1.9 cm each. Phalanx A seems to represent a first phalanx, as its proximal articulation is wider than high, trapezoidal (with the dorsal margin wider than the ventral), and the dorsal intercondylar process weakly developed. Furthermore, the articulation facet is shallowly concave and no vertical ridge separates the medial and lateral facets. There are pits for collateral ligaments in both margins of the distal condyles, the medial of which is much deeper. The extensor pit on the dorsal surface of the distal portion of the bone is well-developed and positioned slightly closer to its lateral margin. The distal ginglymoid articulation has separate condyles, which are rounded and have wider than high outlines in distal view. Also, the medial condyle is more projected distally than the right. Compared with *B*. *schultzi*, the latter feature, coupled to the proximal articulation morphology, suggests that this phalanx represents the first element of the fourth right digit. Phalanx B shares with the former a wider than high proximal articulation, a weakly-developed dorsal intercondylar process, and no ridge dividing the concave proximal articular surface. Accordingly, this morphology suggests that phalanx B also represents a first phalanx, but its proximal surface has a rounded outline. Scars for ligamentous insertions surround the dorsal surface and one of the sides of the proximal portion of the bone. The dorsal surface of its distal portion has a deep extensor pit, and one of the collateral pits is well-developed. The remaining morphology is, however, hidden in the rock matrix.

## Results and discussion of the phylogenetic study

**Fig 27:**
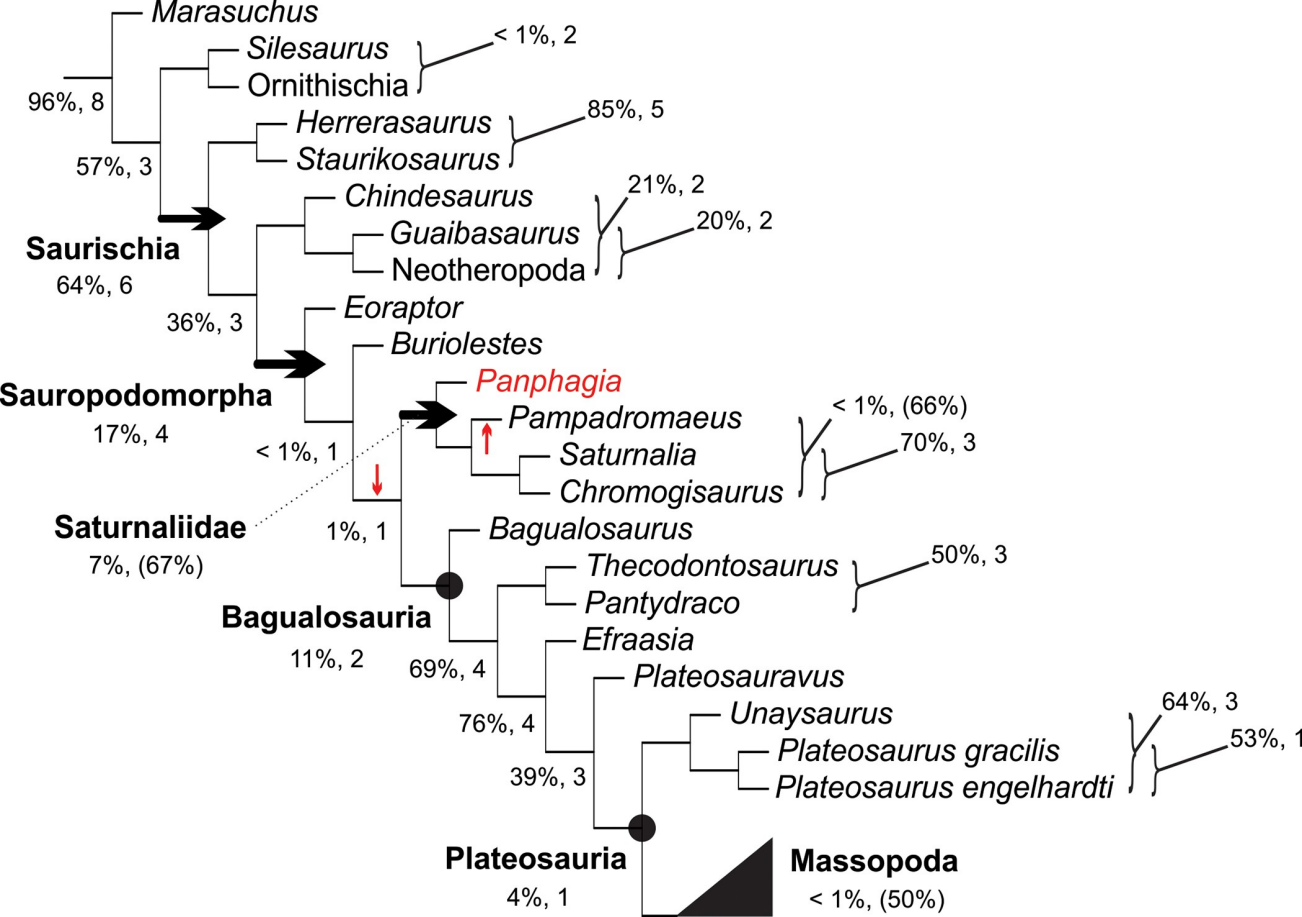
Phylogenetic position of *Pampadromaeus barberenai*. Abridged version of the “majority-rule” consensus of 192 MPTs recovered in the present phylogenetic analysis, depicting the position of *Pam*. *barberenai*. Circles and horizontal-black arrows indicate node- and branch-based clade names, respectively. Vertical-red arrows indicate the alternative positions for *Pan*. *protos* found in a IterPCR analysis. CG bootstrap values are indicated for each clade followed by either “Bremer support” values, for clades present in all MPTs, or by the MPTs percentage (under brackets) in which the clade appears.

An abridged version of the “Majority Rule” consensus (S1 Fig) of the 192 MPTs (1.467 steps each; Consistency Index = 0.325; Retention Index = 0.673) is depicted in Fig 27. CG (Frequency difference) bootstrap values are indicated for each clade, followed by either “Bremer support” values, for clades present in all MPTs, or the MPTs percentage (under brackets) in which the clade appears. Circles and black-horizontal arrows respectively indicate node- and branch-based clades as defined here for Saturnaliidae and Bagualosauria, and following Gauthier [92] for Saurischia, Upchurch [22] for Sauropodomorpha, Sereno [93] for Plateosauria, and Yates [45] for Massopoda.

The resulting phylogenetic hypothesis shows *Ba*. *agudoensis* closer to younger members of the group than to other Carnian sauropodomorphs, as already proposed by Pretto *et al*. [12]. The name Bagualosauria is here erected to join the “minimal clade including *Ba*. *agudoensis* Pretto, Langer & Schultz 2018 [12] and *Saltasaurus loricatus* Bonaparte and Powell 1980 [19]” (new node-based definition). That name refers to the large size of taxa within the clade compared to other Carnian sauropodomorphs [12], a trait that was soon to become typically characteristic of that dinosaur lineage [94–95]. The recovered topology also places *E*. *lunensis* in the sauropodomorph branch, rather than as a theropod as previously proposed, but challenged by most recent studies [3, 5, 35, 96]. Indeed, *E*. *lunensis* was found to represent the sister taxon of all other sauropodomorphs, as opposed to recent studies [5, 35], which placed *Bu*. *schultzi* in such a position. Instead, *Bu*. *schultzi* is here positioned as the sister taxon to a clade composed of Saturnaliidae plus Bagualosauria. Although recovered in all MPTs, the resulting relations of *Bu*. *schultzi* and *E*. *lunensis* to other sauropodomorphs are poorly supported, as indicated by very low Bootstrap and “Bremer support” values (Fig 27). Even less well-supported is the Saturnaliidae clade, a taxon translated from Saturnaliinae [31], with a new branch-based definition (see “Systematic Paleontology” above) to encompass *Sa*. *tupiniquim*, *C*. *novasi*, *Pan*. *protos*, and *Pam*. *barberenai*. However, the nesting of the latter two taxa within Saturnaliidae occurs in only slightly more than 65% of the MPTs. As suggested by an IterPCR analysis [97], this may result from the floating position of *Pan*. *protos*, which was found in other MPTs to alternatively (Fig 27) represent the sister taxon to *Pam*. *barberenai* or to a clade formed by Bagualosauria plus a less inclusive Saturnaliidae (including only *Pam*. *barberenai*, *Sa*. *tupiniquim*, and *C*. *novasi*). Indeed, in all MPTs, *Sa*. *tupiniquim*, *C*. *novasi*, and *Pam*. *barberenai* are closer to one another than any of them are to either *Bu*. *schultzi*, *E*. *lunensis*, or Bagualosauria. Further, our study found relatively good support for the Saturnaliinae clade, composed of *C*. *novasi* and *Sa*. *tupiniquim* only, as previously suggested by many authors [5, 12, 31–32, 35, 56, 98].

After the discovery of *Sa*. *tupiniquim* in 1998 [53], the XXI century witnessed the report of further Carnian sauropodomorphs and the placement of *E*. *lunensis* within the lineage. This resulted in the proposition of two alternative patterns for the phylogenetic arrangement of such taxa. They were either grouped in their own clade of small-bodied forms [3, 31, 94, 98] or arranged in a more pectinate fashion [5, 12, 30, 32, 35, 55–56], with some taxa successively closer to the post-Carnian sauropodomorphs. However, studies recovering the “Carnian clade” were either broader in scope [3, 96], contained a relatively shallow taxonomic sample, and/or were performed prior to the proposed nesting of *E*. *lunensis* within Sauropodomorpha [31, 98]. Instead, most recent studies dealing with early sauropodomorphs and based on the modification of independent datasets ˗ e.g. [12, 32, 55] derived from Yates [45], and [5, 35, 55] derived from Bittencourt *et al*. [99] ˗ agree in a more pectinate pattern. Our phylogenetic hypothesis suggests an intermediate arrangement, with a pectinate early branching with *Bu*. *schultzi* and *E*. *lunensis* and a less inclusive Carnian clade, i.e. Saturnaliidae, sister to Bagualosauria. Similar arrangements, but with taxa in different positions, were already proposed by other authors [35–36, 43]. Regardless of their respective positions, the idea that *Bu*. *schultzi* and *E*. *lunensis* fall outside the clade formed of all other sauropodomorphs is becoming mainstream [5, 12, 32, 35, 56]. Likewise, the presence of a clade of *Saturnalia*-like sauropodomorphs is also likely, although its inclusivity (if it encompasses *Pan*. *protos* and *Pam*. *barberenai* or not) is still unclear.

With seven taxa identified in the last twenty years, the high diversity of sauropodomorphs in the Carnian may have came as a surprise for dinosaur researchers, especially considering: (1) the much lower diversity of theropods and ornithischians at the time; (2) the fact that all those taxa come from only two nearby areas in South America and; (3) they have very similar body-plans (with the exception of *Ba*. *agudoensis*). Thus, one may easily suspect that such high diversity could represent an artificial taxonomic inflation. However, even if sometimes based on arguably minor diagnostic features, the validity of these different species of Carnian sauropodomorph has withstood numerous comparative studies, conducted here and by other authors [3, 5, 14, 30–35]. Moreover, with the obvious exception of *C*. *novasi*, most are known from relatively complete remains, allowing comprehensive anatomical evaluations based on the identification of autapomorphies and/or unique sets of anatomical traits. Nonetheless, when we included all valid Carnian sauropodomorphs within a phylogenetic analysis, evidence for alternative arrangements among these taxa seem to have counterweighted one another. As a result, the signal provided by the sum of their anatomical variations did not allow the establishment of a robust phylogenetic hypothesis of relationships.

Interestingly, the explanation for the nearly equal parsimony of the alternative phylogenetic arrangements proposed for Carnian sauropodomorphs may be the same as to why their diversity is probably not as overstated as one might guess. Given that the group is considerably more diverse than the other major dinosaur clades right through to the end of the Triassic [100], their Carnian diversity may well be understood as early stages of the Late Triassic sauropodomorph adaptive radiation [101]. Whatever the deeper causes of both phenomena, adaptive radiations are frequently coupled with conditions of relaxed selection [102–107], which in turn may facilitate the establishment of “zones of variability” sensu Bever et al. [108], when variation between plesiomorphic and apomorphic states of characters (which ought first to appear as polymorphisms within populations [109]) persist across speciation events. Hence, apomorphies later to be fixed ˗ i.e., “acquire evolutionary burden” [109] ˗ during the evolution of a lineage may intermittently appear among species of its early offshoots. Homoplasy is thus rampant [110–111], making it hard to define the relations among taxa belonging to such “zones of variability”.

In the case of sauropodomorphs, their Carnian increase in species richness occurred in the absence of significant changes in disparity, i.e. with the retention of similar body plans. On the contrary, “disparity first” patterns are apparently more frequent in ‘early burst’ models of adaptive radiation [112], especially in the aftermath of niche openings caused by extinction events [113]. However, the radiation of the typically herbivorous sauropodomorphs started (during Carnian times) with faunivorous taxa [5] within an ecological scenario in which other herbivorous terrestrial tetrapods (dicynodonts, traversodontid cynodonts, rhynchosaurs) were abundant [15, 59]. Dental modifications towards herbivory were by then already present among sauropodomorphs, so that tooth anatomy plays a significant role in differentiating between Carnian members of the group [5]. Moreover, the varied dental anatomy observed in the earliest sauropodomorphs suggests divergent strategies in the initial exploitation of plant resources, most likely resulting in dietary niche partitioning and allowing high species richness with similar body plans in restricted geographical areas, as is today seen among various herbivorous taxa [114–115].

In sum, sauropodomorph evolution in the Carnian may be characterized by the acquisition of herbivorous adaptations, allowing high species richness with low disparity, which in turn hampers the definition of their phylogenetic relationships. That said, this is clearly a hypothetical scenario requiring further scrutiny. Given that an incomplete understanding of the alpha-taxonomy of Carnian sauropodomorphs potentially complicates the reconstruction of their relationships, future attempts at exploring the scenario presented here should be conducted in tandem with rigorous descriptive analyses of their anatomical variation. As such, a more comprehensive investigation of taxa with more than one assigned specimen (e.g. *Sa*. *tupiniquim*, *Bu*. *schultzi*, *E*. *lunensis*) may help to unscramble species diversity from taxonomic inflation.

## Supporting information

S1 Fig Majority-rule consensus of the MPTs resulting from the phylogenetic analysis. (EMF)

S1 File List of 413 characters employed in the phylogenetic analysis. (DOCX)

S2 File Character-taxon matrix. (DOCX)
