# Supplementary material for: Anatomy of the dinosaur Pampadromaeus barberenai (Saurischia—Sauropodomorpha) from the Late Triassic Santa Maria Formation of southern Brazil
Source: PLoS One. 2019 Feb 20;14(2):e0212543. doi: 10.1371/journal.pone.0212543 (PMC6382151; doi:10.1371/journal.pone.0212543)
Supplement: S1 File — (DOCX) [file pone.0212543.s002.DOCX]

**S1 File: List of 413 characters employed in the phylogenetic analysis**

Multistate characters 8, 18, 22, 39, 56, 68, 90, 112, 114, 118, 128, 141, 144, 146, 147, 154, 159, 164, 166, 167, 174, 204, 207, 215, 222, 227, 234, 242, 251, 254, 266, 279, 300, 306, 314, 334, 347, 350, 352, 357, 361, 363, 373, 374, and 410 were ordered.

Characters 1-370 from Bronzati et al. (2017); characters 371-413 (in blue below) added here.

1. Skull to femur ratio: greater than 0.6 (0); less than 0.6 (1). (modified from Gauthier 1986).
2. Lateral plates appressed to the labial side of the premaxillary, maxillary and dentary teeth: absent (0); present (1). (Upchurch, 1995)
3. Relative height of the rostrum at the posterior margin of the naris: more than 0.6 the height of the skull at the middle of the orbit (0); less than 0.6 the height of the skull at the middle of the orbit (1). (Langer 2004)
4. Foramen on the lateral surface of the premaxillary body: absent (0); present (1). (Yates 2007)
5. Distal end of the dorsal premaxillary process: tapered (0); transversely expanded (1). (Sereno 1999)
6. Profile of premaxilla: convex (0); with an inflection at the base of the dorsal process (1). (Upchurch 1995)
7. Size and position of the posterolateral process of premaxilla: large and lateral to the anterior process of the maxilla (0); small and medial to the anterior process of the maxilla (1). (Yates 2007)
8. Relationship between posterolateral process of the premaxilla and the anteroventral process of the nasal: broad sutured contact (0); point contact (1); separated by maxilla (2). (modified from Gauthier 1986)
9. Posteromedial process of the premaxilla: absent (0); present (1) (Rauhut 2003)
10. Shape of the anteromedial process of the maxilla: narrow, elongated and projecting anterior to lateral premaxilla-maxilla suture (0); short, broad and level with lateral premaxilla-maxilla suture (1). (Yates 2007)
11. Development of external narial fossa: absent to weak (0); well-developed with sharp posterior and anteroventral rims (1). (Yates 2007)
12. Development of narial fossa on the anterior ramus of the maxilla: weak and orientated laterally to dorsolaterally (0); well-developed and forming a horizontal shelf (1). (modified from Upchurch 1995)
13. Shape of subnarial foramen: rounded (0); slot-shaped (1). (Yates 2007)
14. Maxillary contribution to the margin of the narial fossa: absent (0); present (1). (Yates 2007)
15. Diameter of external naris: less than 0.5 of the orbital diameter (0); greater than 0.5 of the orbital diameter. (Wilson and Sereno 1998)
16. Shape of the external naris (in adults): rounded (0); subtriangular with an acute posteroventral corner (1). (Galton & Upchurch 2004)
17. Level of the anterior margin of the external naris: anterior to the midlength of the premaxillary body (0); posterior to the midlength of the premaxillary body (1). (Rauhut 2003a)
18. Level of the posterior margin of external naris: anterior to, or level with the premaxillamaxilla suture (0); posterior to the first maxillary alveolus (1); posterior to the midlength of the maxillary tooth row and the anterior margin of the antorbital fenestra (2). (modified from Wilson and Sereno 1998)
19. Dorsal profile of the snout: straight to gently convex (0); with a depression behind the naris (1). (Yates 2007)
20. Elongate median nasal depression: absent (0); present (1). (Sereno 1999)
21. Width of anteroventral process of nasal at its base: less than the width of the anterodorsal process at its base (0); greater than the width of the anterodorsal process at its base (1). (modified from Sereno 1999)
22. Nasal relationship with dorsal margin of antorbital fossa: not contributing to the margin of the antorbital fossa (0); lateral margin overhangs the antorbital fossa and forms its dorsal margin (1); overhang extensive, obscuring the dorsal lachrymal-maxilla contact in lateral view (2). (modified from Sereno 1999)
23. Pointed caudolateral process of the nasal overlapping the lachrymal: absent (0); present (1). (Sereno 1999)
24. Anterior profile of the maxilla: slopes continuously towards the rostral tip (0); with a strong inflection at the base of the ascending ramus, creating a rostral ramus with parallel dorsal and ventral margins (1). (Sereno *et al.* 1996)
25. Length of rostral ramus of the maxilla: less than its dorsoventral depth (0); greater than its dorsoventral depth (1). (Sereno *et al.* 1996)
26. Shape of the main body of the maxilla: tapering posteriorly (0); dorsal and ventral margins parallel for most of their length (1). (Yates 2007)
27. Shape of the ascending ramus of the maxilla in lateral view: tapering dorsally (0); with an anteroposterior expansion at the dorsal end (1). (Yates 2007)
28. Rostrocaudal length of the antorbital fossa: greater than that of the orbit (0); less than that of the orbit (1). (Yates 2003b)
29. Posteroventral extent of medial wall of antorbital fossa: reaching the anterior tip of the jugal (0); terminating anterior to the anterior tip of the jugal (1). (modified from Galton and Upchurch 2004).
30. Development of the antorbital fossa on the ascending ramus of the maxilla: deeply impressed and delimited by a sharp, scarp-like rim (0); weakly impressed and delimited by a rounded rim or a change in slope (1). (Yates 2007)
31. Shape of the antorbital fossa: crescentic with a strongly concave posterior margin that is roughly parallel to the anterior margin of the antorbital fossa (0); subtriangular with a straight to gently concave posterior margin (1); antorbital fossa absent (2). (modified from Galton 1985a)
32. Size of the neurovascular foramen at the posterior end of the lateral maxillary row: not larger than the others (0); distinctly larger than the others in the row (1). (Yates 2003b)
33. Direction that the neurovascular foramen at the posterior end of the lateral maxillary row opens: posteriorly (0); anteriorly, ventrally, or laterally (1). (modified from Sereno 1999)
34. Arrangement of lateral maxillary neurovascular foramina: linear (0); irregular (1). (modified from Sereno 1999)
35. Longitudinal ridge on the posterior lateral surface of the maxilla: absent (0); present (1). (Barrett et al. 2005a)
36. Dorsal exposure of the lachrymal: present (0); absent (1). (Gauthier 1986)
37. Shape of the lachrymal: dorsoventrally short and blockshaped (0); dorsoventrally elongate and shaped like an inverted L (1). (Rauhut 2003)
38. Orientation of the lachrymal orbital margin: strongly sloping anterodorsally (0); erect and close to vertical (1). (Yates 2007)
39. Length of the anterior ramus of the lachrymal: greater than half the length of the ventral ramus (0); less than half the length of the ventral ramus (1); absent altogether (2). (modified from Galton 1990)
40. Web of bone spanning junction between anterior and ventral rami of lachrymal: absent and antorbital fossa laterally exposed (0); present, obscuring posterodorsal corner of antorbital fossa (1). (Yates 2007)
41. Extension of the antorbital fossa onto the ventral end of the lachrymal: present (0); absent (1). (modified from Wilson and Sereno 1998)
42. Length of the posterior process of the prefrontal: short (0); elongated, so that total prefrontal length is equal to the anteroposterior diameter of the orbit (1). (Galton 1985a)
43. Ventral process of prefrontal extending down the posteromedial side of the lachrymal: present (0); absent (1). (Wilson and Sereno 1998)
44. Maximum transverse width of the prefrontal: less than 0.25 of the skull width at that level (0); more than 0.25 of the skull width at that level (1). (modified from Galton 1990)
45. Shape of the orbit: subcircular (0); ventrally constricted making the orbit subtriangular (1). (Wilson and Sereno 1998)
46. Slender anterior process of the frontal intruding between the prefrontal and the nasal: absent (0); present (1). (modified from Sereno 1999)
47. Jugal-lachrymal relationship: lachrymal overlapping lateral surface of jugal or abutting it dorsally (0); jugal overlapping lachrymal laterally (1). (Sereno et al. 1993)
48. Shape of the suborbital region of the jugal: an anteroposteriorly elongate bar (0); an anteroposteriorly shortened plate (1). (Yates 2007)
49. Jugal contribution to the antorbital fenestra: present (0); absent (1). (Holtz 1994)
50. Dorsal process of the anterior jugal: present (0); absent (1). (modified from Rauhut 2003)
51. Ratio of the minimum depth of the jugal below the orbit to the distance between the anterior end of the jugal and the anteroventral corner of the infratemporal fenestra: less than 0.2 (0); greater than 0.2 (1). (modified from Galton 1985a)
52. Transverse width of the ventral ramus of the postorbital: less than its anteroposterior width at midshaft (0); greater than its anteroposterior width at midshaft (1). (Wilson and Sereno 1998)
53. Shape of the dorsal margin of postorbital in lateral view: straight to gently curved (0); with a distinct embayment between the anterior and posterior dorsal processes (1). (Yates 2007)
54. Height of the postorbital rim of the orbit: flush with the posterior lateral process of the postorbital (0); raised so that it projects laterally to the posterior dorsal process (1). (Yates 2007)
55. Postfrontal bone: present (0); absent (1). (Sereno *et al.* 1993)
56. Position of the anterior margin of the infratemporal fenestra: behind the orbit (0); extends under the rear half of the orbit (1); extends as far forward as the midlength of the orbit (2). (modified from Upchurch 1995)
57. Frontal contribution to the supratemporal fenestra: present (0); absent (1). (modified from Gauthier 1986)
58. Orientation of the long axis of the supratemporal fenestra: longitudinal (0); transverse (1). (Wilson and Sereno 1998)
59. Medial margin of supratemporal fossa: simple smooth curve (0); with a projection at the frontal/postorbital-parietal suture producing a scalloped margin (1). (Leal et al. 2004)
60. Length of the quadratojugal ramus of the squamosal relative to the width at its base: less than four times its width (0); greater than four times its width (1). (Sereno 1999)
61. Proportion of infratemporal fenestra bordered by squamosal: more than 0.5 of the depth of the infratemporal fenestra (0); less than 0.5 of the depth of the infratemporal fenestra (1). (Yates 2007)
62. Squamosal-quadratojugal contact: present (0); absent (1). (Gauthier 1986)
63. Angle of divergence between jugal and squamosal rami of quadratojugal: close to 90 degrees (0); close to parallel (1). (Yates 2007)
64. Length of jugal ramus of quadratojugal: no longer than the squamosal ramus (0); longer than the squamosal ramus (1). (Wilson and Sereno 1998)
65. Shape of the rostral end of the jugal ramus of the quadratojugal: tapered (0); dorsoventrally expanded (1).
66. Relationship of quadratojugal to jugal: jugal overlaps the lateral surface of the quadratojugal (0); quadratojugal overlaps the lateral surface of the jugal (1); quadratojugal sutures along the ventrolateral margin of the jugal (2). (Yates 2007)
67. Position of the quadrate foramen: on the quadrate-quadratojugal suture (0); deeply incised into, and partly encircled by, the quadrate (1); on the quadrate-squamosal suture, just below the quadrate head (2). (modified from Rauhut 2003)
68. Shape of posterolateral margin of quadrate: sloping anterolaterally from posteromedial ridge (0); everted posteriorly creating a posteriorly facing fossa (1); posterior fossa deeply excavated, invading quadrate body (2). (Wilson and Sereno 1998)
69. Exposure of the lateral surface of the quadrate head: absent, covered by lateral sheet of the squamosal (0); present (1). (Sereno et al. 1993)
70. Proportion of the length of the quadrate that is occupied by the pterygoid wing: at least 70 per cent (0); greater than 70 per cent (1). (Yates 2003b)
71. Depth of the occipital wing of the parietal: less than 1.5 times the depth of the foramen magnum (0); more than 1.5 times the depth of the foramen magnum (1). (Wilson and Sereno 1998)
72. Position of foramina for mid-cerebral vein on occiput: between supraoccipital and parietal (0); on the supraoccipital (1). (modified from Yates 2003b)
73. Postparietal fenestra between supraoccipital and parietals: absent (0); present (1). (Yates 2007)
74. Shape of the supraoccipital: diamond-shaped, at least as high as wide (0); semilunate and wider than high (1). (Yates 2003c)
75. Orientation of the supraoccipital plate: erect to gently sloping (0); strongly sloping forward so that the dorsal tip lies level with the basipterygoid processes (1). (Galton and Upchurch 2004)
76. Orientation of the paroccipital processes in occipital view: slightly dorsolaterally directed to horizontal (0); ventrolaterally directed (1). (Rauhut 2003a)
77. Orientation of the paroccipital processes in dorsal view: posterolateral forming a Vshaped occiput (0); lateral forming a flat occiput (1) (Wilson 2002)
78. Size of the post-temporal fenestra: large fenestra (0); a small hole that is much less than half the depth of the paroccipital process (1). (Yates 2007)
79. Exit of the mid-cerebral vein: through trigeminal foramen (0) or through a separate foramen (1) (modified from Rauhut, 2003).
80. Shape of the floor of the braincase in lateral view: relatively straight with the basal tuberae, basipterygoid processes and parasphenoid rostrum roughly aligned (0); bent with the basipterygoid processes and the parasphenoid rostrum below the level of the basioccipital condyle and the basal tuberae (1); bent with the basal tuberae lowered below the level of the basioccipital and the parasphenoid rostrum raised above it (2). (modified from Galton 1990)
81. Basioccipital component of the basal tubera, medial component in relation to the parabasisphenoidal component: present (0); absent (1). (Modified from Yates, 2007)
82. Length of the basipterygoid processes (from the top of the parasphenoid to the tip of the process): less than the height of the braincase (from the top of the parasphenoid to the top of the supraoccipital) (0); greater than the height of the braincase (from the top of the parasphenoid to the top of the supraoccipital) (1). (Benton et al. 2000)
83. Basioccipital - basisphenoid junction on the ventral surface of the bones: straight line (0); U/V shaped (1) (Bronzati & Rauhut, 2017)
84. subsellar recess: maximum width equal or greater than the dorsoventral height (0); maximum width smaller than the dorsoventral height (1) (Bronzati & Rauhut, 2017)
85. Shape of jugal process of ectopterygoid: gently curved (0); strongly recurved and hooklike (1). (Yates 2003b)
86. Pneumatic fossa on the ventral surface of the ectopterygoid: present (0); absent (1). (Sereno *et al*. 1996)
87. Relationship of the ectopterygoid to the pterygoid: ectopterygoid overlapping the ventral surface of the pterygoid (0); ectopterygoid overlapping the dorsal surface of the pterygoid (1). (Sereno *et al.* 1993)
88. Position of the maxillary articular surface of the palatine: along the lateral margin of the bone (0); at the end of a narrow anterolateral process due to the absence of the posterolateral process (1). (Wilson and Sereno 1998)
89. Centrally located tubercle on the ventral surface of palatine: absent (0); present (1). (Yates 2007)
90. Medial process of the pterygoid forming a hook around the basipterygoid process: absent (0); flat and blunt-ended (1); bent upward and pointed (2). (modified from Wilson and Sereno 1998)
91. Length of the vomers: less than 0.25 of the total skull length (0); more than 0.25 of the total skull length (1). (Yates 2007)
92. Position of jaw joint: no lower than the level of the dorsal margin of the dentary (0); depressed, well below this level (1). (Sereno 1999)
93. Shape of upper jaws in ventral view: narrow with an acute rostral apex (0); broad and Ushaped (1). (Wilson and Sereno 1998)
94. Length of the external mandibular fenestra: more than 0.1 of the length of the mandible (0); less than 0.1 of the length of the mandible (1). (modified from Upchurch 1995)
95. Caudal end of dentary tooth row medially inset with a thick lateral ridge on the dentary forming a buccal emargination : absent (0); present (1). (Gauthier 1986)
96. Height : length ratio of the dentary: less than 0.2; greater than 0.2 (1). (modified from Benton *et al.* 2000)
97. Orientation of the symphyseal end of the dentary: in line with the long axis of the dentary (0); strongly curved ventrally (1). (Sereno 1999)
98. Position of first dentary tooth: adjacent to symphysis (0); inset one tooth's width from the symphysis (1). (Sereno 1999)
99. Dorsoventral expansion at the symphyseal end of the dentary: absent (0); present (1). (Wilson and Sereno 1998)
100. Splenial foramen: absent (0); present and enclosed (1); present and open anteriorly (2). (Rauhut 2003a)
101. Splenial-angular joint: flattened sutured contact (0); synovial joint surface between tongue-like process of angular fitting in groove of the splenial (1). (Sereno *et al.* 1993)
102. A stout, triangular, medial process of the articular, behind the glenoid : present (0); absent (1). (Yates 2003b)
103. Length of the retroarticular process: less than the depth of the mandible below the glenoid (0); greater than the depth of the mandible below the glenoid (1). (Yates 2003b)
104. Strong medial embayment behind glenoid of the articular in dorsal view: absent (0); present (1). (Yates & Kitching 2003)
105. Number of premaxillary teeth: four (0); more than four (1). (Galton 1990)
106. Number of dentary teeth (in adults): less than 18 (0); 18 or more (1). (modified from Wilson and Sereno 1998)
107. Arrangement of teeth within the jaws: linearly placed, crowns not overlapping (0); imbricated with distal side of tooth overlapping mesial side of the succeeding tooth (1). (Yates 2007)
108. Orientation of the maxillary tooth crowns: erect (0); procumbent (1). (modified from Gauthier 1986)
109. Orientation of the dentary tooth crowns: erect (0); procumbent (1). (modified from Gauthier 1986)
110. Teeth with basally constricted crowns: absent (0); present (1). (Gauthier 1986)
111. Tooth-tooth occlusal wear facets : absent (0); present (1). (Wilson and Sereno 1998)
112. Distribution of serrations on the maxillary and dentary teeth: present on both the mesial and distal carinae (0); absent on the posterior carinae (1); absent on both carinae (2). (Wilson 2002)
113. Long axis of the tooth crowns distally recurved: present (0); absent (1). (Gauthier 1986)
114. Texture of the enamel surface: entirely smooth (0); finely wrinkled in some patches (1); extensively and coarsely wrinkled (2). (modified from Wilson and Sereno 1998)
115. Lingual concavities of the teeth: absent (0); present (1). (Upchurch 1995)
116. Longitudinal labial grooves on the teeth: absent (0); present (1). (Upchurch 1998)
117. Distribution of the serrations along the mesial and distal carinae of the tooth: extend along most of the length of the crown (0); restricted to the upper half of the crown (1). (Yates 2003b)
118. Number of cervical vertebrae: eight or fewer (0); 9-10 (1); 12-13 (2); more than 13 (3). (modified from Wilson and Sereno 1998)
119. Shallow, dorsally facing fossa on the atlantal neurapophysis bordered by a dorsally everted lateral margin: absent (0); present (1). (Yates and Kitching 2003)
120. Width of axial intercentrum: less than width of axial centrum (0); greater than width of axial centrum (1). (Sereno 1999)
121. Position of axial prezygapophyses: on the anterolateral surface of the neural arch (0); mounted on anteriorly projecting pedicels (1). (Yates 2007)
122. Posterior margin of the axial postzygapophyses: overhang the axial centrum (0); flush with the caudal face of the axial centrum (1). (Sereno 1999)
123. Length of the axial centrum: less than three times the height of the centrum (0); at least three times the height of the centrum (1). (Yates 2007)
124. Length of the anterior cervical centra (cervicals 3-5): no more than the length of the axial centrum (0); greater than the length of the axial centrum (1). (Yates 2007)
125. Length of middle to posterior cervical centra (cervical 6-8): no more than the length of the axial centrum (0); greater than the length of the axial centrum (1). (Yates 2007)
126. Dorsal excavation of the cervical parapophyses: absent (0); present (1). (Upchurch 1998)
127. Lateral compression of the anterior cervical vertebrae: centra are no higher than they are wide (0); are approximately 1.25 times higher than wide (1). (Upchurch 1998)
128. Relative elongation of the anterior cervical centra (cervical 3-5): lengths of the centra are less than 2.5 times the height of their anterior faces (0); lengths are 2.5-4 times the height of their anterior faces (1); the length of at least cervical 4 or 5 exceeds 4 times the anterior centrum height (2). (modified from Sereno 1999)
129. Ventral keels on cranial cervical centra: present (0); absent (1). (modified from Upchurch 1998)
130. Height of the mid cervical neural arches: no more than the height of the posterior centrum face (0); greater than the height of the posterior centrum face (1). (Yates 2007)
131. Cervical epipophyses on the dorsal surface of the postzygapophyses: absent (0); present on at least some cervical vertebrae (1). (Yates 2007)
132. Posterior ends of the anterior, postaxial epipophyses: with a free pointed tip (0); joined to the postzygapophysis along their entire length (1). (Yates 2007)
133. Shape of the epipophyses: tall ridges (0); flattened, horizontal plates (1). (Yates 2003b)
134. Epipophyses overhanging the rear margin of the postzygapophyses: absent (0); present in at least some postaxial cervical vertebrae (1). (Sereno *et al.* 1993)
135. Anterior spur-like projections on mid-cervical neural spines: absent (0); present (1). (Yates 207)
136. Shape of mid-cervical neural spines: less than twice as long as high (0); at least twice as long as high (1). (Yates 2007)
137. Shape of cervical rib shafts: short and posteroventrally directed (0); longer than the length of their centra and extending parallel to cervical column (1). (Sereno 1999)
138. Position of the base of the cervical rib shaft: level with, or higher than the ventral margin of the cervical centrum (0); located below the ventral margin due to a ventrally extended parapophysis (1). (Wilson and Sereno 1998)
139. Postzygodiapophyseal lamina in cervical neural arches 4-8: present (0); absent (1). (Yates (2003b)
140. Laminae of the cervical neural arches 4-8: well-developed tall laminae (0); weakly developed low ridges (1). (Wilson and Sereno 1998)
141. Shape of anterior centrum face in cervical centra: concave (0); flat (1); convex (2). (modified from Gauthier 1986)
142. Ventral surface of the centra in the cervicodorsal transition: transversely rounded (0); with longitudinal keels (1). (Rauhut 2003a)
143. Number of vertebrae between cervicodorsal transition and primordial sacral vertebrae: 15-16 (0); no more than 14 (1). (modified from Wilson and Sereno 1998)
144. Lateral surfaces of the dorsal centra: with at most vague, shallow depressions (0); with deep fossae that approach the midline (1); with invasive, sharp-rimmed pleurocoels (2). (Gauthier 1986)
145. Oblique ridge dividing pleural fossa of cervical vertebrae: absent (0); present (1). (Wilson and Sereno 1998)
146. Laterally expanded tables at the midlength of the dorsal surface of the neural spines: absent in all vertebrae (0); present on the pectoral vertebrae (1); present on the pectoral and cervical vertebrae (2). (Yates and Kitching 2003)
147. Dorsal centra: entirely amphicoelous to amphiplatyan (0); first two dorsals are opisthocoelous (1); cranial half of dorsal column is opisthocoelous (2).(Wilson and Sereno 1998)
148. Shape of the posterior dorsal centra: relatively elongated for their size (0); strongly axially compressed for their size (1). (modified from Novas 1993)
149. Laminae bounding triangular infradiapophyseal fossae (chonae) on dorsal neural arches: absent (0); present (1). (Wilson 1999)
150. Location of parapophysis in first two dorsals: at the anterior end of the centrum (0); located at the mid-length of the centrum, within the middle chonos (1). (Yates 2007)
151. Parapophyses of the dorsal column completely shift from the centrum to the neural arch: anterior to the thirteenth presacral vertebra (0); posterior to the thirteenth presacral vertebra (1). (Langer 2004)
152. Orientation of the transverse processes of the dorsal vertebrae: most horizontally directed (0); all upwardly directed (1). (Upchurch 1998)
153. Contribution of the paradiapophyseal lamina to the margin of the anterior chonos in mid-dorsal vertebrae: present (0); prevented by high placement of parapophysis (1). (Yates 2007)
154. Hyposphenes in the dorsal vertebrae: absent (0); present but less than the height of the neural canal (1); present and equal to the height of the neural canal (2). (modified from Gauthier 1986)
155. Prezygodiapophyseal lamina and associated anterior triangular fossa (anterior infradiapophyseal fossa): present on all dorsals (0); absent in mid-dorsals (1). (Yates 2003b)
156. Anterior centroparapophyseal lamina in dorsal vertebrae: absent (0); present (1). (Wilson 2002)
157. Prezygoparapophyseal lamina in dorsal vertebrae: absent (0); present (1). (Yates 2007)
158. Accessory lamina dividing posterior chonos from postzygapophysis: absent (0); present (1). (Yates 2007)
159. Pneumatic excavation of the dorsal neural arches: absent (0); equivocal (e.g., no more than depressions within the infradiapophyseal chambers) (1); sharp-rimmed subfossae or foramina clearly invading bone surface (2). (modified from Sereno & Wilson 1998)
160. Separation of lateral surfaces of anterior dorsal neural arches under transverse processes: widely spaced (0); only separated by a thin midline septum (1). (Upchurch *et al.* 2004)
161. Height of dorsal neural arches, from neurocentral suture to level of zygapophyseal facets: much less than height of centrum (0); subequal to or greater than height of centrum (1). (Yates 2007)
162. Form of anterior surface of neural arch: simple centroprezygopophyseal ridge (0); broad anteriorly facing surface bounded laterally by centroprezygopophyseal lamina (1). (Bonaparte 1999)
163. Shape of posterior dorsal neural canal: subcircular (0); slit-shaped (1). (Wilson and Sereno 1998)
164. Height of middle dorsal neural spines: less than the length of the base (0); higher than the length of the base but less than 1.5 times the length of the base (1); greater than 1.5 times the length of the base (2). (modified from Bonaparte 1986b)
165. Shape of anterior dorsal neural spines: lateral margins parallel in anterior view (0); transversely expanding towards dorsal end (1). (Yates 2007)
166. Cross-sectional shape of dorsal neural spines: transversely compressed (0); broad and triangular (1); square-shaped in posterior vertebrae (2). (modified from Bonaparte 1986b)
167. Spinodiapophyseal lamina on dorsal vertebrae: absent (0); present and separated from spinopostzygapophyseal lamina (1); present and joining spinopostzygapophyseal lamina to create a composite posterolateral spinal lamina (2). (Wilson and Sereno 1998)
168. Well-developed, sheet-like suprapostzygapophyseal laminae: absent (0); present on at least the caudal dorsal vertebrae (1). (Bonaparte 1986b)
169. Shape of the spinopostzygapophyseal lamina in middle and posterior dorsal vertebrae: singular (0); bifurcated at its distal end (1). (Wilson 2002)
170. Shape of posterior margin of middle dorsal neural spines in lateral view: approximately straight (0); concave with a projecting posterodorsal corner (1). (Yates 2003c)
171. Transversely expanded plate-like summits of posterior dorsal neural spines: absent (0); present (1). (Novas 1993)
172. Last presacral rib: free (0); fused to vertebra (1). (Yates 2007)
173. Sacral rib much narrower than the transverse process of the first primordial sacral vertebra (and dorsosacral if present) in dorsal view: absent (0); present (1). (Yates and Kitching 2003)
174. Number of dorsosacral vertebrae: none (0); one (1); two (2). (modified from Gauthier 1986)
175. Caudosacral vertebra: absent (0); present (1). (Galton and Upchurch 2004)
176. Shape of the iliac articular facets of the first primordial sacral rib: singular (0); divided into dorsal and ventral facets separated by a non-articulating gap (1). (Yates 2007)
177. Deep, medially-directed pit excavating the surface of the non-articulating gap of the first primordial sacral rib: absent (0); present (1).
178. Depth of the iliac articular surface of the primordial sacrals: less than 0.75 of the depth of the ilium (0); greater than 0.75 of the depth of the ilium (1). (modified from Novas 1992)
179. Sacral ribs contributing to the rim of the acetabulum: absent (0); present (1). (Wilson 2002)
180. Posterior and anterior expansion of the transverse processes of the first and second primordial sacral vertebrae, respectively, partly roofing the intercostal space: absent (0); present (1). (Langer 2004)
181. Length of first caudal centrum: greater than its height (0); less than its height (1). (Yates 203b)
182. Position of postzygapophyses in proximal caudal vertebrae: protruding with an interpostzygapophyseal notch visible in dorsal view (0); placed on either side of the caudal end of the base of the neural spine without any interpostzygapophyseal notch (1). (Yates 2003b)
183. A hyposphenal ridge on caudal vertebrae: absent (0); present (1). (Upchurch 1995)
184. Prezygadiapophyseal laminae on anterior caudals: absent (0); present (1).
185. Depth of the bases of the proximal caudal transverse processes: shallow, restricted to the neural arches (0); deep, extending from the centrum to the neural arch (1). (Upchurch 1998)
186. Position of last caudal vertebra with a protruding transverse process: distal to caudal 16 (0); proximal to caudal 16 (1). (Wilson 2002)
187. Orientation of posterior margin of proximal caudal neural spines: sloping posterodorsally (0); vertical (1). (Novas 1992)
188. Longitudinal ventral sulcus on proximal and middle caudal vertebrae: present (0); absent (1). (modified from Upchurch 1995)
189. Length of midcaudal centra: greater than twice the height of their anterior faces (0); less than twice the height of their anterior faces (1). (Yates 2003a)
190. Cross-sectional shape of the distal caudal centra: oval with rounded lateral and ventral sides (0); square-shaped with flattened lateral and ventral sides (1). (Yates 2007a)
191. Length of distal caudal prezygapophyses: short, not overlapping the preceding centrum by more than a quarter (0); long and overlapping the preceding the centrum by more than a quarter (1). (Gauthier 1986)
192. Shape of the terminal caudal vertebrae: unfused, size decreasing toward tip (0); expanded and fused to form a club-shaped tail (1). (Upchurch 1995)
193. 'Weaponized' dermal spikes on tail: absent (0); present (1).
194. Length of the longest chevron: less than twice the length of the preceding centrum (0); greater than twice the length of the preceding centrum (1). (modified from Yates 2003a)
195. Anteroventral process on distal chevrons: absent (0); present (1). (Upchurch 1995)
196. Mid-caudal chevrons with a ventral slit: absent (0); present (1). (Upchurch 1995)
197. Longitudinal ridge on the dorsal surface of the sterna plate: absent (0); present (1). (Upchurch 1998)
198. Craniocaudal length of the acromion process of the scapula: less than 1.5 times the minimum width of the scapula blade (0); greater than 1.5 times the minimum width of the scapula blade (1). (Wilson and Sereno 1998)
199. Minimum width of the scapula: greater than 20 per cent of its length (0); less than 20 per cent of its length (1). (Gauthier 1986)
200. Caudal margin of the acromion process of the scapula: rises from the blade at angle that is less than 65 degrees from the long axis of the scapula, at its steepest point (0); rises from the blade at angle that is greater than 65 degrees from the long axis of the scapula, at its steepest point (1). (modified from Novas 1992)
201. Width of dorsal expansion of the scapula: less than the width of the ventral end of the scapula (0); equal to the width of the ventral end of the scapula (1). (Pol & Powell 2007)
202. Flat caudoventrally facing surface on the coracoids between glenoid and coracoid tubercle: absent (0); present (1). (Yates & Kitching 2003)
203. Coracoid tubercle: present (0); absent (1). (modified from Pérez-Moreno *et al.* 1994)
204. Length of the humerus: less than 55 per cent of the length of the femur (0); 55-65 per cent of the length of the femur (1); 65-70 per cent of the length of the femur (2); more than 70 per cent of the length of the femur (3). (modified from Gauthier 1986)
205. Shape of the humeral head: weakly developed, rounded in anterior-posterior view but minimally expanded perpendicular to the latter axis (0); flat in anterior-posterior view with only a slightly expanded lateral component (1); domed, being convex/hemispherical in anterior-posterior view with a strong lateral incursion onto the humeral shaft (2).
206. Humerus; deltopectoral crest shape: low rounded crest (0); subtriangular (1); subrectangular (2). (Yates, 2007).
207. Length of the deltopectoral crest of the humerus: less than 30 per cent of the length of the humerus (0); 30-50 per cent of the length of the humerus (1); greater than 50 per cent of the length of the humerus (2). (modified from Sereno *et al.* 1993)
208. Shape of the anterolateral margin of the deltopectoral crest of the humerus: straight (0); strongly sinuous (1). (Yates 2003)
209. Rugose pit centrally located on the lateral surface of the deltopectoral crest: absent (0); present (1). (Yates 2007)
210. Well-defined fossa on the distal flexor surface of the humerus: present (0); absent (1). (Yates & Kitching 2003)
211. Transverse width of the distal humerus: less than 33 per cent of the length of the humerus (0); greater than 33 per cent of the length of the humerus (1). (Langer 2004)
212. Shape of the entepicondyle of the distal humerus: rounded process (0): with a flat distomedially facing surface bounded by a sharp proximal margin (1). (Yates 2007)
213. Length of the radius: greater than 80 per cent of the humerus (0); less than 80 per cent of the humerus (1). (Langer 2004)
214. Deep radial fossa, bounded by an anterolateral process, on proximal ulna: absent (0); present (1). (Wilson and Sereno 1998)
215. Olecranon process on proximal ulna: present (0); absent (1); greatly enlarged olecranon (2). (Wilson and Sereno 1998)
216. Maximum linear dimensions of the ulnare and radiale: exceed that of at least one of the first three distal carpals (0); less than any of the distal carpals (1). (Yates 2003)
217. Transverse width of the first distal carpal: less than 120 per cent of the transverse width of the second distal carpal (0); greater than 120 per cent of the transverse width of the second distal carpal (1). (Sereno 1999)
218. Sulcus across the medial end of the first distal carpal:absent (0); present (1). (Yates 2007)
219. Lateral end of first distal carpal: abuts second distal carpal (0); overlaps second distal carpal (1). (Yates 2003)
220. Second distal carpal: completely covers the proximal end of the second metacarpal (0); does not completely cover the proximal end of the second metacarpal (1). (Yates & Kitching 2003)
221. Ossification of the fifth distal carpal: present (0); absent (1). (Yates 2007)
222. Length of the manus: less than 38 per cent of the humerus + radius (0); 38-45 per cent of the humerus + radius (1); greater than 45 per cent of the humerus + radius (2). (modified from Sereno *et al.* 1993)
223. Shape of metacarpus: flattened to gently curved and spreading (0); a colonnade of subparallel metacarpals tightly curved into a U-shape (1). (Wilson & Sereno 1998)
224. Proximal width of first metacarpal: less than the proximal width of the second metacarpal (0); greater than the proximal width of the second metacarpal (1). (modified from Gauthier 1986)
225. Minimum transverse shaft width of first metacarpal: less than twice the minimum transverse shaft width of second metacarpal (0); greater than twice the minimum transverse shaft width of second metacarpal (1). (Yates 2007)
226. Proximal end of first metacarpal: flush with other metacarpals (0); inset into the carpus (1). (Sereno 1999)
227. Shape of the first metacarpal: proximal width less than 65 per cent of its length (0); proximal width 65-80 per cent of its length (1); proximal width 80-100 per cent of its length (2); greater than 100 per cent of its length (3). (modified from Sereno 1999)
228. Strong asymmetry in the lateral and medial distal condyles of the first metacarpal: absent (0); present (1). (Gauthier 1986)
229. Deep distal extensor pits on the second and third metacarpals: absent (0); present (1). (Novas 1993)
230. Shape of the distal ends of second and third metacarpals: subrectangular in distal view (0); trapezoidal with flexor rims of distal collateral ligament pits flaring beyond extensor rims (1). (Yates 2007)
231. Shape of the fifth metacarpal: longer than wide at the proximal end with a flat proximal surface (0); almost as wide as it is long with a strongly convex proximal articulation surface (1). (Yates 2003)
232. Length of the fifth metacarpal: less than 75 per cent of the length of the third metacarpal (0); greater than 75 per cent of the length of the third metacarpal (1). (Upchurch 1998)
233. Length of manual digit one: less than the length of manual digit two (0); greater than the length of manual digit two (1). (Yates 2003a)
234. Ventrolateral twisting of the transverse axis of the distal end of the first phalanx of manual digit one relative to its proximal end: absent (0); present but much less than 60 degrees (1); 60 degrees (2). (Sereno 1999)
235. Length of the first phalanx of manual digit one: less than the length of the first metacarpal (0); greater than the length of the first metacarpal (1). (Gauthier 1986)
236. Shape of the proximal articular surface of the first phalanx of manual digit one: rounded (0); with an embayment on the medial side (1). (modified from Sereno 1999)
237. Shape of the first phalanx of manual digit one: elongate and subcylindrical (0); strongly proximodistally compressed and wedge-shaped (1). (Wilson 2002)
238. Length of the penultimate phalanx of manual digit two: less than the length of the second metacarpal (0); greater than the length of the second metacarpal (1). (Rauhut 2003)
239. Length of the penultimate phalanx of manual digit three: less than the length of the third metacarpal (0); greater than the length of the third metacarpal (1).
240. Shape of non-terminal phalanges of manual digits two and three: longer than wide (0); as long as wide (1). (Rauhut 2003)
241. Shape of the unguals of manual digits two and three: straight (0); strongly curved with tips projecting well below flexor margin of proximal articular surface (1). (Sereno *et al.* 1993)
242. Length of the ungual of manual digit two: greater than the length of the ungual of manual digit one (0); 75-100 per cent of the ungual of manual digit one (1); less than 75 per cent of the ungual of manual digit one (2); the ungual of manual digit two is absent (3). (modified from Gauthier 1986)
243. Phalangeal formula of manual digits two and three: three and four, respectively (0); with at least one phalanx missing from each digit (1). (modified from Sereno & Wilson 1998)
244. Phalangeal formula of manual digits four and five: greater than 2-0, respectively (0); less than 2-0, respectively (1). (Gauthier 1986)
245. Strongly convex dorsal margin of the ilium: absent (0); present (1). (Gauthier 1986)
246. Cranial extent of preacetabular process of ilium: does not project further anterior than the anterior margin of the pubic peduncle (0); projects anterior to the cranial margin of the pubic peduncle (1). (Yates 2003)
247. Shape of the preacetabular process: blunt and rectangular (0); with a pointed, projecting anteroventral corner and a rounded dorsum (1). (modified from Sereno 1999)
248. Depth of the preacetabular process of the ilium: much less than the depth of the ilium above the acetabulum (0); subequal to the depth of the ilium above the acetabulum (1). (modified from Gauthier 1986)
249. Length of preacetabular process of the ilium: less than twice its depth (0); greater than twice its depth (1).
250. Buttress between preacetabular process and the supraacetabular crest of the ilium: present (0); absent (1). (Gauthier 1986)
251. Medial wall of acetabulum: fully closing acetabulum with a triangular ventral process between the pubic and ischial peduncles (0); partially open acetabulum with a straight ventral margin between the peduncles (1); partially open acetabulum with a concave ventral margin between the peduncles (2); fully open acetabulum with medial ventral margin closely approximating lateral rim of acetabulum (3). (modified from Gauthier 1986)
252. Length of the pubic peduncle of the ilium: less than twice the anteroposterior width of its distal end (0); greater than twice the anteroposterior width of its distal end. (Sereno 1999)
253. Caudally projecting ‘heel’ at the distal end of the ischial peduncle: absent (0); present (1). (Yates 2003b)
254. Length of the ischial peduncle of the ilium: similar to pubic peduncle (0); much shorter than pubic peduncle (1); virtually absent so that the chord connecting the distal end of the pubic peduncle with the ischial articular surface contacts the postacetabular process (2). (Upchurch *et al.* 2004)
255. Well-developed brevis fossa with sharp margins on the ventral surface of the postacetabular process of the ilium: absent (0); present, ventrally facing (1); present, lateroventrally facing (2). (modified from Gauthier 1986)
256. Anterior end of ventrolateral ridge bounding brevis fossa: not connected to supracetabular crest (0); joining supracetabular crest (1). (Yates 2007)
257. Shape of the caudal margin of the postacetabular process of the ilium: rounded to bluntly pointed (0); square ended (1); with a pointed ventral corner and a rounded caudodorsal margin (2). (Yates 2003)
258. Width of the conjoined pubes: less than 75 per cent of their length (0); greater than 75 per cent of their length (1). (Cooper 1984)
259. Pubic tubercle on the lateral surface of the proximal pubis: present (0); absent (1). (Yates 2003a)
260. Proximal anterior profile of pubis: anterior margin of pubic apron smoothly confluent with anterior margin of iliac pedicel (0); iliac pedicel set anterior to the pubic apron creating a prominent inflection in the proximal anterior profile of the pubis (1). (Yates 2007)
261. Minimum transverse width of the pubic apron: much more than 40 per cent of the width across the iliac peduncles of the ilium (0); less than 40 per cent of the width across the iliac peduncles of the ilium (1).
262. Position of the obturator foramen of the pubis: at least partially occluded by the iliac pedicel in anterior view (0); completely visible in anterior view (1). (Galton & Upchurch 2004)
263. Lateral margins of the pubic apron in anterior view: straight (0); concave (1). (Yates & Kitching 2003)
264. Orientation of distal third of the blades of the pubic apron: confluent with the proximal part of the pubic apron (0); twisted posterolaterally relative to proximal section so that the anterior surface turns to face laterally (1). (Langer 2004)
265. Orientation of the entire blades of the pubic apron: transverse (0); twisted posteromedially (1). (modified from Langer 2004)
266. Craniocaudal expansion of the distal pubis: absent (0); less than 15 per cent of the length of the pubis (1); greater than 15 per cent of the length of the pubis (2). (modified from Gauthier 1986)
267. Notch separating posteroventral end of the ischial obturator plate from the ischial shaft: present (0); absent (1). (Rauhut 2003)
268. Elongate interischial fenestra: absent (0); present (1). (Yates 2003)
269. Longitudinal dorsolateral sulcus on proximal ischium: absent (0); present (1). Yates (2003)
270. Shape of distal ischium: broad and plate-like, not distinct from obturator region (0); with a discrete rod-like distal shaft (1). (Yates 2007)
271. Length of ischium: less than that of the pubis (0); greater than that of the pubis (1). (Salgado *et al.* 1997)
272. Ischial component of acetabular rim: larger than the pubic component (0); equal to the pubic component (1) (Galton & Upchurch 2004)
273. Shape of the transverse section of the ischial shaft: ovoid to subrectangular (0); triangular (1). (Sereno 1999)
274. Orientation of the long axes of the transverse section of the distal ischia: meet at an angle (0); are coplanar (1). (Wilson and Sereno 1998)
275. Depth of the transverse section of the ischial shaft: much less than the transverse width of the section (0); at least as great as the transverse width of the section (1). (Wilson and Sereno 1998)
276. Distal ischial expansion: absent (0); present (1). (Holtz 1994)
277. Transverse width of the conjoined distal ischial expansions: greater than their sagittal depth (0); less than their sagittal depth (1). (Yates 2003)
278. Length of the hindlimb: greater than the length of the trunk (0); less than the length of the trunk (1). (Gauthier 1986)
279. Longitudinal axis of the femur in lateral view: strongly bent with an offset between the proximal and distal axes greater than 15 degrees (0); weakly bent with an offset of less than 10 degrees (1); straight (2). (Cooper 1984)
280. Shape of the cross-section of the mid-shaft of the femur: subcircular (0); strongly elliptical with the long axis orientated mediolaterally (1). (Wilson and Sereno 1998)
281. Angle between the long axis of the femoral head and the transverse axis of the distal femur: about 30 degrees (0); close to 0 degrees (1). (Carrano 2000)
282. Shape of femoral head: roughly rectangular in profile with a sharp medial distal corner (0); roughly hemispherical with no sharp medial distal corner (1). (modified from Yates 2007) This character only applies to taxa with a medially, or anteromedially protruding femoral head. It does not apply to outgroup taxa (*Euparkeria* or Crurotarsi) with proximally directed femoral heads and is coded as non-applicable in these taxa.
283. Posterior proximal tubercle on femur: well-developed (0); indistinct to absent (1). (Novas 1996)
284. Shape of the lesser trochanter: small rounded tubercle (0); proximodistally orientated, elongate ridge (1); absent (2). (modified from Gauthier 1986)
285. Position of proximal tip of lesser trochanter: level with the femoral head (0); distal to the femoral head (1). (Galton & Upchurch 2004)
286. Projection of the lesser trochanter: just a scar upon the femoral surface (0); a raised process (1).
287. Transverse ridge extending laterally from the lesser trochanter: absent (0); present (1). (Rowe 1989)
288. Height of the lesser trochanter in cross section: less than its basal width (0); at least as high as its basal width (1). (modified from Galton 1990)
289. Position of the lesser trochanter in anterior view: near the centre of the anterior face of the femoral shaft (0); close to the lateral margin of the femoral shaft (1).
290. Visibility of the lesser trochanter in posterior view: not visible (0); visible (1). (Galton & Upchurch 2004)
291. Height of the fourth trochanter: a low rugose ridge (0); a tall crest (1). (Gauthier 1986)
292. Position of the fourth trochanter along the length of the femur: in the proximal half (0); straddling the midpoint (1). (Galton 1990)
293. Symmetry of the profile of the fourth trochanter of the femur: subsymmetrical without a sharp distal corner (0); asymmetrical with a steeper distal slope than the proximal slope and a distinct distal corner (1). (Langer 2004)
294. Shape of the profile of the fourth trochanter of the femur: rounded (0); subrectangular (1).
295. Position of fourth trochanter along the mediolateral axis of the femur: centrally located (0); on the medial margin (1). (Galton 1990)
296. Extensor depression on anterior surface of the distal end of the femur: absent (0); present (1). (Molnar *et al.* 1990)
297. Size of the medial condyle of the distal femur: subequal to the fibular + lateral condyles (0); larger than the fibular + lateral condyles (1). (modified from Wilson 2002)
298. Well-developed tibiofibular crest on distal femur: absent (0); present (1).
299. Distal surface of tibiofibular crest: as deep anteroposteriorly as wide mediolaterally or deeper (0); wider mediolaterally than deep anteroposteriorly (1).
300. Tibia:femur length ratio: greater than 1.0 (0); between 0.6 and 1.0 (1); less than 0.6 (2). (modified from Gauthier 1986)
301. Orientation of cnemial crest: projects anteriorly to anterolaterally (0); projecting laterally (1). (Wilson and Sereno 1998)
302. Paramarginal ridge on lateral surface of cnemial crest: absent (0); present (1). (Yates 2007)
303. Position of the tallest point of the cnemial crest: close to the proximal end of the crest (0); about half-way along the length of the crest, creating an anterodorsally sloping proximal margin of the crest (1). (Yates 2007)
304. Proximal end of tibia with a flange of bone that contacts the fibula: absent (0): present (1). (Gauthier 1986)
305. Position of the posterior end of the fibular condyle on the proximal articular surface tibia: anterior to the posterior margin of the proximal articular surface (0); level with the posterior margin of the proximal articularsurface (1). (Yates 2007)
306. Shape of the proximal articular surface of the tibia: transverse width subequal to anteroposterior length (0); transverse width between 0.6 and 0.9 times anteroposterior length (1); anteroposterior length twice the transverse width or higher (2). (Wilson & Sereno 1998)
307. Transverse width of the distal tibia: subequal to its craniocaudal length (0); greater than its craniocaudal length (1). (Gauthier 1986)
308. Anteroposterior width of the lateral side of the distal articular surface of the tibia: as wide as the anteroposterior width of the medial side (0); narrower than the anteroposterior width of the medial side (1). (Yates 2007)
309. Relationship of the posterolateral process of the distal end of the tibia with the fibula: not flaring laterally and not making significant contact with the fibula (0); flaring laterally and backing the fibula (1). (Yates 2007)
310. Shape of the distal articular end of the tibia in distal view: ovoid (0); subrectangular (1). (Yates 2007)
311. Shape of the anteromedial corner of the distal articular surface of the tibia: forming a right angle (0); forming an acute angle (1). (Langer 2004)
312. Position of the lateral margin of descending caudoventral process of the distal end of the tibia: protrudes laterally at least as far as the anterolateral corner of the distal tibia (0); set well back from the anterolateralcorner of the distal tibia (1). (Wilson and Sereno 1998)
313. A triangular rugose area on the medial side of the fibula: absent (0); present (1). (Wilson and Sereno 1998)
314. Transverse width of the midshaft of the fibula: greater than 0.75 of the transverse width of the midshaft of the tibia (0); between 0.5 and 0.75 of the transverse width of the midshaft of the tibia (1); less than 0.5 of thetransverse width of the midshaft of the tibia (2). (Langer 2004)
315. Position of fibula trochanter: on anterior surface of fibula (0); laterally facing (1); anteriorly facing but with strong lateral bulge (2). (modified from Wilson & Sereno 1998)
316. Depth of the medial end of the astragalar body in cranial view: roughly equal to the lateral end (0); much shallower creating a wedge-shaped astragalar body (1). (Wilson & Sereno 1998)
317. Shape of the posteromedial margin of the astragalus in dorsal view: forming a moderately sharp corner of a subrectangular astragalus (0); evenly rounded without formation of a caudomedial corner (1). (Wilson & Sereno 1998)
318. Dorsally facing horizontal shelf forming part of the fibular facet of the astragalus: present (0); absent with a largely vertical fibular facet (1). (Sereno 1999)
319. Pyramidal dorsal process on the posteromedial corner of the astragalus: absent (0); present (1).
320. Shape of the ascending process of the astragalus: anteroposteriorly deeper than transversely wide (0); transversely wider than anteroposteriorly deep (1). (Yates 2007)
321. Posterior extent of ascending process of the astragalus: positioned anteriorly upon the astragalus (0); close to the posterior margin of the astragalus (1). (Wilson and Sereno 1998)
322. Sharp medial margin around the depression posterior to the ascending process of the astragalus: absent (0); present (1). (Novas 1996)
323. Buttress dividing posterior fossa of astragalus and supporting ascending process: absent (0); present (1). (Wilson and Sereno 1998)
324. Vascular foramina set in a fossa at the base of the ascending process of the astragalus: present (0); absent (1). (Wilson and Sereno 1998)
325. Distal articular surface of astragalus: relatively flat or weakly convex (0); extremely convex and roller-shaped (1).
326. Transverse width of the calcaneum: greater than 30 per cent of the transverse width of the astragalus (0); less than 30 per cent of the transverse width of the astragalus (1). (Yates & Kitching 2003)
327. Lateral surface of calcaneum: simple (0); with a fossa (1). (Yates 2007a)
328. Medial peg of calcaneum fitting into astragalus: present, even if rudimentary (0); absent (1). (Sereno *et al.* 1993)
329. Calcaneal tuber: large and well developed (0); highly reduced to absent (1). (Yates 2007)
330. Shape of posteromedial heel of distal tarsal four (lateral distal tarsal): proximodistally deepest part of the bone (0); no deeper than the rest of the bone (1). (Langer 2004)
331. Shape of posteromedial process of distal tarsal four in proximal view: rounded (0); pointed (1). (Langer 2004)
332. Ossified distal tarsals: present (0); absent (1).
333. Proximal width of the first metatarsal: less than the proximal width of the second metatarsal (0); at least as great as the proximal width of the second metatarsal (1). (modified from Wilson and Sereno 1998)
334. Size of first metatarsal: maximum proximal breadth less than 0.4 times its proximodistal length (0); maximum proximal breadth between 0.4 and 0.7 times its proximodistal length (1); maximum proximal breadth greater than 0.7times its proximodistal length (2).
335. Orientation of proximal articular surface of metatarsal one: horizontal (0); sloping proximolaterally relative to the long axis of the bone (1). (Wilson 2002)
336. Shaft of metatarsal I: closely appressed to metatarsal II throughout its length (0); only closely appressed proximally, with a space between metatarsals I and II distally (1).
337. Orientation of the transverse axis of the distal end of metatarsal one: horizontal (0); angled proximomedially (1). (Wilson 2002)
338. Shape of the medial margin of the proximal surface of the second metatarsal: straight (0); concave (1). (modified from Sereno 1999)
339. Shape of the lateral margin of the proximal surface of the second metatarsal: straight (0); concave (1). (modified from Sereno 1999)
340. Projection of ventral flange on proximal surface of second metatarsal: neither corner appreciably more developed than the other (0); laterally flaring (1); medially flaring (2).
341. Well-developed facet on proximolateral corner of plantar ventrolateral flange of mt II for articulation with medial distal tarsal: absent (0); present (1).
342. Length of the third metatarsal: greater than 40 per cent of the length of the tibia (0); less than 40 per cent of the length of the tibia (1). (Gauthier 1986)
343. Proximal outline of metatarsal III: subtriangular with acute or rounded posterior border (0); subtrapezoidal, with posterior border broadly exposed in plantar view (1).
344. Minimum transverse shaft diameters of third and fourth metatarsals: greater than 60 per cent of the minimum transverse shaft diameter of the second metatarsal (0); less than 60 per cent of the minimum transverse shaft diameter ofthe second metatarsal (1). (Wilson and Sereno 1998)
345. Transverse width of the proximal end of the fourth metatarsal: less than twice the anteroposterior depth of the proximal end (0); at least twice the anteroposterior depth of the proximal end (1). (modified from Sereno 1999)
346. Angle formed by the anterior and anteromedial borders of metatarsal IV: obtuse (0); right angle, or acute (1).
347. Transverse width of the proximal end of the fifth metatarsal: less than 25 percent of the length of the fifth metatarsal (0); between 30 and 49 percent of the length of the fifth metatarsa (1); greater than 50 percent of the length of the fifth metatarsal (2). (modified from Sereno 1999)
348. Transverse width of distal articular surface of metatarsal four in distal view: greater than the anteroposterior depth (0); less than the anteroposterior depth (1). (Sereno 1999)
349. Pedal digit five: reduced, non-weight bearing (0); large (fifth metatarsal at least 70 per cent of fourth metatarsal), robust and weight bearing (1). (Wilson and Sereno 1998)
350. Length of non-terminal pedal phalanges: all longer than wide (0); proximalmost phalanges longer than wide while more distal phalanges are as wide as long (1); all nonterminal phalanges are as wide, if not wider, than long (2). (modified from Wilson and Sereno 1998)
351. Length of the first phalanx of pedal digit one: greater than the length of the ungual of pedal digit one (0); less than the length of the ungual of pedal digit one (1). (Yates & Kitching 2003)
352. Length of the ungual of pedal digit one: less than at least some non-terminal phalanges (0); longer than all non-terminal phalanges but shorter than first metatarsal (1); longer than the first metatarsal (2). (Yates 2007)
353. Shape of the ungual of pedal digit one: shallow, pointed, with convex sides and a broad ventral surface (0); deep, abruptly tapering, with flattened sides and a narrow ventral surface (1). (Wilson and Sereno 1998)
354. Shape of proximal articular surface of pedal unguals: proximally facing, visible on medial and lateral sides (0); proximomedially facing and visible only in medial view, causing medial deflection of pedal unguals in articulation (1). (Wilson and Sereno 1998)
355. Penultimate phalanges of pedal digits two and three: well-developed (0); reduced discshaped elements if they are ossified at all (1). (Wilson and Sereno 1998)
356. Shape of the unguals of pedal digits two and three: dorsoventrally deep with a proximal articulating surface that is at least as deep as it is wide (0); dorsoventrally flattened with a proximal articulating surface that is wider than deep (1). (Wilson and Sereno 1998)
357. Length of the ungual of pedal digit two: greater than the length of the ungual of pedal digit one (0); between 90 and 100 per cent of the length of the ungual of pedal digit one (1); less than 90 per cent of the length of the ungual of pedal digit one (2). (modified from Gauthier 1986)
358. Size of the ungual of pedal digit three: greater than 85 per cent of the ungual of pedal digit two in all linear dimensions (0); less than 85 per cent of the ungual of pedal digit two in all linear dimensions (1). (Yates 2003)
359. Number of phalanges in pedal digit four: four (0); fewer than four (1). (Gauthier 1986)
360. Phalanges of pedal digit five: present (0); absent (1). (Gauthier 1986)
361. Femoral length: less than 200 mm (0); between 200 and 399 mm (1); between 400 and 599 mm (2); between 600 and 799 mm (3); between 800 and 1000 mm (4); greater than 1000 mm (5). (modified from Yates 2004)
362. Laminae/ridges extending from the basipterygoid process onto the parasphenoid rostrum: converge anteromedially on the ventral surface of the cultriform process (0); extend parallel untill they fade into the ventral margin of the cultriform process (1). (Bronzati & Rauhut 2017)
363. Angle between basipterygoid process and cultriform process of the parabasisphenoid: < 90 degress (0); 90 degress (1); > 90 degrees (2). (modified from Butler *et al.,* 2008)
364. Length of the basisphenoid (from the basipterygoid process to the basisphenoidal component of the basal tubera) in relation to the length of the basioccipital (from the basioccipital component of the basal tubera to posterior limit of the condyle): longer or equal (0); shorter (1). (Bronzati & Rauhut 2017)
365. Notch in the posterodorsal margin of the lateral portion of the parabasisphenoid: absent (0); present (1). (Bronzati & Rauhut 2017)
366. Number of foramina in the otoccipital between the exoccipital pillar (excluding the foramina for the hypoglossal nerve) posteriorly and fenestra ovalis anteriorly: one (0), two (1). (Bronzati & Rauhut 2017)
367. Unossified gap between the basioccipital and basisphenoidal component of the basal tubera and ventral ramus of the opistothic: absent (0); present (1). (Bronzati & Rauhut 2017)
368. Otosphenoidal crest: low and not projecting posterolaterally (i.e. does not cover the fenestra ovalis with the braincase in lateral view) (0); developed as a lamina projecting posterolaterally (i.e. cover the fenestra ovalis with the braincase in lateral view) (1). (new character)
369. Parietal, distance separating supratemporal fenestrae: less than (0); or twice the long axis of supratemporal fenestra (1). (Wilsson, 2002: character 24).
370. Supratemporal region, anteroposterior length: temporal bar longer (0); or shorter anteroposteriorly than transversely (1). (Wilson, 2002: character 28).
371. Premaxilla, first tooth, position: adjacent to rostral tip (0); retreated (1). Pretto et al. (2018)
372. Premaxilla-maxilla articulation, alveolar margin. straight (0), excavated/concave, forming a subnarial gap (1). Gauthier (1986)
373. Subnarial foramen, position: at the ventral margin (0); displaced dorsally (1); displaced dorsally and positioned on the rim of, or inside, the narial fossa (2). Pretto et al. (2018)
374. Subnarial foramen, size: absent (0); small (no larger than adjacent maxillary neurovascular foramina) (1); large (2). Sereno *et al.* 1993.
375. Maxilla, antorbital fossa, ventral margin: sharp/smooth (0), ventrally bounded by a rounded ridge (1). Rowe & Gauthier (1990)
376. Maxilla, antorbital fossa, rostral portion: smooth (0), marked by a promaxillary fossa/fenestra (1). Cabreira et al. (2016)
377. Maxilla, external antorbital fenestra (i.e., fossa), rostral margin: rounded or pointed - frequently part of a triangular fenestra with central dorsal apex (0); squared - frequently part of a D-shaped fenestra (1). Rauhut (2003)
378. Maxilla, antorbital fossa, extension: extends the whole ventral border of the external antorbital fenestra (0); does not reach the caudal edge of the external antorbital fenestra (1). Langer (2004)
379. Maxilla, base of the dorsal ramus, rostral portion: smooth (0), perforated by a large foramen (1). New
380. Maxilla, the antorbital fossa, floor: smooth (0); perforated by a dorsally open neurovascular canal (1). Witmer (1997)
381. Prefrontal, rostroventral margin between rostral and ventral processes: rostroventrally expanded by a bone sheet (0); smooth/concave (1). Cabreira et al. (2011)
382. Lacrimal, rostral ramus, laterodorsal corner: smooth/featureless (0), formed by a rugose ridge (1). New
383. Lacrimal, antorbital fossa, dorsocaudal corner, exposure: exposed in lateral view (0); covered by a lateral bone lamina (1). Ezcurra & Novas (2007)
384. Jugal, caudal ramus, forked portion: does not reach base of the dorsal ramus (0), extends to the base of dorsal ramus (1). Nesbitt (2011)
385. Jugal, rostral and caudal rami, ventral margins: straight or forming an angle of more than 180° (0); forming angle of less than 180° (1). Cabreira et al. (2011)
386. Jugal, caudal process, extension: reaches the caudal margin of the lower temporal fenestra (0); terminates rostral to the caudal margin of the lower temporal fenestra (1). Cabreira et al. (2016)
387. Frontal, length relative to its minimal breadth on the orbital margin: more than four times (0); less than four times (1). New
388. Parietal, rostrolateral process, length relative to the lateromedial breadth of the rostral margin of the bone: less than 1/4 (0); more than 1/4 (1). New
389. Dentary, rostral tip, dorsal surface: at nearly the same plane as the rest of the alveolar margin of the bone (0); ventrally inclined (1). Sereno (1999)
390. Dentary, lateral surface directly below the first three teeth, within the central area of the lateral surface of the dentary: smooth (0); perforated by anterior dentary foramina (1). New
391. Surangular, lateral surface: evenly convex (0); bears a prominent horizontal shelf (1). Tykoski & Rowe (2004)
392. Maxillary teeth, number: 20 or less (0); more than 20 (1). New
393. Central maxillary and dentary tooth crowns, distal margin, shape: concave (0); sigmoid or convex (1). New
394. Central maxillary and dentary tooth crowns, denticles, size: small, more than six per mm (0); large, six or less per mm (1). New
395. Maxillary and dentary tooth crowns, denticles, angles formed to the margin of the crown: right = c. 90 defrees (0); upwards at an angle of 45 degrees (1). Benton *et al.* (2000)
396. Central maxillary and dentary tooth crowns, distal margin, denticles: reach the base of the crown (0); absent from the base of the crown (1). New
397. Premaxilla, first tooth, carina: with denticles/serrations (0); smooth (1). New
398. Premaxilla, teeth 1-3, mesial carina: with denticles/serrations (0); smooth (1). Rowe (1989).
399. Dentary, first tooth, mesiodistal breadth relative to those of teeth 4-10: subequal (0); narrower, about half the breadth(1). New
400. Premaxilla, first tooth crown, height: significantly shorter than most upper jaw crowns (0); at least as high as the tallest maxillary tooth crown (1). Pretto et al. (2018)
401. Second primordial sacral vertebra, neural spine, direction: vertical (0), dorsocaudally oriented (1). New
402. Scapula, blade, length relative to its minimal craniocaudal breadth: longer than six times (0), equal or shorter than six times (1). New
403. Metacarpal I, width (at the middle of the shaft)/length ratio: less than 0.35 (0); equal or more than 0.35 (1). Bakker & Galton (1974)
404. Ilium, postacetalular ala, caudal part, relative ventral projection of the shelves: brevis shelf projects more ventrally (0); “posteromedial” shelf projects more ventrally (1). New
405. Ilium, supraacetabular crest, extension on pubic peduncle: ends before the distal margin of the peduncle (0); extends along the peduncle length (1). Nesbitt et al. (2009)
406. Ilium, pubic peduncle, dorsal margin transverse shape: rounded (0); sharp (1). New
407. Ilium, ischiadic peduncle, orientation in lateral view: mainly vertical (0); oblique (ventrocaudal) as to expand well caudal to the cranial margin of the postacetabular embayment (1). Langer & Benton (2006)
408. Ilium, postacetabular ala, length relative to the space between the pre and post- acetabular embayments: shorter than or subequal (0); longer (1). Yates (2007)
409. Ilium, pubic peduncle, long axis, angle relative to the long axis of the iliac lamina: more than 45 degrees (0); about 45 degrees (1); less 45 degrees (2). Cabreira et al. (2016)
410. Pubis, medial articulation of the pair: complete, reaches the distal edge of the pubis (0); forms a medial hiatus (bevel) on the distal portion (1). Tykoski (2005)
411. Femur, fibular condyle, size relative to the medial condyle: subequal or smaller (0); larger (1). New
412. Tibia, distal end, caudal margin, outline in distal view: straight or convex (0); concave (1). Irmis et al. (2007)
413. Fibula, distal margin, angle relative to long axis of the bone: perpendicular (0); oblique (1). New

**Reference cited**

Bronzati M, Benson RBJ, Rauhut OWM. Rapid transformation in the braincase of sauropod dinosaurs: integrated evolution of the braincase and neck in early sauropods? Palaeontol. 61: 289–302.
