# Supplementary material for: Anatomy of the dinosaur Pampadromaeus barberenai (Saurischia—Sauropodomorpha) from the Late Triassic Santa Maria Formation of southern Brazil
Source: PLoS One. 2019 Feb 20;14(2):e0212543. doi: 10.1371/journal.pone.0212543 (PMC6382151; doi:10.1371/journal.pone.0212543)
Supplement: S2 File — (DOCX) [file pone.0212543.s003.DOCX]

**S2 File: Character-taxon matrix**

Added or independently scored taxa in blue.

“N” = not-applicable

***Aardonyx***

1101?0021000?11?11?????1110?1011000??????1010??0?11010???0?1????????1?0?????????0????????1??0?0?1?0?????11?0010011001?1??????0010?110100101101?0000011?00110001000020000010?1100??0110000?01110?0?00?????11??????????00??00??????13101???1?10??10????????????????1110100010011??10110?101?1111000011110001????????1101110?0???01???????????01201111?0?0010?0?2??00?0???03?????????102200000???????1?0000?11???????1??????????

***Adeopapposaurus***

100110021000111011111011011111010000101101000110111111110001000102001(0/1)0011010110001101101000001011(0/1)1010101111110(0/1)100111001111002001000111011011002001110011000000000000001001101?001000000000100000000100101211000111001101111011121011001110000020000100031101000110110010?1100101100000011010000102101010100000111011001001111010000111010000111100000102000110000110011001?1000102200001?0?001110110011111001?01000000?00?

***Anchisaurus***

10???00??010?1??11?????111011?010?001010?10001100111101100?10??????01?001101?1??00110??????10?10?000?1000000010?1(1/2)?0111?0011???1001001111011?10000?010?001100?000??0000000??01000?0???001?00?10?0????11(0/1)0??2?1100101100??0??1101011100100201000002000(0/1)1010310000?1111100010011?001010000101101000010(0/1)1110?01000001110111?1?01?010????10111?0?0011????0?01?1?1010000010011?10????001?22???0????0???111?????11??01?11???0?010??

***Antetonitrus***

?????????????????????????????????????????????????????????????????????????????????????????????????????????????????????????????0?(0/1)0???????????00?000001??00(1/2)10002010121001000???1?????10?00?01100?0??0?0011??3211000(0/1)0110??????0010?3100???1010???????0010003???002010010001????????????1110111100111111110?11000002100101010?????????????????121111120100?????(1/2)1200?0????(3/4)????????????????????????????????????????11??0?0??000

***Bagualosaurus***

10?1?00(1/2)??0?0???1??????001?000?1000?10??0???0?10010?????????????0?????????????????????????010001010?????01?0010000?00??????????????????????????0??001??0?1???10?0?0000000?0??01??001???????01????0???????????????????????????????????????????????????000011100202?00?00?0?????????????000??0111000101110?1010000000?01??010?????????????????00?01????0?0?10110?????????11?????????101200?1?1???001??1111111100010??100012??01

***Barapasaurus***

?????????????????????????????????????????????????????????????????????????????????????????????????????????????1?11211???????????1?1????00??002??100101??00201102111120121100?0(1/2)10001?1?1?1??1????0????101?00?21???????11?????????????????????????????1111013101000111110011???11110010???????????????????1???1?1?????????1?1???????1??????????20????????????????2?1??????5?????????????????????????????????1??????10-?000?1?0?

***Blikanasaurus***

????????????????????????????????????????????????????????????????????????????????????????????????????????????????????????????????????????????????????????????????????????????????????????????????????????????????????????????????????????????????????????????????????????????????????????????????????????????0000?1110111?1?010010110110111001201111001?1112102120000100?2??????????????????????????????????????????????????0?

***Buriolestes***

000100021010010011000111000000010000100100000?10010010100001?00001?01100110001?10010?1??001100000101000001000100000001010001000000100101110101000000100011?000000000000000000110?00100?0000?000?0?0???1????1011010001?0???????????01????????????????0000011000100?0??0?0010011?000110100000011100010110001100?01010?01?0?1??1?0??????0100???000000?000?0??0100000000??01??00?01000010100011?011110011011000001001001100011100

***Cetiosaurus***

1????????????????????????????????????????????????????????????????????????????????????????????????????????????????????2??0011110111110100100020?2001?1??1020??1(1/2)11102010100???(1/2)??????10111?00100?0??01111010321100100011???????1?????????????????????1111013?020?0110?1?011??1101?00???211112??0???010?111??210100210010111??????????????????????????????????????????????5????????????????????????????????????????1????00?????

***Chindesaurus***

?????????????????????????????????????????????????????????????????????????????????????????????????????????????????????????????0??????????????01?0??0?1??0?1??????000??00?????000????1?0?????1000?0??????????????????????????????????????????????????????????0??001??????0??????????????00001011100010111000??????11111100???01?110100????????????????????????????????????1?????????????????????????????????????????????????00?

***Chromogisaurus***

??????????????????????????????????????????????????????????????????????????????????????????????????????????????????????????????????????????????????????????????????????????????10?00??0000??10????????????????????????02?????????????????????????????0????12100102?????????????????????0??0101110001011000??(0/1)00000?010100010??????????????????????????????????0???000????0??????????????????????????????????????????01?0?2?01?

***Coloradisaurus***

?00?1002??10?111111?1??1110??011000?10??01?10?1011101110000100100?101001111101010011?????1?1001011010011??00010010001?11?001100200100101?01100?00?001?1001100000000000000101?????00?01000?011???0????010010121210011?????????????????1???????????????0?0?13110???011?1100200110010111000001101000010211101110000011101100??011110?001??????0000111111010102000110000210021100?1000?02200?00????0?1111??11111??0?????001?0?00?

**Crurotarsi**

000000000000?0(0/1)000000000?0001000000(0/1)00000000000000000000?0000000000000000000?00000??00000000000000000000000000000000000000000000000???0000??000002000?00?0?00??0000200000000000000000000000000000(0/1)00?0(0/1)0?002?000000000000000000000000000000000000(0/1)000000010?000?0(0/1)00000000000000000000000?00?0000000000000N1?0000(0/1)00000?0000000??0?0000000000?00000000?00?00100000000000(0/1)?(0/1)?01(0/1)0??00?0000?0?(0/1)(0/1)0?(0/1)(0/1)?(0/1)0?000000000??(0/1)??(0/1)00(0/1)?0001

***Efraasia***

100?1001?010?1?111?112?1110?100???00100100000??0?10??01?0??1??????10?00???????100?110??????10010010??11?01100100100001???1?11001101001011011010000001??001?000000000000000001?00000100000?01100000?0?0100112012000111001101012010?110000011101000100001001310010100001000000110010110100001111000010110000010000011101?001?011010?0??01?111000001??0?0?01?1?001(0/1)00001000211001?0????(1/2)20???????????1????11?1????1?00?0?0011???

***Eoraptor***

00010001?010000011000111000000010000110100000?10010010100001?00001101????????????0???????0?000000100000?0?00?100000001?????10000001001011001?10000001000?1100000000000000100?10????1???000110???0?00?110000101100?0010001?0?020000011?00010000000101000001201011?000??0000??11001011010000?011(0/1)0001011000110010101010100?1?010010?00001001?00000?000?0?0?100000000000?010?0??????0010110100?1110010?1100010000?0011?011010001

***Eucnemesaurus***

???????????????????????????????????????????????????????????????????????????????????????????????????????????????????????????????????????????????0???01??001100010000??00?0???????????10000?01100??????????11???????????????????????????????????????????????????????110???0??????????????01101?1010?1010110???000001110110????????????????????????????????????????????????(2/3)???????????????????????????????????????1??0??????00?

***Euparkeria***

00000000?000?0000000?000?100100000000000000000?00000000000010000000000000100?000000?00?000?000000000?100000000000000000?00000000000???0000??000000000?00?0?00??00000000000000000000000?00?0?????0????000?00200000000000??????00000000001?0?0?0000???00?0010?000?0110000000000010000000000?02?000??0100000??1?000?100000?0000000??0?0?0010??0000?0????0?00?1?100000000?000?00?0100000?0000000000?00?100000000?0???11?00000000?

***Glacialisaurus***

???????????????????????????????????????????????????????????????????????????????????????????????????????????????????????????????????????????????????????????????????????????????????????????????????????????????????????????????????????????????????????????????????????????????????????0???????????????0011????????????????0???1010(0 1)0????1100?0111111?1010???????????????????????????????????????????????????????????????????

***Gongxianosaurus***

1?????0?????????12??????????????????????????????????????????????????????????????????????????????????????00???1?2121????????????0????????????1??0??001??0??????0?0??00000????????????????0?0?1?0??000?011???2?110????1?1?????????????????????????????0?100????0????????????????????????(1/2)111?2??????0???110??1??????1?0??1???0?1?1?????1?11??01?0??????1?0??2?1112000020005????????????????????????????????????????????????????

***Guaibasaurus***

???????????????????????????????????????????????????????????????????????????????????????????????????????????????????????????????????????????????0???01??011001000000??00000??0000000?00000?01?0??0(0/1)00??1??????110????1?0???????0????110??0?1?00??0?0?000?01(1/2)10020110010000000010?1011000000100100001011010??100?0?11111100100?01101?0001011100000000000001?011000000000011????????????????????????????????????????1?010012??01

***Herrerasaurus***

00000000?010000000000000?00010000000000100000010001010100000100001101000000101?00?1?0?1000?00000000?100000000000000001010001000000100000?000010000011??0010(0/1)1000000200000010000001011001001100100100?1110??00110000000000000120000001000001101101001000001200000010000010???01?010100100001011100010110000010000110001000100100101000000011000001000000010001000000000?02??0??0?00001101000?111000?1011000000010000?000020001

***Isanosaurus***

?????????????????????????????????????????????????????????????????????????????????????????????????????????????????????????????0??0???????????2??10??0???????????????2?011000?????????????????????????101?0?????????????????????????????????????????????????????????????????????????????211112??0???001?1?011?????????????????????????1???????????????0???????????????????3????????????????????????????????????????????????????

***Jingshanosaurus***

1001?002??1011111100?0?111011101100?102001??1?1011101110?001000????01100010101?2?1??000??1?10110010??1110110010211001????111??010??????1???10100??0011100?1???0000000000000??10???010000000010000000?0010??121201011100??00??0011?3100100?01000102000010013100002011001002??110010110?0100110100001011110??1010001110110?12011010?00???????0120?1112?1?01?2000120000010?4??????????0?2??000?????????????1?1????1?11???001??0?

***Leonerasaurus***

?0??????????????????????????????????????????????????????????????????????????????????????????????0?0???????1?1100110011???001??010?1???????1100?00?0?10000110000000??00000100?111?001?????????????????0100????1100000?????????????????????????????????110103100????????0?????11??1?1????0????????????????????????????????????????????????????100111020????????????0??????????????????????????????????????1111?????1??00001????

***Lessemsaurus***

?????????????????????????????????????????????????????????????????????????????????????????????????????????????????????????????0?011??0?00???101?0000011100(1/2)10000000111000000??????????????????????????0001???21110010110???????0???310?????1?0??1????001001310000?011?10001???1??10110?111011?10???11111?0???00000(0/1)1?01?1???0110110011??????????1?11?????1?2101??00??0???3????????????????????????????????????????11?00000??0?

***Leyesaurus***

?001?00?100011??11?????1010?11010000101101?00?1011111111000100010(0/2)0011?????????00?11110??1?00?10110??1010?111100(0/1)0001?1???111002001001111011????0???????????????????????????????????00000?001????000??????????????????????????????????????????????????????????????????100????????????????????????????????????????????????????????????????010??????????0?102000????0???0???00??1??01022?0000???????1?0????11111???????????????

***Lufengosaurus***

100???02???011?1?11?111111111101001010110100001011101011?0010?1????0110??1010??0001?00?011010010????0111?1101100100011?00011100200110111?011010002001110011000000000000001001101?0010000000(0/1)1000000000110111212(0/1)10111001101111011131001012010001020000100131100020110110021011001011000010110100001021110111010001110110010011110?00001?11100(0/1)0111101010102000110000(1/2)(0/1)004??0????????22000?1?0?000111??0(0/1)111?????1110000011?01

***Mamenchisaurus***

11000113?1110110120000000101112??00111201010101111111012?100000111221010010111?2?1???????2?11(0/1)000010010001111111121113?0001110121010000110002?1110101010020110?011020121000001100111111111011001?0111111000322100100110110011011000100011?0?10?1?31?1111013101?00111110011000110100100211112??0???010?011??21010?0??0??????1010111111??????1120?1????2?1??2?121211102??051?1?0??0100?2?-0-001?---11100001-1-??010?0--0000??00

***Marasuchus***

00???????0?0???????????0?00???????0???????????0???????????????????????????00???0?0?0??????????????????????00?0000000?1?100000000010???00?0??00?000000?00???00??000?00000000?000000000??0010100000000?000?012?1000?0000??????????????????????????????00000000000?01000100000000000000010000000110001000000??00000010001000100100000?0?0000000000?000??0?00?0?00?0??00???10?100100??????0?0?0????????????0?00??????1??000020001

***Massospondylus***

100110021000111111111211011111010000101101000110011011110001000102?01?0011010110001(0/1)??1010010010110101010110(0/1)100110011100?1110020010011110110100020011100110000000000000010111010001(0/1)000000000000000?01(0/1)0111212010111001101111011121001012110000020000100131001000110110010011001011100010110100001021010101000001110110010011110100?01111100001111100?010200011000021002100111000102200000??1(0/1)0011101001111?0?(0/1)111000(0/1)011?0?

**NMQR3314 -'*Melanorosaurus*'**

1001?103100011111101?1110101111???1010101101011001101011000000010220110111010??200110011011101?00111?1000110?1021100110?010110011011010010111?0000001??00(1/2)10000000010000000?0111100110100?0011000????0100??221200000110??????00?0?2100101(1/2)0?000102000010013100001011010001???1?010110?111011110011100111011100000(1/2)1101(0/1)0010011010100100111001(1/2)01111200001??101120000110?3020011000102200001?010-0111??00110111?(0 1)111?00001??0?

***Mussaurus***

????????????????????????????????????????????????????????0????????????????????????????????????????????????????????????????????00100??????10110??0000?1??00100000000?00000010??111??0111?00001110?0????0110??101100011110?1?11?00101210110120100000200???0?0310010?011??1001?011??10110?10101101000010111101?100000(1/2)1101110110110101010??????011011110000011200111000011003?????????????0???????????????????????????1000001??0?

**Neosauropoda**

110001131111011012000001010111(0/2)11101112010101011(0/1)11110121100(0/1)1011222111001011112(0/1)(0/1)0(0/1)000102011100001201000(0/1)11111(0/2)12(0/1)112(0/1)00001110011110100110020121010101(0/1)020111(1/2)0110201211000021000111011111(0/1)1000(0/1)01001110003?21001(0/1)00111100010100000000110001??1?3001111013102000111110011001110(0/1)10(0/1)00211012??0???010?1111121010?010000111110101111111011??11211100??2?10?201212111021105(0/1)(0/1/2)1000(0 1)010022?-?-?01?-?-11?00001?1-??010?0--000???0?

**Neotheropoda**

00(0/1)1(0/1)002(0/1)01001001100001(0/1)000000000000110000000010(0/1)100(0/1)01000001(0/1)0001(0/1/2)010000001011000011110001000000001?0000000000000000101100110000010000(0/1)1000010200001010110010000001000000000(1/2)100001100100000010010000100010?1100000100010001200000010?0001001100(1/2)01010101300011101010000111110000110100001?01100010000001000(0/1)01111111100200101100000111110000??0?0000000?010000000000012?(0/1)?0100000101111(0/1)0000(0/1)10(0/1)?10001000011000010001121000

***Omeisaurus***

11000113111101101200000101011?201001112010101011111010121100000112221110010111???0?????1???11100001?0?0001110111121113??001110120011010111002?1210101011020110?011020121100?021???1?10111100100??0111101000322100100011??????0100001000110001001?3101111013101000111110011000111100100211012??0???010?010??210100?1?0??111?011010?11???1????121?110??2?10?20121211102?105?????????0022?-0-0?1?---11????01111??0101(0/1)-?00001???

**Ornithischia**

001000000000?0000000?000?1011000100100?1000000100100001000000(0/1)00010010000000010000100010010(0/1)01(0/1)00000010011100110100001000000000010100(0/1)0000??010000000?00?0?00?000000000000000(1/2)100000000000010000010000100111(0/2)110000010000000000000000000000000000000011011210010001??000?000110000000100001?0101111020000?000000(0/1)101110002?01?(0/1)1000000111100000?000?00?00?0(0/1)0000000000010?(0/1)?01?00010?0000000000000?0?100111000010(0/1)00100020000

***Pampadromaeus***

000100021010?10?11000?11000??00100001100000????0?10010??0??1?????110?1?????????????????000???000000??0000110010000000??10001????????????1??????0??001??0?1?10?00000000000(0/1)0?0??0?001?0??0??1000???0??1110??10?101010000?????????????????????????????0000011110102?????????0?1?????????00001011100010110?0???00?00???????01??????????????????00000??0???0???1?0???0????????????????000?01111010000?01(0 1)0?11111111000?0010?1?1?1

***Panphagia***

00??????????????????0?1???????????????????0??1????????1?0?0???????10?1?0?1????0????????????1?00001010000?11?010000000????????00100100111?01101?000001??101?10000?000000000??0??0?00??0000?01000?0?0??0111???????????????????????????????????????????00?0?11(0/1)101000?00?000???11??0?110???????????????????????000001010100???0100?01000????????????????0???????0???0?????????????0????????????1?????00111?1011??0??1?1000120?0?

***Pantydraco***

?0???0???????????(0/1)00?0???1??1????000100100000010?100001?00????????00?000?1?10??00?1011?000?00?01010??0000?100100100001001??1100110101001101101?00???1????1????????????????????????0??1?0000100000100?????11?01?00???????????????????????????????????0010012110001?????????0011??10110?00??????????1?1?0000??0000010?01?001??????????????????000011100000101?000000001001?1100??0????2?0????1111???001???111?1011??????101????

***Patagosaurus***

11??????????????????????????????????????????????????????????????????????????????????????????1?00001??????0??011012111??????1100101100000110020?2001010?00201?02111120121?00?0210001110?01?0?100????0?111?00??2100100?11?????????????????????????????111101310100011111001100111110010?211112??0???0011111???10100(0/1)??0???????????????????????????????????????????????????5?????????????????????????????????1?????0?????00?????

***Plateosaurus engelhardti***

100110011010011(0/1)111112111100101100001001010101100110011000110010011011011101011100110010111100101101011111100100100001100111100110100(0/1)01101101000200111001100000000000000101101000010000000010000000001001111120000110011011110101110010020100000100001001311010200101000111110010111100001111000010110001010000111111100100110101000010111001011100000010110011000010003020001000102200001111101?111001111100(0/1)11010(0/1)0101??01

***Plateosaurus gracilis***

?00??001?010?110111?1??1110?101??00?10?1010?0??0?1000110???10??????????????????1????????????0??0110?????1110010010?001?????1100??01???01101101?00?001?100110000000000000010?101000010000000010000?00????????11200?01100?1011?10101010000???10?????0?00100131101020010100011111001011??00001111000010110?0??10000?1??0110??????????????????????0??????????????0?0????????(2 3)?????????1022??????0??0????????1111?????????0101????

***Plateosauravus***

????????????????????????????????????????????????????????????????????????????????????????????????????????????????????????????10???0100101?01101?00(1/2)0011?00110000000001000000??10???011??00??110??0??????0???(2/3)01110011100????????????1????????????????0010013110101?01????????11??10110?0000111100001011000??1000001110110??????0??????????????????110???0?????0??????????3??????????????????????????????????????????10?101??0?

***Pulanesaura***

?????????????????????????????????????????????????????????????????????????????????????????????????????????????10012011????????001?11?0100??11?????0??1??00(1/2)(0/1)?0010?0120001000?????????11111?011???0?0??????????????10??1????????????????????????????????????????????????????0?11??1?11????????????????????????000002100101????????????????????????????????????????1??????????????????????????????????????????1???????????????0?

***Riojasaurus***

1001?00??010011?110??00111011011000?100001000?10010?00110001000001101?0?110101?0001??????1?001000?1??101?10001001000010??1111001101001011011010001001110011000000000000001011101?0010000000110000??0?010011221210011100111111?011111011001010001020?00100131101020110100011011001011001011011101011020110??1000001110110010011010100???011?0010?1110?0?01?21011(0/1)00001??13??0????0?????????????????????????1?????1??(0/1)0010(0/1)??00

***Ruehleia***

????????????????????????????????????????????????????????????????????????????????????????????????????????????????????????????10???0????01???101?002001??00110010000011000000?11000001?0?00?0110???????010011101200011100?0100??01011101000???0??00???00100131100020??11000110110?10110?1010010100001021000??10?00?111011001?000010100????????????????????????????????????3?????????????????????????????????????????10??101??0?

***Saturnalia***

10???????????????????????????????00011000??????????010??0??1???????0?00?11000101?010??????????00000??????0000100000001???????0010010010??01101?000001000011001000000000000000010?0010???0??1000???0??011010101101010102?????????????????????????????0000011100102000000001001100(0/1)011000000(0/1)011(0/1)000101100000000000101010001001001010000100110000000000000100100???000?001010000000?????0??????00???111???100???1??0?01(0/1)0120010

***Seitaad***

???????????????????????????????????????????????????????????????????????????????????????????????????????????????????????????????????????????????00?(0/1)?1??00??00?001????0000???????????????????????????0010011??110001110011?11100101310?00?2110??0???0?????????????0?1000001??????????????????????????????????0?00?11101?0???01101010?00??11?00??11????0?010???11(0/1)0000010???????????????????????????????????????????1??????????

***Shunosaurus***

11000113?11001101200000001011?2010011120101010100110101200001101112101?001111?1200????11020010000012010001111112121112??01011?1001?1?10010012?1100001011021?0??01??201210000?11??00?101?110110011?111?1?0002221001100?11000?1011001?000110000??1?310111101310100011111?01100?111100100211112??0???000?011??21010?(0/1)1?0????110?101???1?1011??1121?110??2?00?2?12121?1021105???0??1?10022?-?-?01?---11?00?10?1???01111--0000????

***Silesaurus***

00?0??00?000?00100000??0?00?0?01010?????????0010??0????0??????????00?0?0?1000??00011?????1?00?0?010???0?000001001000010?00000000000???0010000000000010100001100000000000000??100000100000?0?0????????111?11100000100000?????????????????????????????00000100001001000000000?010100?0?1000011011000100100000100011110000000?0000000??011?1??002???00000000?0?10???000?00111100000??00?0000000???00?????101101??0?00?1000000000

***Spinophorosaurus***

1????1????????????????????????????????2????????????0?????1?????????0??1011111112?1??????????1?????????????10011012?112?????11?11011?0?01?0002?110??01????(1/2)?11???1?12?1010?0?????????1???11011?001?1??1110?03?2(1/2)0?10??????????????????????????????????????????(1/2)???1?1???01(1/2)0?011??001??211111110?1101??0?0??20?1002??0??11?101?0?0?01??????????????????????????????????????21?0?101?????????????????1??????1?????0??-?????????

***Staurikosaurus***

00?????????????????????????????????????????????????????????????????????????????????????????0000000001000?00?000000000????????00010????00?01001?000011?000?00000000020000001??010?101100?001100100????1?1????????????????????????????????????????????00000020010000110?010?00010?00000100001??000?0101?000??000001100000001????01????????????????????????????????????????1?????????????????????????????????0???0?0???000021000

***Tazoudasaurus***

11?????????????????????????????????????????????????010????????????(0/2)011???1???1?????????????00?00000???0??10?111012101???000??001010??????0002??100001??00200101011020121000?????????11?11?111?????11?????01?22100100??????????010?210000100?0001?310?1???03101?0?111000002??11??1??1??21101111001111011?0???0010020101010?1111011111??011????1?????20????????1??10?1????5???????????????????????????01??0111??????1--00001?0?

***Thecodontosaurus***

?0???????????????????????1??1??1000??????0?0?0?0???000????????????10?0?0?1010?10001????????00?11010??????110010010000?1??????000101010?1?01101?000001??00110000000000000000?10000?0101000?01000?0?00?010011201100010100110001201000110000101000001010000012100001???0?000??011??01010?0000111100001011000??10000010101100??011010100?????1100?0?111??0??1??000??00?0????1?000?10????????????????????????0110??1??10??0???????

***Unaysaurus***

100110011010?1?111??1??1010?1011000????????????0??00????001???000??0?10????10??10?11??????010?10110?????0110010010000???0?1?????0?1?0??????????0??0?11?00?10000?0??00000?????????????0?0???010000?00?010011?11200001100??????1????110????211???0??0???????????????????????????????????????????????????????????????110110???011010?00????????0???1100??????0??0???0??????10?0??10????1?01?011???0???1?10?0111?????01????????0?

***Vulcanodon***

?????????????????????????????????????????????????????????????????????????????????????????????????????????????????????????????????????????????????????????(1/2)????????????????????1??????01?11?0?00??0?0???1???2?(1/2)10?10?011???????????????????????????????????310(1/2)00?01111000100111110010?111011110010011?1????20?10021?0???11?10101101111011??111?10102?1001020111110?121?05????????????????????????????????????????1????000????

***Yunnanosaurus***

100?1002??00?10011001??1011111????00101110?00??0?1101111?001000001?01?0?01010??????????????10000010???0???0001021000?1???111100(0/1)10110100?0110100000011000110000000001000000?0101?10100000?00?0???0??001?0??121200?11100??01??0011?2101100201000(0/1)020?00100131000000110100010?110010111?00101101000010110101?101000110011001?011010100001111001?0?1110?0?011???11?0000110?2??????????0????0?????????11???00-?-11??111??0001??01
